# Functional group introduction and aromatic unit variation in a set of π-conjugated macrocycles: revealing the central role of local and global aromaticity†

**DOI:** 10.1039/d1qo00901j

**Published:** 2021-06-22

**Authors:** Martina Rimmele, Wojciech Nogala, Maryam Seif-Eddine, Maxie M. Roessler, Martin Heeney, Felix Plasser, Florian Glöcklhofer

**Affiliations:** Department of Chemistry, Imperial College London London W12 0BZ UK f.glocklhofer@imperial.ac.uk; Centre for Processable Electronics, Imperial College London London W12 0BZ UK; Institute of Physical Chemistry, Polish Academy of Sciences Kasprzaka 44/52 01-224 Warsaw Poland; Department of Chemistry, Loughborough University Loughborough LE11 3TU UK f.plasser@lboro.ac.uk

## Abstract

π-Conjugated macrocycles are molecules with unique properties that are increasingly exploited for applications and the question of whether they can sustain global aromatic or antiaromatic ring currents is particularly intriguing. However, there are only a small number of experimental studies that investigate how the properties of π-conjugated macrocycles evolve with systematic structural changes. Here, we present such a systematic experimental study of a set of [2.2.2.2]cyclophanetetraenes, all with formally Hückel antiaromatic ground states, and combine it with an in-depth computational analysis. The study reveals the central role of local and global aromaticity for rationalizing the observed optoelectronic properties, ranging from extremely large Stokes shifts of up to 1.6 eV to reversible fourfold reduction, a highly useful feature for charge storage/accumulation applications. A recently developed method for the visualization of chemical shielding tensors (VIST) is applied to provide unique insight into local and global ring currents occurring in different planes along the macrocycle. Conformational changes as a result of the structural variations can further explain some of the observations. The study contributes to the development of structure–property relationships and molecular design guidelines and will help to understand, rationalize, and predict the properties of other π-conjugated macrocycles. It will also assist in the design of macrocycle-based supramolecular elements with defined properties.

## Abstract 2

Insights into how the optoelectronic properties of π-conjugated macrocycles evolve as a result of systematic structural changes are presented, helping to develop structure–property relationships and molecular design guidelines.

## Graphical abstract

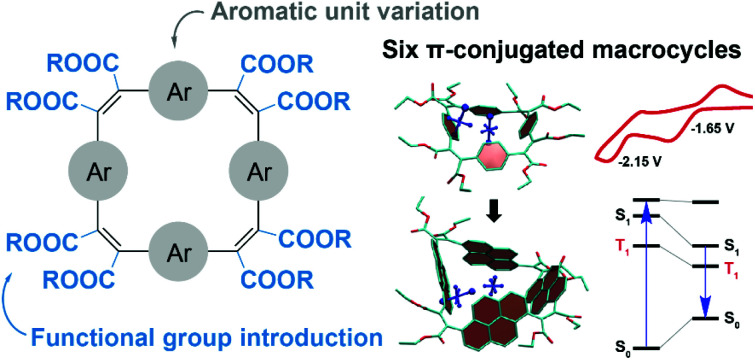

## Introduction

π-Conjugated macrocycles provide an exciting playing field for the discovery of effects and properties that cannot usually be attained with organic compounds. Their study began in the early 1960s and many π-conjugated macrocycles have been synthesized since.^1^ However, only recently the field has moved towards making use of the unique properties of these cyclic molecules for applications. π-Conjugated macrocycles are now being investigated in organic solar cells,^2^ photodetectors,^3^ field-effect transistors,^4–7^ and light-emitting diodes.^8–11^ They can also be used in bioimaging,^12^ as templates for the growth of carbon nanotubes,^13^ and as molecular nanoreactors.^14^ Moreover, macrocycles can serve as (non-natural) supramolecular elements.^15^

**Scheme 1:**
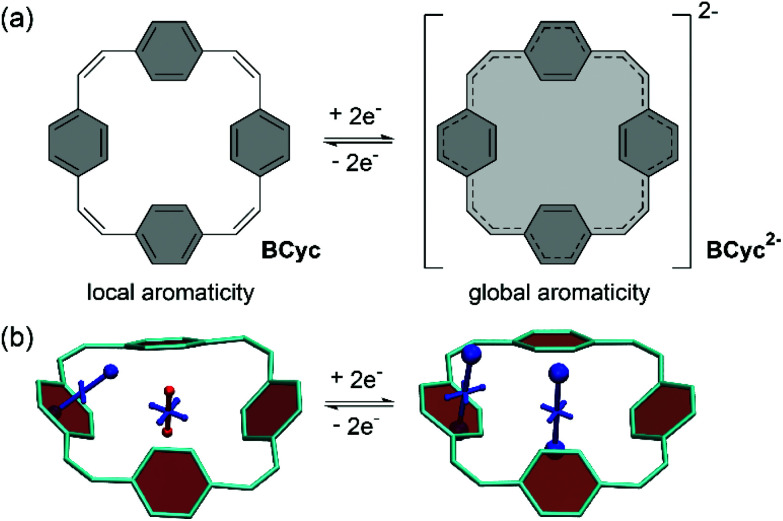
(a) Reversible two-electron reduction and aromaticity switching of the π-conjugated macrocycle [2.2.2.2]paracyclophane-1,9,17,25-tetraene, denoted **BCyc** here. (b) Visualization of chemical shielding tensors (VIST) with shielded (aromatic) contributions in blue and deshielded (antiaromatic) contributions in red. The VIST plots show that the neutral ground state is dominated by the local aromaticity of the phenylene units; the formally antiaromatic perimeter of [4*n*] π-electrons only induces weak global antiaromaticity. The doubly reduced state, however, is dominated by the global aromaticity of the resulting [4*n* + 2] π-electron perimeter. Hydrogen atoms omitted for clarity.

In our own research, we exploited the capability of [2.2.2.2]paracyclophane-1,9,17,25-tetraene, a [2.2.2.2]cyclophanetetraene with benzene units, denoted **BCyc** here, to switch between a locally aromatic neutral state and a globally aromatic doubly reduced state (Scheme 1).^16,17^ This switching enabled excellent redox properties and application as high-performance organic sodium-ion battery anode material. The macrocyclic geometry was further found to result in voids in the solid-state packing that can facilitate ion diffusion.

Further applications of π-conjugated macrocycles are possible if the molecular structure is adapted accordingly. However, there are only a small number of experimental studies that investigate how the properties of π-conjugated macrocycles evolve with systematic structural changes, presumably due to the difficulties of synthesizing sets of macrocycles that allow for such an investigation. Cycloparaphenylenes (CPPs) are probably the class of π-conjugated macrocycles for which the most extensive structural variations have been performed and several applications are now emerging.^18,19^ However, in contrast to **BCyc**, the π-system of these compounds, which have been synthesized over a period of more than a decade, is radially oriented, which makes it difficult to draw conclusions for other macrocyclic π-systems. We believe that further systematic studies of π-conjugated macrocycles are needed to gain a better understanding of the evolving properties. Such studies can also be expected to lead to the discovery and identification of unexpected effects and properties, which may ultimately result in the development of new materials and applications.

**Scheme 2:**
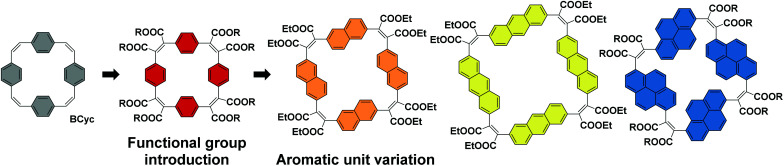
Systematic structural changes to the π-conjugated macrocycle **BCyc** to study the evolving properties in a set of [2.2.2.2]cyclophanetetraenes: (i) introduction of electron-withdrawing ester groups and (ii) replacement of the benzene units by naphthalene, anthracene, and pyrene units.

For the study presented here, we aimed to investigate how the introduction of electron-withdrawing groups at the vinylene bridges of **BCyc** can influence its properties. We were particularly interested in how this structural modification changes the redox properties of the compound. While the reduction of **BCyc** was found to be a two-electron process and the charges of **BCyc2−** were delocalized, electron-withdrawing groups were expected to impact on this behavior, possibly enabling reduction beyond the dianion. Due to the availability of promising synthetic methods, we aimed to introduce ester groups as the electron-withdrawing groups to study these effects (Scheme 2, Functional group introduction). In the context of supramolecular elements (and considering that macrocycles can serve as such elements), it is noteworthy that these functional groups will point to the outside of the macrocycle due to their position at the vinylene bridges and conformational restrictions, impacting the (intermolecular) non-covalent interactions.

In addition to the functional group introduction, we were curious how a variation of the aromatic units can affect the properties, replacing the benzene units of the ester-substituted macrocycle by naphthalene, anthracene, and pyrene units (Scheme 2, Aromatic unit variation). This variation gradually increases the size of the macrocycles, yielding a set of ester-substituted [2.2.2.2]cyclophanetetraenes (all with a formally antiaromatic macrocyclic system of [4*n*] π-electrons in the neutral ground state), and was expected to result in changes to the effective ring currents and the associated local and global (anti)aromaticity in the neutral and charged states. Nevertheless, our interest here was also to identify deviations from the expected trends to advance our understanding of the unique properties that can be attained with carefully designed π-conjugated macrocycles. In contrast to the ester groups, which may serve as hydrogen bond acceptors or in dipole–dipole interactions, the larger aromatic units can strengthen π–π interactions or cation–π interactions; the resulting larger cavity may be able to host larger guest molecules or ions.

Our aim was to combine this experimental investigation with an in-depth computational analysis to explain, understand, and confirm the experimental results and to evaluate computational tools and methods that can facilitate the study of π-conjugated macrocycles. Aside from reproducing experimental energies, it was of particular interest to quantify and visualize the local and global (anti)aromaticity of these macrocycles. Generally speaking, a wide array of aromaticity descriptors has been developed relying, *e.g.*, on structural^20^ or electronic^21^ properties. A particularly popular set revolves around magnetic properties and induced currents,^22–27^ as these are related to the characteristic signals of (anti)aromatic molecules observed in nuclear magnetic resonance (NMR) spectroscopy. For the present study, we used a newly developed method for the visualization of chemical shielding tensors (VIST).^17^ VIST is based on the nucleus-independent chemical shift (NICS),^28^ however, rather than just reporting a number, VIST shows the full local shielding tensor graphically. It is, thus, particularly suitable for illustrating the dramatic changes in the electronic structure the molecules studied undergo when their charge and spin state is altered.

## Results and discussion

### Synthesis

**Scheme 3:**
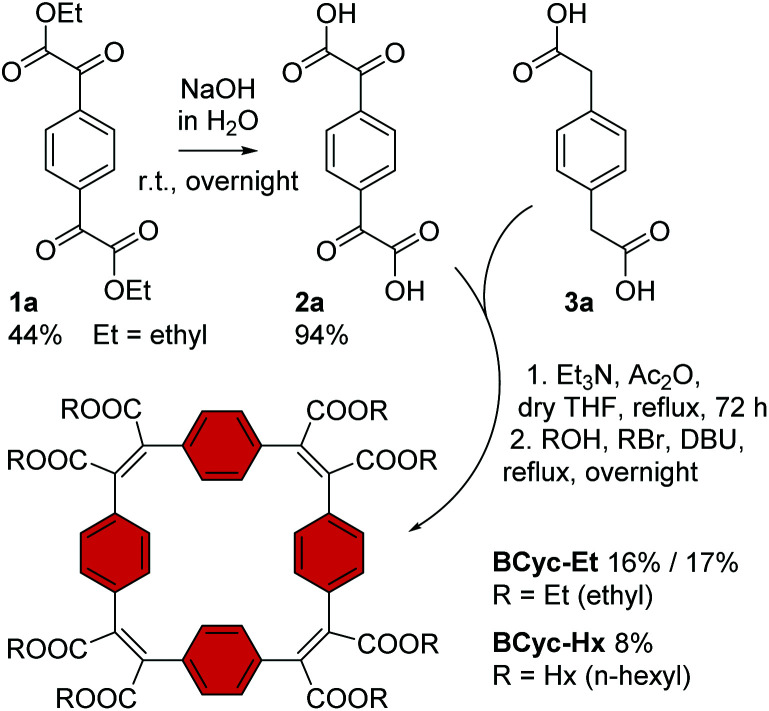
Synthesis of macrocycles **BCyc-Et** and **BCyc-Hx** with benzene units and ethyl/hexyl ester groups. Compound **1a** was obtained from 1,4-diiodobenzene (ESI section 1.3.1†).

In contrast to the synthesis of the unsubstituted macrocycle **BCyc** (Scheme 1), which can be achieved by a Wittig reaction of terephthalaldehyde with *p*-xylylenebis(triphenylphosphonium bromide),^16^ the synthesis of the π-conjugated macrocycles with ester groups at the vinylene bridges requires α,α′-dioxoarylenediacetic acids (also known as arylenediglyoxylic acids) and arylenediacetic acids as the precursors.^29,30^ These precursors, such as compounds **2a** and **3a** in Scheme 3, can give ester-substituted macrocycles in a two-step reaction. In the first step, a Perkin-type condensation reaction of the precursors gives macrocyclic carboxylic anhydride intermediates, which are then converted into the ester-substituted macrocycles by addition of an alcohol and a bromoalkane in the second step. In this study, we either added ethanol and bromoethane or 1-hexanol and 1-bromohexane for the second step of the reaction, yielding ethyl or hexyl ester-substituted macrocycles, respectively.

For the synthesis of the macrocycles with benzene units, **BCyc-Et** and **BCyc-Hx** (Scheme 3), α^1^,α^4^-dioxo-1,4-benzenediacetic acid **2a** was obtained in two steps starting from 1,4-diiodobenzene. In the first step, lithium–halogen exchange and quenching with an excess of diethyl oxalate gave compound **1a**, which was then saponified to yield precursor **2a** in an overall yield of 42%. An alternative one-step approach, Riley oxidation of 1,4-diacetylbenzene under conditions previously used for the oxidation of acetophenone,^31^ also gave precursor **2a** according to ^1^H NMR spectra. However, our attempts to separate the highly polar product from the by-products of this alternative approach were not successful. The second cyclisation precursor, 1,4-benzenediacetic acid **3a**, is available from commercial suppliers.

Using precursors **2a** and **3a**, **BCyc-Et** and **BCyc-Hx** were obtained by adapting a protocol reported for the synthesis of the corresponding π-conjugated macrocycle with pyrene units, which was shown to assemble into a tubular structure.^29^ In contrast to the previous report, the macrocycles were purified by gel permeation chromatography (GPC) instead of silica gel column chromatography, enabling efficient separation of oligomeric by-products and, thus, helping to prevent an overestimation of the reaction yield. After purification, **BCyc-Et** was obtained as a yellow solid in a yield of 16%; the initially obtained oil slowly crystallized to give the yellow solid. A marginally higher yield of 17% was achieved when the precursors were dissolved and added to the reaction over a period of 10 hours using a syringe pump. Such slow addition can keep the concentration of the precursors in the reaction low, which is usually considered to favor cyclisation over the formation of linear oligomers, but the effect was negligible in our case. The macrocycle with hexyl ester groups, **BCyc-Hx**, was obtained as a dark oil in a lower yield of 8%. In contrast to **BCyc-Et**, this macrocycle did not crystallize, and the separation of the by-products was found to be more challenging, despite using GPC for purification.

**Scheme 4:**
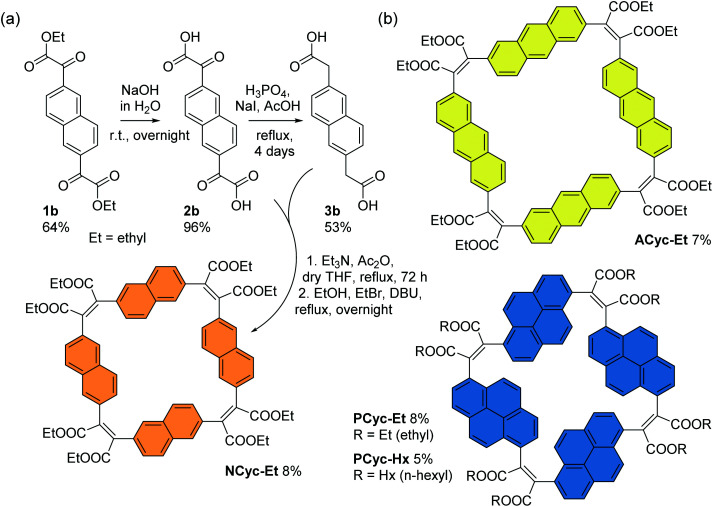
(a) Synthesis of macrocycle **NCyc-Et** with naphthalene units and ethyl ester groups. Compound **1b** was obtained from 2,6-dibromonaphthalene (ESI section 1.3.2†). (b) Macrocycles **ACyc-Et**, **PCyc-Et**, and **PCyc-Hx** with anthracene/pyrene units and ethyl/hexyl ester groups, obtained in analogous reactions using precursors **2c–d** and **3c–d** (ESI sections 1.3–1.6, Fig. S1†).

For the corresponding macrocycles with larger aromatic units, **NCyc-Et**, **ACyc-Et**, **PCyc-Et** and **PCyc-Hx** (Scheme 4 and ESI Fig. S1†), the required α,α′-dioxoarylenediacetic acid precursors **2b–d** were obtained in similar reactions as used for the synthesis of precursor **2a**. However, dibrominated instead of diiodinated arenes were used as the starting materials for the synthesis of compounds **1b–d** and the reaction conditions and reagents of both synthetic steps were adapted and optimized for each precursor. In contrast to precursor **3a**, the arylenediacetic acid precursors **3b–d** are not commercially available but were synthesized by reduction of compounds **2b–d**, adapting previously reported conditions.^29,32^ Interestingly, the corresponding reduction of substituents in the 9,10-positions of anthracene did not give the desired product (ESI Fig. S2†).

The macrocycles were then prepared under the same conditions as **BCyc-Et**, yielding **NCyc-Et** as a pale yellow solid, **ACyc-Et** and **PCyc-Et** as orange solids, and **PCyc-Hx** as a red oil. No syringe pumps were used due to poor solubility of the precursors in the reaction solvent and the negligible effect on the reaction yield in the synthesis of **BCyc-Et**. As intended, the introduction of hexyl ester groups in **PCyc-Hx**, a reaction tested with the synthesis of **BCyc-Hx**, significantly improved the solubility of the compound compared to **PCyc-Et**, but the separation of the reaction by-products was again found to be tedious. The macrocycles were characterized by ^1^H and ^13^C NMR as well as by high-resolution mass spectrometry (HRMS) to confirm the molecular structure. Unfortunately, although we found the synthesis of all precursors and all other macrocycles to be reproducible, we struggled to re-synthesize **ACyc-Et** in later attempts. However, as highly interesting effects were observed for this macrocycle in both the computations and experiments, we do not want to omit the available data for this macrocycle here. The computational analysis also allows us to present a possible explanation for the difficulties with the synthesis of this compound related to its more flexible nature.

### UV-vis absorption and photoluminescence

**Fig. 1:**
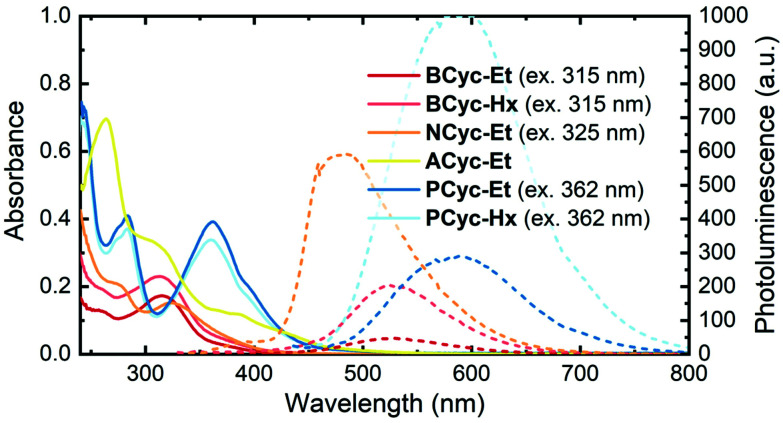
UV-vis absorption (solid lines) and photoluminescence spectra (dashed lines) of the macrocycles in CHCl_3_ solution (5 μM). The excitation wavelengths for recording the photoluminescence (PL) spectra are shown in brackets; spectra obtained at other excitation wavelengths are available (ESI Fig. S43†).

UV-vis absorption spectra (Fig. 1, solid lines) in CHCl_3_ solution showed a peak at 315 nm for **BCyc-Et** and 313 nm for **BCyc-Hx**, both slightly redshifted compared to the unsubstituted macrocycle **BCyc**, which showed an absorption peak at 306 nm in the same solvent.^16^ In contrast to **BCyc**, an additional shoulder at approx. 262 nm was observed for both macrocycles. Considering that **BCyc-Et** and **BCyc-Hx** feature the same π-conjugated system, the similarity of their absorption spectra is not surprising. The photoluminescence (PL) spectra (Fig. 1, dashed lines) also showed similar peak wavelengths, 526 nm for **BCyc-Et** and 530 nm for **BCyc-Hx**. The difference between the absorption and PL peak wavelengths corresponds to large Stokes shifts of 1.58 and 1.62 eV, respectively. Interestingly, despite featuring the same π-conjugated system, the PL was found to be significantly more intense for the hexyl-ester substituted macrocycle **BCyc-Hx**, possibly due to shielding of the chromophores by the hexyl chains.

With both **NCyc-Et** and **ACyc-Et**, a trend to further redshifted absorption is observed. **NCyc-Et** showed an absorption peak at 324 nm and a shoulder at 274 nm. The PL maximum was found to be at 486 nm, corresponding to a Stokes shift of 1.28 eV, smaller than for **BCyc-Et/Hx**. **ACyc-Et** showed an absorption peak at 264 nm and shoulders at longer wavelengths; PL data for **ACyc-Et** is not available. The absorption spectrum of **ACyc-Et** has a markedly different shape compared to the other molecules studied and we assign this difference to its folded structure and the ensuing loss of symmetry that are discussed below.

As for **BCyc-Et** and **BCyc-Hx**, the absorption spectra of **PCyc-Et** and **PCyc-Hx** were found to be very similar, showing peaks at 283 nm and 362/360 nm. The PL was again significantly more intense for the hexyl-ester substituted compound, while the peak wavelengths were similar, 588 nm for **PCyc-Et** and 593 nm for **PCyc-Hx**. This corresponds to Stokes shifts of 1.32 eV and 1.35 eV, respectively, similar to the Stokes shift observed for **NCyc-Et**.

Beside the experimental results discussed so far, Table 1 presents results computed at the time-dependent density functional theory (TDDFT) level. The first computed singlet state of **BCyc-Et**, located at 353 nm, is symmetry forbidden within the four-fold symmetry of this molecule. This state may be tentatively assigned to the red tail between 350 and 400 nm observed in the experimental spectrum. A degenerate pair of bright states (oscillator strength of 0.77) follows at 315 nm, in good agreement with the experimentally observed absorption maximum. These three states, along with the dark S_4_ state (not listed in Table 1), are characterized by an excitation from a π-orbital delocalized over the whole system into the π*-orbitals on the vinylene units, with some contributions by the ester groups. Next, a degenerate pair of bright states (oscillator strength of 0.40) at 294 nm is found, which is consistent with the higher-energy shoulder in the spectrum.

**Table 1:** Experimental and theoretical UV-vis absorption and photoluminescence bands of the macrocycles in solution and the experimental Stokes shifts

Compound	*λ*_abs., exp._ a [nm]	*λ*_abs., th._ [nm]	*λ*_PL, exp._ [nm]	*λ*_PL, th._ c [nm]	Stokes shift [eV]
**BCyc-Et**	262/**315**	294/**315**/353	526	648	1.58
**BCyc-Hx**	262/**313**	n/a	530	n/a	1.62
**NCyc-Et**	274/**324**	342/**353**/379	486	450	1.28
**ACyc-Et**	**264**/307/387	**451**/476/496	n/a	592	n/a
**PCyc-Et**	275/**283**/**362**	**398**/431	588	580	1.32
**PCyc-Hx**	275/**283**/**360**	n/a	593^b^	n/a	1.35

a Wavelength of maxima shown in bold, wavelength of shoulders estimated.

b Determined from the photoluminescence spectrum recorded with the detector voltage set to ‘medium’ (ESI Fig. S44†).

c Computed at the optimized triplet geometry in dichloroethane (DCE).

Proceeding to **NCyc-Et** and **PCyc-Et**, both of which retain the four-fold symmetry, one finds the same structure with a dark low-energy state and a pair of bright degenerate states giving rise to the main peak in the spectrum. In agreement with the experiments, we find a consecutive red shift when going to **NCyc-Et** and further to **PCyc-Et**, but this shift is somewhat overestimated by the computations. For the bright pair of states in **NCyc-Et** and **PCyc-Et** we find oscillator strengths of 0.61 and 1.20, respectively, showing that the computed oscillator strengths are in good agreement with the relative experimental intensities. A discussion of **ACyc-Et** is more challenging considering that more states are involved due to the loss of symmetry in this molecule (as a result of the folded structure discussed below). We find two dark states (496 and 476 nm) before the first bright state located at 451 nm. Due to the lack of symmetry, this state is not degenerate, and we find a second bright state at a slightly higher energy (438 nm). We tentatively assign these two states to the extended red tail found between 400 and 500 nm in the absorption spectrum of **ACyc-Et**.

### Electrochemistry

**Fig. 2:**
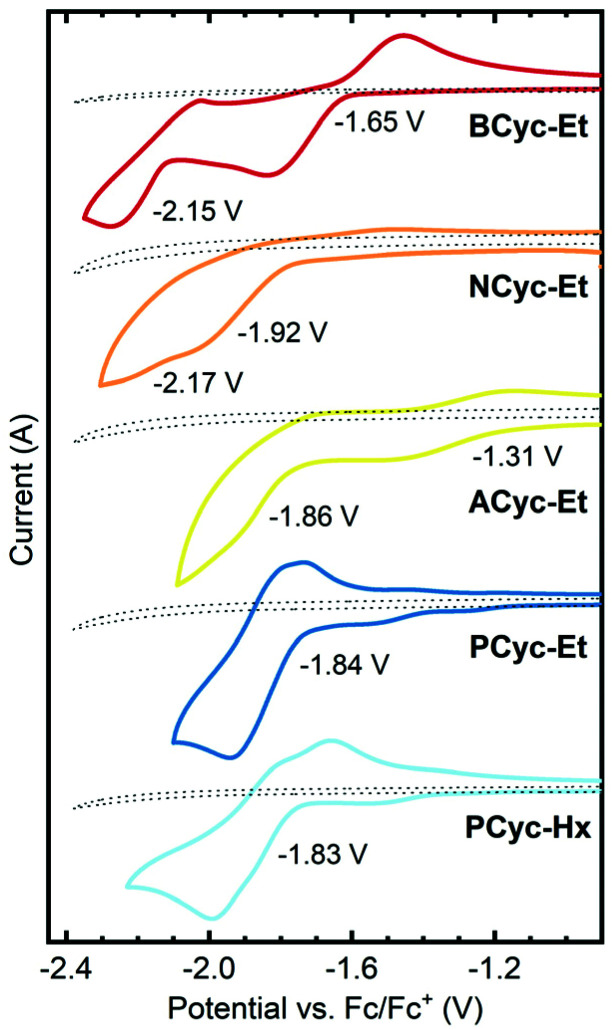
Cyclic voltammograms and redox potentials for the reduction of the macrocycles in dichloroethane (DCE), recorded on 2 mm diameter platinum disk electrodes at a scan rate of 0.1 V s^−1^ with 0.1 M NBu_4_PF_6_ as the supporting electrolyte. Dotted lines show the measurement of the electrolyte solution without macrocycle. Previously reported redox potential of **BCyc** in DCE for comparison: −2.29 V *vs.* ferrocene/ferrocene^+^ (Fc/Fc^+^).^16^

π-Conjugated macrocycles are potentially interesting compounds for battery electrodes and other electrochemical applications, as we have shown in our investigation of **BCyc**.^16^ Cyclic voltammetry (CV) measurements (Fig. 2) were expected to give insights into how the electrochemical properties evolve with the structural changes in our set of macrocycles. These measurements revealed a large shift of the first reduction wave upon introduction of the electron-withdrawing ester groups, from −2.29 V *vs.* ferrocene/ferrocene^+^ (Fc/Fc^+^) for **BCyc** to −1.65 V for **BCyc-Et**. In both cases, the reduction was found to be chemically reversible. However, in contrast to **BCyc**, a second reduction wave was observed for **BCyc-Et** within the electrochemical window of the electrolyte solution, at a potential of −2.15 V.

Measuring cyclic voltammograms for **BCyc-Et** at defined concentrations in different solvents, we aimed to determine the stoichiometry of the observed reduction steps (as we did previously for **BCyc**). However, due to sluggish kinetics of electron transfer to the ester-substituted macrocycles, the analysis of the voltammograms could neither confirm that the reduction proceeds in two one-electron steps nor exclude the presence of two two-electron steps (see ESI section 4.2†).

Upon variation of the aromatic units, a shift of the reduction to lower potential was observed for **NCyc-Et**; a redox potential of −1.92 V was determined for this compound. The voltammogram (and more clearly its 1^st^ derivative, see ESI Fig. S45†) indicates that there may be a second reduction step at −2.17 V. In contrast to **BCyc-Et**, the reduction was found to be chemically irreversible. The lower redox potential for the reduction of **NCyc-Et** (compared to **BCyc-Et**) may be explained by its weaker global antiaromaticity in the neutral state and weaker global aromaticity in the doubly reduced state, discussed further in the section on ring currents and chemical shielding below. Interestingly, further variation of the aromatic units led to unexpected effects. Chemically reversible reduction at a significantly higher potential of −1.31 V was observed for **ACyc-Et**, while for **PCyc-Et** and **PCyc-Hx** the reduction remained at a similar potential as for **NCyc-Et** (at −1.84 V and −1.83 V, respectively), albeit being chemically reversible. For **ACyc-Et**, a second reduction wave was observed at −1.86 V.

**Fig. 3:**
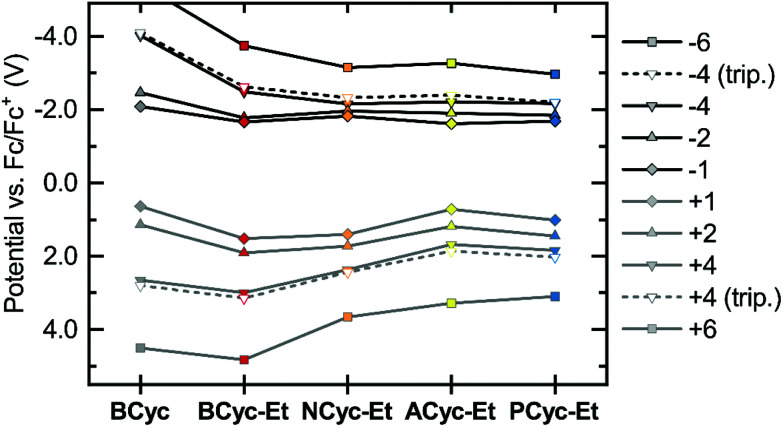
Calculated redox potentials *vs.* Fc/Fc^+^ for the reduction to the mono-, di-, tetra- and hexaanions (top) and oxidation to the corresponding cations (bottom) of the macrocycles in 1,2-dichloroethane (DCE). Data available in the ESI Table S2.†

Considering the relatively small structural differences between **ACyc-Et** and the most similar other macrocycles, **NCyc-Et** and **PCyc-Et**, as well as the absence of a general trend to serve as an explanation, rationalizing the observed redox potentials was difficult. Ruling out impurities as an explanation, we were curious if a computational investigation of the electrochemical properties could validate the results. Hence, we computed the redox potentials by optimizing the molecular geometries for the individual charged states using density functional theory and computing their Gibbs free energies in DCE solution (see computational details). Indeed, the computations showed a dip in the redox potential for the first reduction wave of **ACyc-Et** (Fig. 3, top), but the dip was smaller than in the experiments.

The computations also showed very small differences in redox potential between the first and second reduction of all ester-substituted macrocycles, except for **ACyc-Et**. Furthermore, they revealed that the introduction of ester groups significantly facilitates the fourfold and sixfold reduction of the macrocycles; the redox potentials for the reduction to the tetra-/hexaanions shifted from −4.01/−5.43 V for **BCyc** to −2.48/−3.74 V for **BCyc-Et**. These results strongly support that the reduction steps of **BCyc-Et** observed in the CV measurements are two consecutive two-electron processes, corresponding to a reversible reduction to the dianion in the first step and to the tetraanion in the second step. For **NCyc-Et**, the potential differences were found to be even smaller, with the calculations indicating a potential of −1.82 V for the first reduction, −1.96 V for the second reduction, and −2.16 V for the reduction to the tetraanion. As all calculated potentials (ESI Table S2†), these values corresponded well with the experimentally determined redox potentials.

While the introduction of the ester groups has its most pronounced effect on the reduction to the tetra- and hexaanions, the variation of the aromatic units most significantly affects the oxidation to the tetra-/hexacations (Fig. 3, bottom), shifting the corresponding potentials from 3.00/4.83 V for **BCyc-Et** to 1.68/3.28 V for **ACyc-Et**. In line with the calculations, the experimentally determined redox potentials for the first oxidation wave showed a similar, albeit smaller shift from 1.47 V for **BCyc-Et** to 0.85 V for **ACyc-Et** (ESI Fig. S45†). For all ester-substituted macrocycles, the oxidation was found to be chemically irreversible in the CV measurements. In contrast, the two-electron oxidation of **BCyc** at 0.77 V was shown to be chemically reversible.^16^

In addition to the redox potentials for the singlet states of the macrocycles discussed so far, we also calculated the redox potentials for the reduction and oxidation to the tetraanions and -cations in the triplet state (Fig. 3, dashed lines). These calculations showed surprisingly small differences between the singlet and triplet states, which are discussed further below in the section on the ring currents and chemical shielding.

### Conformation

**Fig. 4:**
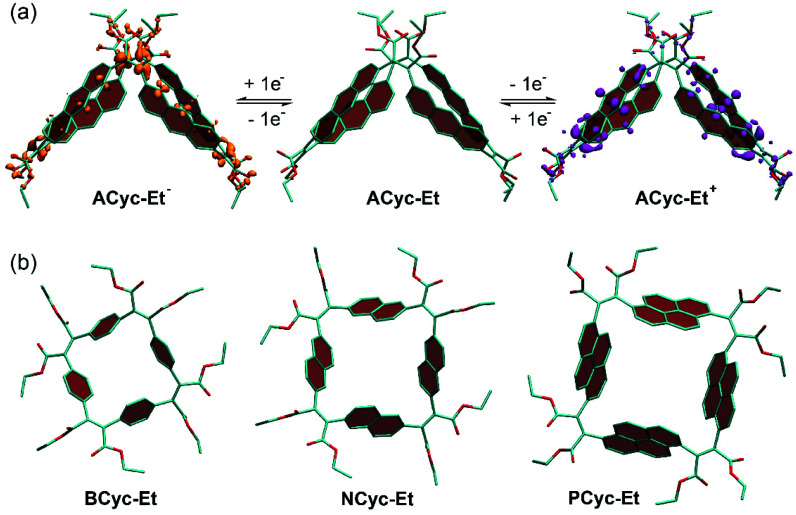
Calculated conformation of (a) **ACyc-Et** in the neutral and singly charged states and (b) **BCyc-Et**, **NCyc-Et**, and **PCyc-Et** in the neutral state. Panel (a) shows electron density differences between the neutral and charged states (isovalues −0.0015*e* for **ACyc-Et−** and +0.0015*e* for **ACyc-Et+**). Hydrogen atoms omitted for clarity.

π-Conjugated macrocycles are often shape-persistent, meaning they are non-collapsible, geometrically well-defined, and conformationally restricted. We also expected the macrocycles from this study to be shape-persistent, but – when looking into the origin of the higher redox potential for the first reduction and the lower redox potential for the first oxidation of **ACyc-Et** compared to the other macrocycles (an effect observed in the calculations and experiments) – enhanced structural flexibility of the macrocycle was found to provide an explanation for the effect. As our calculations show, the macrocycle features a collapsed conformation in the neutral as well as in the first charged states (Fig. 4a), enabling increased stabilization of the injected charges and resulting in the observed shift of the redox potentials. Fig. 4a also shows electron density difference plots for the singly charged states of **ACyc-Et**, highlighting that the excess electron in the anion is predominantly located in the vicinity of the electron-withdrawing ester groups, whereas the hole in the cation is distributed over the aromatic units. The collapsed conformation also helps to explain the UV-vis absorption spectrum of the macrocycle as it reduces the symmetry, enabling lower energy transitions. In contrast to **ACyc-Et**, the more rigid nature of the other macrocycles seems to prevent a collapse (Fig. 4b).

Interestingly, the more flexible nature of **ACyc-Et** can also provide an explanation for the difficulties with its synthesis. Conformation plays a crucial role in cyclization reactions, as it has a strong impact on the degree of competing oligomerization and polymerization reactions; an unfavorable conformation of the intermediates can impede the formation of macrocycles. Greater flexibility will make the conformation of the intermediates more prone to subtle changes in reaction conditions or impurities, explaining our results for the synthesis of **ACyc-Et**.

### EPR spectroelectrochemistry

In order to clarify whether the two reduction steps observed in the CV measurements of **BCyc-Et** are one-electron steps (as cannot be excluded based on the measured voltammograms) or two-electron steps (as supported by the calculation of the redox potentials), electron paramagnetic resonance (EPR) spectroelectrochemical measurements were performed (see ESI section 5†). A reduction proceeding in two one-electron steps would result in the formation of a paramagnetic species and, consequently, in an EPR signal after the first reduction step. The signal would disappear again in the second step. If the reduction proceeds in two two-electron steps, no paramagnetic species is formed at any point of the reduction cycle and no EPR signal is expected.

Indeed, no EPR signal was observed at any point in the measurement of **BCyc-Et** (ESI Fig. S51†), corroborating the reduction to the dianion in the first step and to the tetraanion in the second step. In contrast, measurements of the paramagnetic compound 4-amino-2,2,6,6-tetramethylpiperidinyl-1-oxyl (4-amino-TEMPO) showed an EPR signal (ESI Fig. S50†), confirming that the absence of a signal in the measurements of **BCyc-Et** cannot be attributed to issues with the measurement set-up.

### Ring currents and chemical shielding

**Fig. 5:**
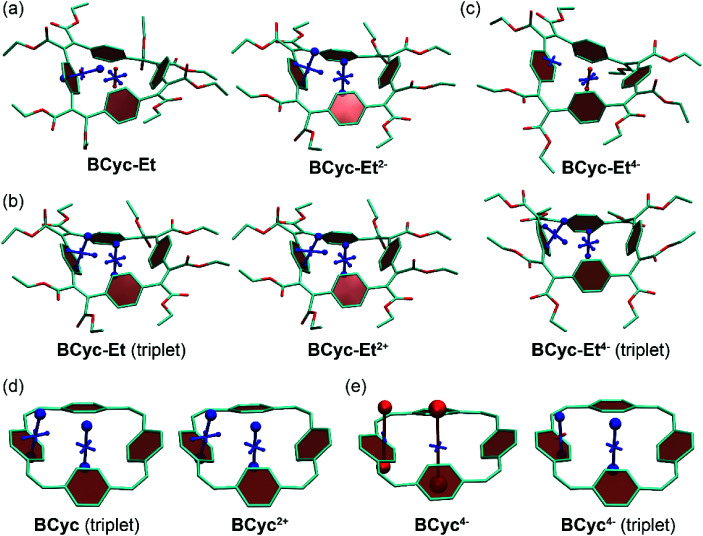
(a) to (c) VIST plots for **BCyc-Et** in different charge and spin states. (d) and (e) VIST plots for **BCyc** for comparison (see also Scheme 1b). Shielded (aromatic) tensor components are shown in blue, deshielded (antiaromatic) tensor components in red. Each tensor component relates to ring currents in a plane perpendicular to it. In all VIST plots, hydrogen atoms were omitted for clarity. Further VIST plots are provided in the ESI section 6.4.†

The systematic structural changes of the macrocycles as well as the changes in redox and spin state were also expected to lead to changes in magnetic properties (ring currents and chemical shielding) and the associated global (anti)aromaticity, providing a powerful framework for explaining the observed properties. To assess the local and global (anti)aromaticity, we carried out nucleus-independent chemical shift (NICS) calculations for the different states of the macrocycles. The NICS tensors were then represented graphically using the visualization of chemical shielding tensors (VIST) method (Fig. 5, further VIST plots are provided in Scheme 1b and in the ESI section 6.4†).^17^ VIST allows the visualization of local variations in aromaticity and antiaromaticity in the context of the molecular structure by showing the tensor components as blue (shielded, aromatic) or red (deshielded, antiaromatic) dumbbells. Each tensor component relates to ring currents in a plane perpendicular to it. VIST does not require the a-priori definition of an external magnetic field and, thus, provides an unbiased view of currents aligned in different planes, giving insight into the anisotropy of the chemical shielding, a highly valuable feature when studying multiring systems such as our macrocycles, where several ring currents in different planes can interact.

For each macrocycle and state, the NICS tensors were computed and visualized at two different positions: (i) 1 Å off the plane of a ring of the aromatic units, denoted NICS(1)_ar_ here, and (ii) at the center of the macrocycle, denoted NICS(0). The NICS(1)_ar_ tensor can tell us how strongly the local aromaticity of the aromatic units is perturbed by macrocyclic currents, while the NICS(0) tensor is best suited to provide information about the macrocyclic currents themselves. The VIST plots for **BCyc** and **BCyc2−** (Scheme 1b) illustrate the expected changes: in the neutral state, the main component of the NICS(1)_ar_ tensor is shielded and perpendicular to the plane of the aromatic unit. In the doubly reduced state, however, it is tilted and almost perpendicular to the plane of the macrocycle, indicating strong perturbation of the local aromaticity by macrocyclic currents, as described previously.^17^ In contrast, the main component of the central NICS(0) tensor is slightly deshielded (+11.0 ppm) in the neutral state but strongly shielded (−38.7 ppm) in the doubly reduced state. The deshielding in the neutral system may derive from paratropic currents in the macrocyclic [4*n*] π-electron system as well as from diatropic currents in the phenylene units (see also ref. 17 and 33), indicating either weakly antiaromatic or nonaromatic character, whereas the strong shielding in the dianion is an unequivocal sign of its aromaticity due to the resulting [4*n* + 2] π-electron system. A similar, but somewhat less pronounced, effect was observed in the VIST plots for **BCyc-Et** and **BCyc-Et2−** (Fig. 5a).

Before proceeding, we want to point out that the application of NICS in multiring systems has been challenged,^33^ as it is not generally possible to assign the contributions to the individual rings and more generally, following ref. 34, it should be clear that any method aimed at compressing the current density susceptibility tensor field to just a few numbers has to be applied carefully to retain all the chemically relevant information. For these reasons, we have performed additional computations of gauge-including magnetically induced currents (GIMIC)^25,35^ integrated across the plane bisecting a vinylene unit. As ESI Table S5† shows, GIMIC and NICS do not only show similar trends but provide even quantitative agreement for the strongly aromatic systems when the computed current is inserted into a conductor loop model. On the other hand, the GIMIC computations indicate only minuscule antiaromaticity for neutral **BCyc** and **BCyc-Et**, suggesting that the deshielding in these cases derives mostly from diatropic currents in the benzene units. In summary, this comparison highlights that VIST is suitable for a qualitative illustration of the overall magnetic properties and for a semi-quantitative discussion of the trends between different molecules whereas a fully quantitative picture would require a more involved analysis.

The VIST plots for **BCyc** and **BCyc-Et** in the triplet excited state as well as **BCyc2+** and **BCyc-Et2+** in the singlet ground state (Fig. 5d and b) showed similar NICS tensors as their dianions, indicating similarly strong aromatic macrocyclic currents (Baird aromaticity in the triplet excited state, Hückel aromaticity in the doubly charged ground states). Moving to the tetraanions, the VIST plots indicate strong antiaromatic macrocyclic currents in the singlet state (Hückel antiaromaticity) and strong aromatic currents in the triplet state of **BCyc4−** (Baird aromaticity) (Fig. 5e). The currents are considerably weaker in the corresponding states of **BCyc-Et4−** (Fig. 5c) and the reduction in current is particularly pronounced in the singlet state. Notwithstanding the weaker currents, the aromaticity in the triplet state and the antiaromaticity in the singlet state of the tetraanions provide an explanation for the unusually small difference in energy between the two states and the ensuing small difference in computed redox potentials (see Fig. 3).

To understand the weaker currents in the ester-substituted compound, we computed electron density difference plots (ESI section 6.5†). In these plots, **BCyc-Et** is shown to be able to place positive as well as negative charge on the ester groups, whereas for **BCyc** the changes in electron density are largely concentrated on the π-electron perimeter. As a consequence, **BCyc** acts more like an idealised [24]annulene in its charged states, switching between aromatic and antiaromatic states with the addition or subtraction of two electrons. This effect is alleviated for **BCyc-Et**, as other orbitals aside from the π-orbitals on the perimeter also play a significant role. The difference between the two macrocycles is particularly pronounced for the tetraanions. **BCyc4−** has no option aside from placing the additional electrons along the macrocyclic π-conjugated pathway, which induces strong antiaromaticity, as seen by the pronounced deshielding in the VIST plots as well as the strongly negative redox potential for the reduction to the tetraanion. By contrast, **BCyc-Et4−** is able to place the additional electron density into the ester groups, thus avoiding the formation of a strongly antiaromatic state, causing a dramatic shift in the computed redox potential for the reduction by about 1.5 V when compared to **BCyc4−**.

**Fig. 6:**
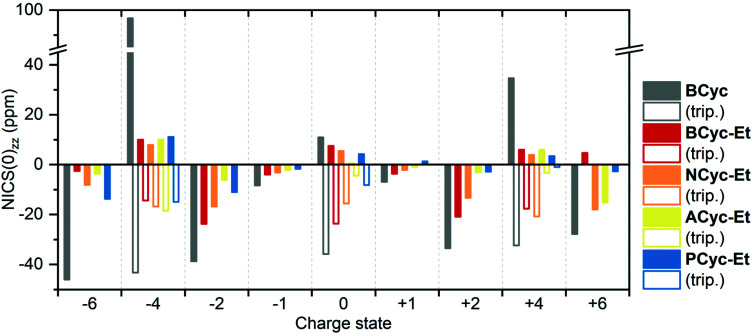
Comparison of NICS(0)_*zz*_ values of the macrocycles in different charge and spin states (singlet states: filled bars, triplet states: unfilled bars). Negative values indicate diatropic (aromatic) macrocyclic currents, positive values indicate paratropic (antiaromatic) macrocyclic currents. The corresponding data is available in the ESI Table S3.†

For comparing the full set of macrocycles in the different states, the component of the NICS(0) tensor perpendicular to the plane of the macrocycle, denoted NICS(0)_*zz*_, is most instructive, as it can serve as an indicator of the diatropic (aromatic) or paratropic (antiaromatic) macrocyclic currents (assuming that contributions from the currents in the aromatic units are weaker and cancel out in part). Shielded tensor components correspond to negative NICS(0)_*zz*_ values and indicate global aromaticity, deshielded tensor components correspond to positive values and indicate global antiaromaticity. Fig. 6 provides an overview of the NICS(0)_*zz*_ values of the macrocycles in the different states. Almost without exception, the signs of the NICS(0)_*zz*_ values agree with the predictions of (anti)aromaticity following Hückel's and Baird's rule, highlighting the consistency between these approaches. In all states, the values indicate a decrease of the macrocyclic currents with the introduction of the ester groups, as can be seen when comparing the values of **BCyc** and **BCyc-Et**, but the magnitude of the decrease depends on the state. In the neutral singlet state, the NICS(0)_*zz*_ values indicate only a small decrease (by about one third) in paratropic currents upon introduction of the ester groups, whereas the decrease is somewhat larger for the mono- and dianions. A further stepwise decrease can be observed when increasing the size of the aromatic units, moving to **NCyc-Et** and **PCyc-Et**. Only **ACyc-Et** deviates from this general trend, a result of its folded conformation. In the neutral triplet state, the NICS(0)_*zz*_ values indicate macrocyclic currents that are significantly stronger than the currents in the neutral singlet state, suggesting that the excited-state Baird aromaticity is more pronounced than the ground-state Hückel antiaromaticity in the neutral macrocycles.

To interpret the data from Fig. 6 in some more detail, it is important to note that the strength of the ring current is approximately proportional to the number of electrons contributing^36^ and, accordingly, the diamagnetic shielding is proportional to the number of electrons divided by the radius of the ring.^37^ In line with this, we find that due to the enhanced number of electrons the shielding for the anionic systems is slightly more negative than for the cationic systems. This is particularly true when comparing the dianions with the dications, a trend that is also seen for smaller annulenes.^38^ Looking at the radii, we find that going from **BCyc-Et** to **PCyc-Et** the radius increases by about 50% (from 4 Å to 6 Å). All else equal, one would expect the NICS(0)_*zz*_ values to decrease by a third. However, the NICS(0)_*zz*_ values of the dianion and dication decrease by more than half (despite the increased number of electrons), highlighting that the aromaticity of these states in **PCyc-Et** is clearly reduced when compared to **BCyc-Et**.

**Fig. 7:**
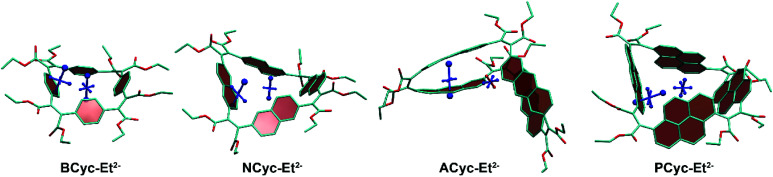
VIST plots for the dianions of the macrocycles, highlighting that global aromaticity dominates for **BCyc-Et2−** and **NCyc-Et2−** whereas local aromaticity remains for **ACyc-Et2−** and **PCyc-Et2−**. Hydrogen atoms omitted for clarity.

To understand the reduced global aromaticity for the macrocycles containing larger aromatic systems, it is instructive to view their VIST plots, as exemplified in Fig. 7 for the dianions. **BCyc-Et2−**, shown on the left, exhibits the expected behavior for a globally aromatic system, as the dominant contributions for both, the NICS(0) and NICS(1)_ar_ tensors, are approximately perpendicular to the plane of the macrocycle. A similar behavior but slightly less pronounced is found for **NCyc-Et2−**. The two larger macrocycles, **ACyc-Et2−** and **PCyc-Et2−**, show an entirely different behavior; the NICS(1)_ar_ tensor stands perpendicular to the aromatic unit and the NICS(0) tensor almost vanishes. This discussion supports the interpretation that the dianions of the smaller molecules become globally aromatic at the expense of local aromaticity of the individual units, whereas the local aromaticity is maintained at the cost of weaker global aromaticity for the larger macrocycles.

### Stokes shifts

As shown in Table 1, all the molecules studied possess strong Stokes shifts of more than 1 eV and this effect is particularly pronounced for **BCyc-Et**. In this section, we consider excited-state energies and shielding tensors to examine if these Stokes shifts can be seen as a consequence of excited-state aromaticity (see also ref. 39). For this discussion, one would ideally compute shielding tensors for the S_1_ state at the relaxed S_1_ geometry. However, neither a geometry optimisation nor a computation of shielding tensors in the S_1_ state is routinely possible for molecules of the size considered here. A TDDFT optimisation of S_1_ is not only computationally demanding but is also sensitive to spurious charge transfer problems^40,41^ in systems of the size considered here. Therefore, we optimised the triplet geometry instead, using unrestricted Kohn–Sham theory (UKS) as a computationally efficient and numerically stable alternative. At the UKS level, it is also straightforward to compute the shielding tensors relevant to the T_1_ state. For the following analysis, it is worth noting that S_1_ and T_1_ are both dominated by the HOMO/LUMO transition and, thus, we expect that any statement made about the T_1_ in terms of molecular geometry and electronic structure is also transferable to the S_1_ (see ref. 37 and 42–44 for more information on the similarities of excited-state aromaticity in singlet and triplet excited states).

**Fig. 8:**
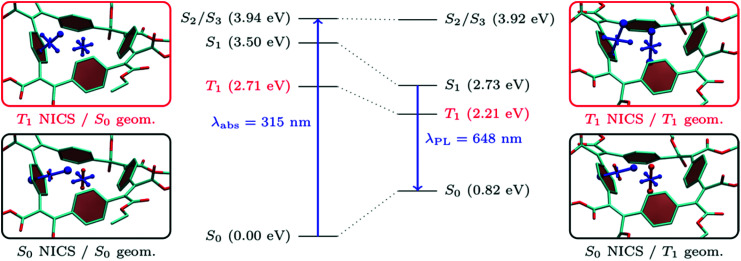
Comparison of energetic and magnetic properties of the electronic states of **BCyc-Et** optimized for the lowest singlet (S_0_) and triplet (T_1_). Energies relative to the S_0_ minimum and the associated absorption (*λ*_abs_) and photoluminescence (*λ*_PL_) wavelengths are shown in the center. VIST plots for the S_0_ and T_1_ states, each for the S_0_ and T_1_ geometries, are shown on the sides.

In the centre of Fig. 8, we present the total energies of the S_0_, T_1_, S_1_, S_2_, and S_3_ states computed at the S_0_ and T_1_ geometries. As discussed above, the transition to the S_1_ state is symmetry-forbidden and the first band in the absorption spectrum relates to the degenerate S_2_/S_3_ states. These are located at 3.94 eV at the S_0_ geometry, which corresponds to a computed absorption wavelength (*λ*_abs_) of 315 nm. The photoluminescence wavelength (*λ*_PL_) is approximated here *via* the energy difference of S_1_ and S_0_ at the T_1_ geometry, obtaining a value of 1.91 eV or 648 nm. The computed photoluminescence energy is about 0.4 eV lower than the experimental value but, importantly, reflects the large Stokes shift and we are confident that the following discussion based on T_1_ correctly captures the underlying physics related to the strong Stokes shifts observed. Fig. 8 highlights that the large Stokes shift is obtained *via* a combination of three effects: (i) internal conversion from S_2_/S_3_ to S_1_ according to Kasha's rule, (ii) destabilization of S_0_, and (iii) stabilization of S_1_ as the excited-state geometry is relaxed. The first effect amounts to about 0.4 eV while the latter two contribute about 0.8 eV each. It should be noted that emission from the S_1_ state is also symmetry forbidden at the excited-state geometry, highlighting that only rather weak luminescence is expected.

Interestingly, the total energy of the S_2_/S_3_ states is almost unaffected by the structure optimization. Within the MO picture, this difference can be explained by the fact that the structural relaxation strongly stabilizes the LUMO (from −2.4 to −4.8 eV at the PBE0/def2-SV(P) level), leaving the other orbitals largely unaffected. Therefore, only the T_1_ and S_1_, which are dominated by the HOMO/LUMO transition, are notably affected.

Moving beyond the MO picture, we can endeavor to explain the effects within the framework of excited-state aromaticity. Specifically, we propose that the geometry relaxation in the excited state produces enhanced excited-state aromaticity for T_1_/S_1_ in connection with enhanced ground-state antiaromaticity for S_0_. To examine this hypothesis, we have computed chemical shielding tensors related to the S_0_ and T_1_ states at the S_0_ (Fig. 8, left) and T_1_ (Fig. 8, right) geometries. Starting with the chemical shielding of the S_0_ state computed at the S_0_ geometry, shown on the lower left in Fig. 8, we find a slightly deshielded out-of-plane component for the NICS(0) tensor (NICS(0)_*zz*_, 7.5 ppm). The NICS(1)_ar_ tensor, on the other hand, shows one strongly shielded component perpendicular to the benzene unit, indicating its local aromaticity. The formal vertical excitation to T_1_, represented in the upper left panel, induces only modest Baird aromaticity and the NICS(0)_*zz*_ component only becomes slightly shielded (−6.3 ppm) along with minor variations of NICS(1)_ar_. The situation changes dramatically once the triplet geometry is allowed to relax, as seen on the upper right in Fig. 8. For the relaxed T_1_ state one finds strong indications of global aromaticity for both the NICS(0) and NICS(1)_ar_ positions (−23.6 and −29.2 ppm). Staying at the T_1_ geometry but performing computations for the S_0_ state, we find notably enhanced antiaromaticity when compared to the S_0_ geometry, yielding a dominant NICS(0) component of 14.9 ppm. However, the dominant component of NICS(1)_ar_ (−20.4 ppm) keeps pointing away from the phenylene ring, highlighting that much of its local aromaticity is restored.

From the viewpoint of molecular structures, it is observed that the bond length alternation changes significantly when going from the ground- to excited-state optimized structure. In the ground state, there is a clear difference between the length of the vinylene double bond (1.35 Å) and that of the single bond connecting vinylene and phenylene (1.48 Å), whereas this difference is significantly reduced at the T_1_ geometry (1.39/1.44 Å). The reduction in bond-length alternation is similar to what is observed in the open-chain analogue, poly(*p*-phenylene vinylene) (PPV).^45^ However, the consequences are much more pronounced in the cyclic system presented here, noting that the stabilization of S_1_ upon geometry relaxation in open-chain PPV amounts to only about 0.2 eV,^45^ as opposed to the 0.8 eV found here.

## Conclusions

The results of this detailed experimental and computational study of a set of [2.2.2.2]cyclophanetetraenes show that systematic variations of the molecular structure can indeed produce pronounced variations in terms of their redox- and photochemistry. The study advances our fundamental understanding of such systems, helping to develop structure–property relationships and molecular design guidelines for π-conjugated macrocycles, paving the way to the development of macrocycles for new applications and assisting in the design of macrocycle-based supramolecular elements. The structural variations investigated, the introduction of electron-withdrawing ethyl or hexyl ester groups and the variation of the aromatic units, yielded a set of six ester-substituted π-conjugated macrocycles and were found to have drastic effects on some of the properties but also on the synthesis. The performed structural variations may also be used to control the non-covalent interactions when designing macrocycle-based supramolecular elements; the ester groups may serve as hydrogen bond acceptors or in dipole–dipole interactions while the larger aromatic units can strengthen π–π interactions or cation–π interactions (either inside or outside the molecular cavity).

While the synthesis of some cyclization precursors required considerable optimization, the synthesis of the macrocycles themselves was generally found to be straightforward when adapting previously reported conditions, except for the synthesis of the ester-substituted macrocycle with anthracene units, **ACyc-Et**, which suffered from poor reproducibility. In contrast to the other macrocycles, the computations indicated a folded conformation for **ACyc-Et**, leading us to conclude that small structural variations can result in significant differences in conformation that may affect the synthesis. The folded conformation also resulted in a markedly different shape of the absorption spectrum due to the entailing loss of symmetry.

We can further conclude that the introduction of hexyl instead of ethyl ester groups can give π-conjugated macrocycles that are viscous oils. Even with large pyrene units, these macrocycles did not crystallize during prolonged storage and dissolved well in organic solvents. The hexyl ester-substituted macrocycles also showed a significantly more intense photoluminescence than their ethyl ester analogs, which we attributed to enhanced shielding of the chromophores by the larger hexyl ester groups, enabling emission from the formally symmetry-forbidden lowest singlet excited state. In contrast to the photoluminescence spectra, the absorption spectra of the ethyl and hexyl ester analogs were almost identical.

Regarding the electrochemical properties, the introduction of the ester groups was found to drastically shift the first reduction wave to higher potential in the cyclic voltammetry measurements (compared to the previously reported unsubstituted macrocycle [2.2.2.2]paracyclophane-1,9,17,25-tetraene, denoted **BCyc** in this work). Computations showed that even more pronounced shifts occur for the reduction to the tetra- and hexaanions. In contrast, increasing the size of the aromatic units was found to have its most pronounced effect on the oxidation to the tetra- and hexacations. From EPR spectroelectrochemical measurements, we conclude that the two reversible reduction steps observed in the cyclic voltammogram of **BCyc-Et** are two consecutive two-electron processes. This reversible fourfold reduction, in combination with the commercial availability of one of the cyclisation precursors and the lower molecular weight compared to the other macrocycles, renders **BCyc-Et** the most interesting macrocycle to be used, for example, as a building block for battery electrode materials or as component of a multielectron photoredox catalyst.

A detailed investigation of the ring currents and chemical shielding allowed us to rationalize many of the observed optoelectronic properties in the context of local and global aromaticity in different planes. The recently developed method for the visualization of chemical shielding tensors (VIST) was found to provide particularly useful insights in this regard. Most importantly, the method provided an intuitive explanation of the large Stokes shifts of up to 1.6 eV, showing that emission occurs at a geometry where excited-state aromaticity and ground-state antiaromaticity are both significantly increased, thus, narrowing the gap between the states. A more detailed analysis of excited-state energies was presented to substantiate this viewpoint.

From the weaker global aromaticity and antiaromaticity in the different charge and spin states after introduction of the ester groups, it can be concluded that the ester-substituted macrocycles have additional flexibility of placing positive as well as negative charge on the ester groups, largely explaining the observed redox properties; the effect was further confirmed by electron density difference plots. It can further be concluded that, as a general trend, increasing the size of the aromatic units results in weaker global aromaticity but stronger local aromaticity.

In summary, we have shown that [2.2.2.2]cyclophanetetraenes provide versatile scaffolds for applications as molecular materials or supramolecular elements due to their unusual properties along with their high tunability. Their remarkable optoelectronic properties, in particular their large Stokes shifts and the accessibility of a variety of charged states, were traced back to their formal ground state antiaromaticity – illustrated *via* VIST plots – along with high structural flexibility. We have shown that their properties can be effectively tuned *via* two independent strategies: (i) introduction of electron-withdrawing groups to allow for easier reduction and (ii) variation of the aromatic groups to alter the balance between local and global aromaticity, indicating that a wide range of target properties can be achieved using this remarkable class of molecules as basis for molecular design.

## Methods

Experimental details for the synthesis and purification of all compounds as well as characterization results confirming their identity and purity are provided in the ESI (see section 1.3 for compounds **1a–e**, section 1.4 for precursors **2a–e**, section 1.5 for precursors **3b–d**, and section 1.6 for macrocycles **BCyc-Et** to **PCyc-Hx**†).

UV-vis absorption spectra were recorded on an Agilent Cary 60 UV-vis spectrophotometer at room temperature. The measurements were carried out with 5 μM solutions in CHCl_3_ at a scan rate of 300 nm min^−1^ and a data interval of 0.5 nm. Photoluminescence (PL) spectra were acquired on an Agilent Cary Eclipse fluorescence spectrophotometer with 5 μM solutions in CHCl_3_ at a scan rate of 120 nm min^−1^ and a data interval of 1 nm. The excitation and emission slits were set to 5 nm, the emission and excitation filters were set to ‘auto’ setting, and the detector voltage was set to ‘high’ (800 V). For **PCyc-Et** and **PCyc-Hx** additional spectra were recorded using ‘medium’ (600 V) detector voltage (ESI Fig. S44†), due to the peak of **PCyc-Hx** exceeding the maximum of 1000 counts with the ‘high’ setting.

Cyclic voltammetry measurements were performed in an argon atmosphere glovebox (LabStar, MBraun) using a PalmSens4 potentiostat controlled *via* Bluetooth connection in a standard three-electrode setup. Platinum disk electrodes of 2 mm diameter, a silver wire, and a platinum wire served as working, quasi-reference, and auxiliary electrodes, respectively. Small glass test tube vessels were used as open electrochemical cells. The electrolyte volume was below 0.4 mL. After the measurements, an arbitrary amount of ferrocene (internal reference) was added to the solution to evaluate the redox potentials of the studied compounds (ESI Fig. S45 and S48†). Cyclic voltammograms were fitted to simulated voltammograms using the DigiSim 3.03b software (Bioanalytical Systems). For steady-state voltammetry (ESI Fig. S47†), platinum disk ultramicroelectrodes of 25 μm diameter were used as the working electrode. Details for the EPR spectroelectrochemical measurements are provided in the ESI section 5.†

Density functional theory computations considered singlet states of charge −6, −4, −2, −1, 0, +1, +2, +4, +6 as well as triplet states of charge −4, 0, +4. Geometries for these electronic states were optimized in vacuum using the PBE0 functional^46,47^ along with the def2-SV(P) basis set^48^ and the D3 dispersion correction^49^ in its optimized power parameterization.^50^ The nature of the stationary points as minima was verified using a finite difference Davidson procedure^51^ to avoid computation of the full *ab initio* Hessian.

Redox potentials were determined *via* single-point computations on the gas-phase optimized structures for the individual states using PBE0-D3 along with the def2-SVPD basis set (possessing additional diffuse basis functions) and including solvation effects using a conductor-like polarizable continuum model^52^ considering a dielectric constant *ε* of 10.125 to represent 1,2-dichloroethane. First, the ionization potential (IP) of a state of molecular charge (*z*) was computed according toIP(*z*) = *G*(*z*) − G(0)

where *G*(*z*) corresponds to the total free energy in solution at the PBE0-D3/def2-SVPD level. No vibrational effects were included considering that a vibrational analysis was unfeasible for the largest systems considered and noting that vibrational effects are generally expected to play a minor role for redox potentials.^53^ Subsequently the redox potential for any given redox couple *z*_1_/*z*_2_ was computed as
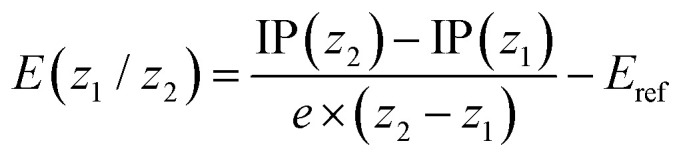
where *e* is the unit charge and *E*_ref_ is the absolute potential of the reference electrode (*cf.* ref. 53). A value of 4.70 V was used for *E*_ref_. Redox potentials for the reductions were computed in complete analogy, only using the electron affinity instead of the IP. The values reported pertain to the redox couples (−6/−4), (−4/−2), (−2/−1), (−1/0), (0/+1), (+1/+2), (+2/+4), (+4/+6). These calculations were carried out in Q-Chem.^54,55^

Absorption wavelengths were computed using time-dependent DFT (TDDFT) with the ωPBEh functional^46,56^ (using 20% global Hartree–Fock exchange and *ω* = 0.1 a.u.) and the def2-SV(P) basis set and considering solvation in 1,2-dichloroethane (*ε* = 10.125, *ε*_∞_ = 2.087). Approximate photoluminescence energies were computed at the triplet geometries, optimized *via* unrestricted Kohn–Sham (UKS) theory, considering that TDDFT geometry optimizations are not feasible for the largest systems considered here.

Nucleus independent chemical shifts (NICS)^28^ were computed at the PBE0/def2-SVP level using gauge including atomic orbitals^57^ as implemented in Gaussian 09.^58^ NICS tensors were represented graphically using the VIST (visualization of chemical shielding tensors) method^17^ as implemented in TheoDORE 2.4^59^ and using VMD for rendering the figures.^60^ Additional current density computations were performed using the GIMIC 2.1.4 package^25,35^ in connection with PBE0/def2-SVP chemical shift computations in Turbomole 7.4.^61,62^ These current densities were integrated along a plane bisecting the C=C double bond in a vinylene unit. Plots of the anisotropy of the induced current density (ACID) were computed using the AICD 3.0.3 program^23^ in connection with PBE0/def2-SVP computations in Gaussian 09.

The underlying computational research data is available *via* a separate repository (DOI: 10.17028/rd.lboro.14500482): geometries for the molecules studied in their different electronic states along with input/output files for characterization of stationary points, solvated calculations, vertical excitations, and NICS.

## Conflicts of interest

There are no conflicts to declare.

## Supplementary Material

QO-008-D1QO00901J-s001

## References

[cit1] Iyoda M., Yamakawa J., Rahman M. J. (2011). Conjugated Macrocycles: Concepts and Applications. Angew. Chem., Int. Ed..

[cit2] Ball M., Zhong Y., Fowler B., Zhang B., Li P., Etkin G., Paley D. W., Decatur J., Dalsania A. K., Li H., Xiao S., Ng F., Steigerwald M. L., Nuckolls C. (2016). Macrocyclization in the Design of Organic n-Type Electronic Materials. J. Am. Chem. Soc..

[cit3] Zhang B., Trinh M. T., Fowler B., Ball M., Xu Q., Ng F., Steigerwald M. L., Zhu X. Y., Nuckolls C., Zhong Y. (2016). Rigid, Conjugated Macrocycles for High Performance Organic Photodetectors. J. Am. Chem. Soc..

[cit4] Zhang B., Hernández Sánchez R., Zhong Y., Ball M., Terban M. W., Paley D., Billinge S. J. L., Ng F., Steigerwald M. L., Nuckolls C. (2018). Hollow organic capsules assemble into cellular semiconductors. Nat. Commun..

[cit5] Ball M. L., Zhang B., Xu Q., Paley D. W., Ritter V. C., Ng F., Steigerwald M. L., Nuckolls C. (2018). Influence of Molecular Conformation on Electron Transport in Giant, Conjugated Macrocycles. J. Am. Chem. Soc..

[cit6] Singh K., Sharma A., Zhang J., Xu W., Zhu D. (2011). New sulfur bridged neutral annulenes. Structure, physical properties and applications in organic field-effect transistors. Chem. Commun..

[cit7] Xu Q., Zhang B., Zeng Y., Zangiabadi A., Ni H., Chen R., Ng F., Steigerwald M. L., Nuckolls C. (2021). Electrical conductivity in a non-covalent two-dimensional porous organic material with high crystallinity. Chem. Sci..

[cit8] Izumi S., Higginbotham H. F., Nyga A., Stachelek P., Tohnai N., Silva P. d., Data P., Takeda Y., Minakata S. (2020). Thermally Activated Delayed Fluorescent Donor–Acceptor–Donor–Acceptor π-Conjugated Macrocycle for Organic Light-Emitting Diodes. J. Am. Chem. Soc..

[cit9] Xue J. Y., Izumi T., Yoshii A., Ikemoto K., Koretsune T., Akashi R., Arita R., Taka H., Kita H., Sato S., Isobe H. (2016). Aromatic hydrocarbon macrocycles for highly efficient organic light-emitting devices with single-layer architectures. Chem. Sci..

[cit10] Izumi T., Tian Y., Ikemoto K., Yoshii A., Koretsune T., Arita R., Kita H., Taka H., Sato S., Isobe H. (2017). Efficient Blue Electroluminescence from a Single-layer Organic Device Composed Solely of Hydrocarbons. Chem. – Asian J..

[cit11] Shikita S., Watanabe G., Kanouchi D., Saito J., Yasuda T. (2021). Alternating Donor–Acceptor π-Conjugated Macrocycle Exhibiting Efficient Thermally Activated Delayed Fluorescence and Spontaneous Horizontal Molecular Orientation. Adv. Photonics Res..

[cit12] White B. M., Zhao Y., Kawashima T. E., Branchaud B. P., Pluth M. D., Jasti R. (2018). Expanding the Chemical Space of Biocompatible Fluorophores: Nanohoops in Cells. ACS Cent. Sci..

[cit13] Omachi H., Nakayama T., Takahashi E., Segawa Y., Itami K. (2013). Initiation of carbon nanotube growth by well-defined carbon nanorings. Nat. Chem..

[cit14] Fontana L. A., Almeida M. P., Alcântara A. F. P., Rigolin V. H., Ribeiro M. A., Barros W. P., Megiatto J. D. (2020). Ru(II)Porphyrinate-based molecular nanoreactor for carbene insertion reactions and quantitative formation of rotaxanes by active-metal-template syntheses. Nat. Commun..

[cit15] Schmidt H.-W., Würthner F. (2020). A Periodic System of Supramolecular Elements. Angew. Chem., Int. Ed..

[cit16] Eder S., Yoo D.-J., Nogala W., Pletzer M., Santana Bonilla A., White A. J. P., Jelfs K. E., Heeney M., Choi J. W., Glöcklhofer F. (2020). Switching between Local and Global Aromaticity in a Conjugated Macrocycle for High-Performance Organic Sodium-Ion Battery Anodes. Angew. Chem., Int. Ed..

[cit17] Plasser F., Glöcklhofer F. (2021). Visualisation of chemical shielding tensors (VIST) to elucidate aromaticity and antiaromaticity. Eur. J. Org. Chem..

[cit18] Lewis S. E. (2015). Cycloparaphenylenes and related nanohoops. Chem. Soc. Rev..

[cit19] Leonhardt E. J., Jasti R. (2019). Emerging applications of carbon nanohoops. Nat. Rev. Chem..

[cit20] Krygowski T. M., Cyrański M. K. (2001). Structural Aspects of Aromaticity. Chem. Rev..

[cit21] Matito E., Duran M., Solà M. (2004). The aromatic fluctuation index (FLU): A new aromaticity index based on electron delocalization. J. Chem. Phys..

[cit22] Steiner E., Fowler P. W. (2001). Patterns of Ring Currents in Conjugated Molecules: A Few-Electron Model Based on Orbital Contributions. J. Phys. Chem. A.

[cit23] Geuenich D., Hess K., Köhler F., Herges R. (2005). Anisotropy of the Induced Current Density (ACID), a General Method To Quantify and Visualize Electronic Delocalization. Chem. Rev..

[cit24] Chen Z., Wannere C. S., Corminboeuf C., Puchta R., Schleyer P. v. R. (2005). Nucleus-Independent Chemical Shifts (NICS) as an Aromaticity Criterion. Chem. Rev..

[cit25] Fliegl H., Taubert S., Lehtonen O., Sundholm D. (2011). The gauge including magnetically induced current method. Phys. Chem. Chem. Phys..

[cit26] Foroutan-Nejad C., Ghosh A. (2018). Magnetic Diversity in Heteroisocorroles: Aromatic Pathways in 10-Heteroatom-Substituted Isocorroles. ACS Omega.

[cit27] Stanger A., Monaco G., Zanasi R. (2020). NICS-XY-Scan Predictions of Local, Semi-Global, and Global Ring Currents in Annulated Pentalene and s-Indacene Cores Compared to First-Principles Current Density Maps. ChemPhysChem.

[cit28] Schleyer P. v. R., Maerker C., Dransfeld A., Jiao H., van Eikema Hommes N. J. R. (1996). Nucleus-Independent Chemical Shifts: A Simple and Efficient Aromaticity Probe. J. Am. Chem. Soc..

[cit29] Robert A., Dechambenoit P., Bock H., Durola F. (2017). A carboxyfunctionalized (24)-1,6-pyrenophane-tetraene by glyoxylic Perkin condensation. Can. J. Chem..

[cit30] Robert A., Dechambenoit P., Hillard E. A., Bock H., Durola F. (2017). Non-planar oligoarylene macrocycles from biphenyl. Chem. Commun..

[cit31] Zhuang J., Wang C., Xie F., Zhang W. (2009). One-pot efficient synthesis of aryl α-keto esters from aryl-ketones. Tetrahedron.

[cit32] Naulet G., Robert A., Dechambenoit P., Bock H., Durola F. (2018). Monoprotection of Arylene-Diacetic Acids Allowing the Build-Up of Longer Aromatic Ribbons by Successive Perkin Condensations. Eur. J. Org. Chem..

[cit33] Stanger A. (2020). NICS – Past and Present. Eur. J. Org. Chem..

[cit34] Van Damme S., Acke G., Havenith R. W. A., Bultinck P. (2016). Can the current density map topology be extracted from the nucleus independent chemical shifts?. Phys. Chem. Chem. Phys..

[cit35] Fliegl H., Jusélius J., Sundholm D. (2016). Gauge-Origin Independent Calculations of the Anisotropy of the Magnetically Induced Current Densities. J. Phys. Chem. A.

[cit36] Foroutan-Nejad C., Shahbazian S., Feixas F., Rashidi-Ranjbar P., Solà M. (2011). A dissected ring current model for assessing magnetic aromaticity: A general approach for both organic and inorganic rings. J. Comput. Chem..

[cit37] Plasser F. (2021). Exploitation of Baird Aromaticity and Clar's Rule for Tuning the Triplet Energies of Polycyclic Aromatic Hydrocarbons. Chemistry.

[cit38] Corminboeuf C., Heine T., Seifert G., Schleyer P. v. R., Weber J. (2004). Induced magnetic fields in aromatic [n]-annulenes—interpretation of NICS tensor components. Phys. Chem. Chem. Phys..

[cit39] Kotani R., Liu L., Kumar P., Kuramochi H., Tahara T., Liu P., Osuka A., Karadakov P. B., Saito S. (2020). Controlling the S1 Energy Profile by Tuning Excited-State Aromaticity. J. Am. Chem. Soc..

[cit40] Dreuw A., Weisman J. L., Head-Gordon M. (2003). Long-range charge-transfer excited states in time-dependent density functional theory require non-local exchange. J. Chem. Phys..

[cit41] Mewes S. A., Plasser F., Dreuw A. (2017). Universal Exciton Size in Organic Polymers is Determined by Nonlocal Orbital Exchange in Time-Dependent Density Functional Theory. J. Phys. Chem. Lett..

[cit42] Karadakov P. B. (2008). Ground- and Excited-State Aromaticity and Antiaromaticity in Benzene and Cyclobutadiene. J. Phys. Chem. A.

[cit43] Rosenberg M., Dahlstrand C., Kilså K., Ottosson H. (2014). Excited State Aromaticity and Antiaromaticity: Opportunities for Photophysical and Photochemical Rationalizations. Chem. Rev..

[cit44] El Bakouri O., Smith J. R., Ottosson H. (2020). Strategies for Design of Potential Singlet Fission Chromophores Utilizing a Combination of Ground-State and Excited-State Aromaticity Rules. J. Am. Chem. Soc..

[cit45] Panda A. N., Plasser F., Aquino A. J. A., Burghardt I., Lischka H. (2013). Electronically Excited States in Poly(p-phenylenevinylene): Vertical Excitations and Torsional Potentials from High-Level Ab Initio Calculations. J. Phys. Chem. A.

[cit46] Perdew J. P., Burke K., Ernzerhof M. (1996). Generalized Gradient Approximation Made Simple. Phys. Rev. Lett..

[cit47] Adamo C., Barone V. (1999). Toward reliable density functional methods without adjustable parameters: The PBE0 model. J. Chem. Phys..

[cit48] Weigend F., Ahlrichs R. (2005). Balanced basis sets of split valence, triple zeta valence and quadruple zeta valence quality for H to Rn: Design and assessment of accuracy. Phys. Chem. Chem. Phys..

[cit49] Grimme S., Antony J., Ehrlich S., Krieg H. (2010). A consistent and accurate ab initio parametrization of density functional dispersion correction (DFT-D) for the 94 elements H-Pu. J. Chem. Phys..

[cit50] Witte J., Mardirossian N., Neaton J. B., Head-Gordon M. (2017). Assessing DFT-D3 Damping Functions Across Widely Used Density Functionals: Can We Do Better?. J. Chem. Theory Comput..

[cit51] Sharada S. M., Bell A. T., Head-Gordon M. (2014). A finite difference Davidson procedure to sidestep full ab initio hessian calculation: Application to characterization of stationary points and transition state searches. J. Chem. Phys..

[cit52] Barone V., Cossi M. (1998). Quantum Calculation of Molecular Energies and Energy Gradients in Solution by a Conductor Solvent Model. J. Phys. Chem. A.

[cit53] Baik M.-H., Friesner R. A. (2002). Computing Redox Potentials in Solution: Density Functional Theory as A Tool for Rational Design of Redox Agents. J. Phys. Chem. A.

[cit54] Shao Y., Gan Z., Epifanovsky E., Gilbert A. T. B., Wormit M., Kussmann J., Lange A. W., Behn A., Deng J., Feng X., Ghosh D., Goldey M., Horn P. R., Jacobson L. D., Kaliman I., Khaliullin R. Z., Kuś T., Landau A., Liu J., Proynov E. I., Rhee Y. M., Richard R. M., Rohrdanz M. A., Steele R. P., Sundstrom E. J., Woodcock H. L., Zimmerman P. M., Zuev D., Albrecht B., Alguire E., Austin B., Beran G. J. O., Bernard Y. A., Berquist E., Brandhorst K., Bravaya K. B., Brown S. T., Casanova D., Chang C.-M., Chen Y., Chien S. H., Closser K. D., Crittenden D. L., Diedenhofen M., DiStasio R. A., Do H., Dutoi A. D., Edgar R. G., Fatehi S., Fusti-Molnar L., Ghysels A., Golubeva-Zadorozhnaya A., Gomes J., Hanson-Heine M. W. D., Harbach P. H. P., Hauser A. W., Hohenstein E. G., Holden Z. C., Jagau T.-C., Ji H., Kaduk B., Khistyaev K., Kim J., Kim J., King R. A., Klunzinger P., Kosenkov D., Kowalczyk T., Krauter C. M., Lao K. U., Laurent A. D., Lawler K. V., Levchenko S. V., Lin C. Y., Liu F., Livshits E., Lochan R. C., Luenser A., Manohar P., Manzer S. F., Mao S.-P., Mardirossian N., Marenich A. V., Maurer S. A., Mayhall N. J., Neuscamman E., Oana C. M., Olivares-Amaya R., O'Neill D. P., Parkhill J. A., Perrine T. M., Peverati R., Prociuk A., Rehn D. R., Rosta E., Russ N. J., Sharada S. M., Sharma S., Small D. W., Sodt A., Stein T., Stück D., Su Y.-C., Thom A. J. W., Tsuchimochi T., Vanovschi V., Vogt L., Vydrov O., Wang T., Watson M. A., Wenzel J., White A., Williams C. F., Yang J., Yeganeh S., Yost S. R., You Z.-Q., Zhang I. Y., Zhang X., Zhao Y., Brooks B. R., Chan G. K. L., Chipman D. M., Cramer C. J., Goddard W. A., Gordon M. S., Hehre W. J., Klamt A., Schaefer H. F., Schmidt M. W., Sherrill C. D., Truhlar D. G., Warshel A., Xu X., Aspuru-Guzik A., Baer R., Bell A. T., Besley N. A., Chai J.-D., Dreuw A., Dunietz B. D., Furlani T. R., Gwaltney S. R., Hsu C.-P., Jung Y., Kong J., Lambrecht D. S., Liang W., Ochsenfeld C., Rassolov V. A., Slipchenko L. V., Subotnik J. E., Van Voorhis T., Herbert J. M., Krylov A. I., Gill P. M. W., Head-Gordon M. (2015). Advances in molecular quantum chemistry contained in the Q-Chem 4 program package. Mol. Phys..

[cit55] Krylov A. I., Gill P. M. W. (2013). Q-Chem: an engine for innovation. Wiley Interdiscip. Rev.: Comput. Mol. Sci..

[cit56] Rohrdanz M. A., Martins K. M., Herbert J. M. (2009). A long-range-corrected density functional that performs well for both ground-state properties and time-dependent density functional theory excitation energies, including charge-transfer excited states. J. Chem. Phys..

[cit57] Cheeseman J. R., Trucks G. W., Keith T. A., Frisch M. J. (1996). A comparison of models for calculating nuclear magnetic resonance shielding tensors. J. Chem. Phys..

[cit58] Frisch M. J., Trucks G. W., Schlegel H. B., Scuseria G. E., Robb M. A., Cheeseman J. R., Scalmani G., Barone V., Mennucci B., Petersson G. A., Nakatsuji H., Caricato M., Li X., Hratchian H. P., Izmaylov A. F., Bloino J., Zheng G., Sonnenberg J. L., Hada M., Ehara M., Toyota K., Fukuda R., Hasegawa J., Ishida M., Nakajima T., Honda Y., Kitao O., Nakai H., Vreven T., Montgomery Jr. J. A., Peralta J. E., Ogliaro F., Bearpark M., Heyd J. J., Brothers E., Kudin K. N., Staroverov V. N., Keith T., Kobayashi R., Normand J., Raghavachari K., Rendell A., Burant J. C., Iyengar S. S., Tomasi J., Cossi M., Rega N., Millam J. M., Klene M., Knox J. E., Cross J. B., Bakken V., Adamo C., Jaramillo J., Gomperts R., Stratmann R. E., Yazyev O., Austin A. J., Cammi R., Pomelli C., Ochterski J. W., Martin R. L., Morokuma K., Zakrzewski V. G., Voth G. A., Salvador P., Dannenberg J. J., Dapprich S., Daniels A. D., Farkas O., Foresman J. B., Ortiz J. V., Cioslowski J. and Fox D. J., Gaussian 09, Revision E.01, Wallingford, CT, 2013

[cit59] Plasser F. (2020). TheoDORE: A toolbox for a detailed and automated analysis of electronic excited state computations. J. Chem. Phys..

[cit60] Humphrey W., Dalke A., Schulten K. (1996). VMD: Visual molecular dynamics. J. Mol. Graphics.

[cit61] TURBOMOLE V7.4 2019, a development of University of Karlsruhe and Forschungszentrum Karlsruhe GmbH, 1989–2007, TURBOMOLE GmbH, since 2007; available from http://www.turbomole.com

[cit62] Häser M., Ahlrichs R., Baron H. P., Weis P., Horn H. (1992). Direct computation of second-order SCF properties of large molecules on workstation computers with an application to large carbon clusters. Theor. Chim. Acta.

