# Supplementary material for: Functional group introduction and aromatic unit variation in a set of π-conjugated macrocycles: revealing the central role of local and global aromaticity
Source: Org Chem Front. 2021 Jun 22;8(17):4730–45. doi: 10.1039/d1qo00901j (PMC8382046; doi:10.1039/d1qo00901j)
Supplement: QO-008-D1QO00901J-s001 [file QO-008-D1QO00901J-s001.pdf]

Electronic Supplementary Material (ESI) for Organic Chemistry Frontiers.
This journal is © the Partner Organisations 2021

# Electronic Supplementary Information

## Functional group introduction and aromatic unit variation in a set of π-conjugated macrocycles: revealing the central role of local and global aromaticity

Martina Rimmele,[a,b] Wojciech Nogala,[c] Maryam Seif-Eddine,[a] Maxie M. Roessler,[a] Martin Heeney,[a,b] Felix Plasser,[d,*] Florian Glöcklhofer[a,b,*]

[a] Department of Chemistry, Imperial College London, London W12 0BZ, United Kingdom
[b] Centre for Processable Electronics, Imperial College London, London W12 0BZ, United Kingdom
[c] Institute of Physical Chemistry, Polish Academy of Sciences, Kasprzaka 44/52, 01-224 Warsaw, Poland
[d] Department of Chemistry, Loughborough University, Loughborough LE11 3TU, United Kingdom
* E-mail: f.plasser@lboro.ac.uk, f.glocklhofer@imperial.ac.uk

## Table of Contents

1 Synthesis ........ 1
2 $^{1}$H and $^{13}$C NMR spectra ........ 14
3 Photoluminescence spectra ........ 34
4 Cyclic voltammetry (CV) in solution ........ 34
5 Electron paramagnetic resonance (EPR) spectroelectrochemistry ........ 38
6 Computational analysis ........ 42
7 References ........ 55

# 1 Synthesis

Reagents and solvents were purchased from commercial suppliers and used without further purification, including compound **3a** and the halogenated precursors for the synthesis of compounds **1a-e**, with the exception of 2,6-dibromoanthracene for the synthesis of compound **1c**, which was obtained from 2,6-dibromoanthraquinone following a previously reported procedure.[1]

## 1.1 Overview of synthetic routes

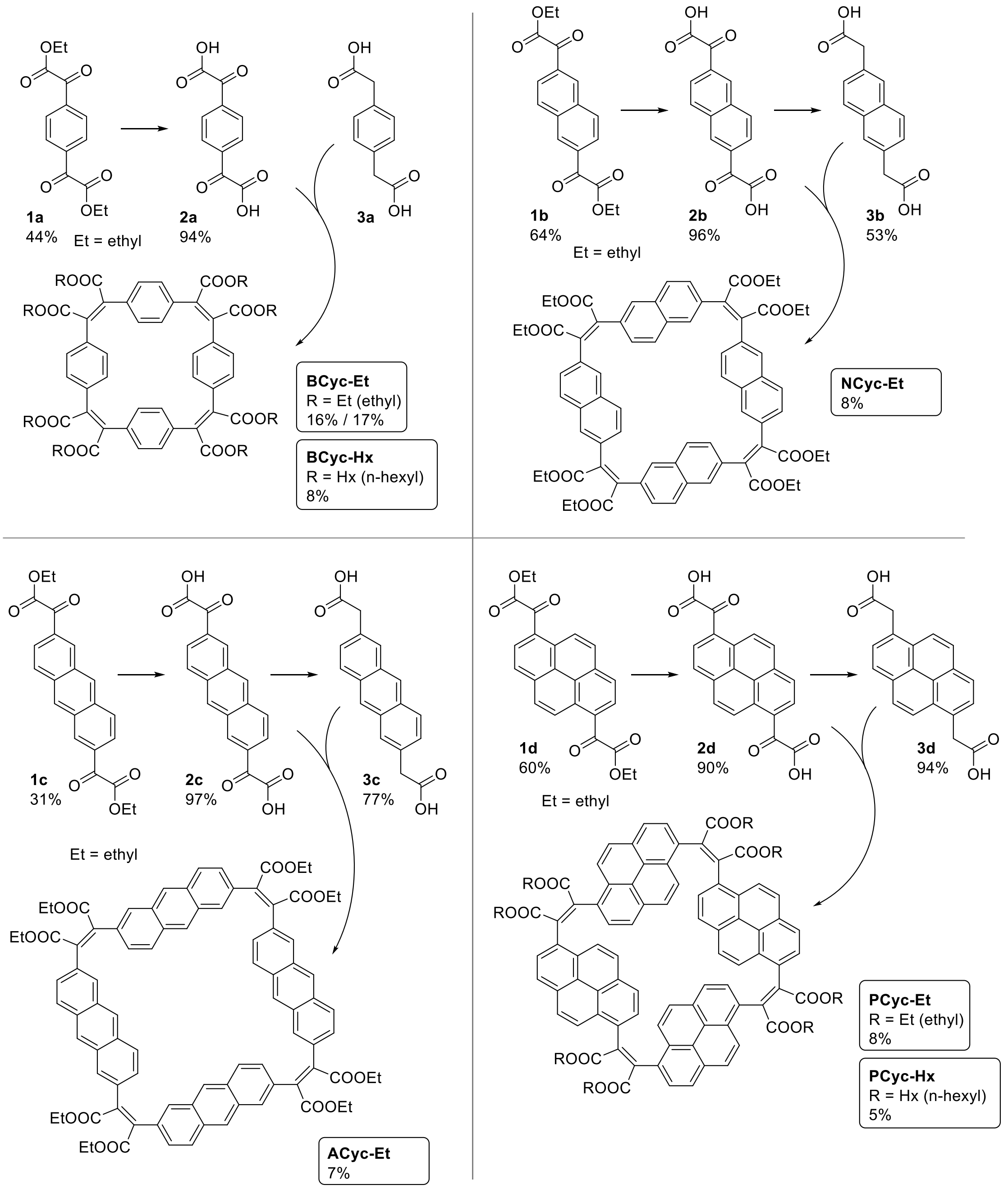


**Figure S1.** Synthetic routes to π-conjugated macrocycles with ethyl ester groups (**BCyc-Et**, **NCyc-Et**, **ACyc-Et**, **PCyc-Et**) or hexyl ester groups (**BCyc-Hx**, **PCyc-Hx**) and different aromatic units. Compounds **1a-d** were obtained from halogenated starting materials.

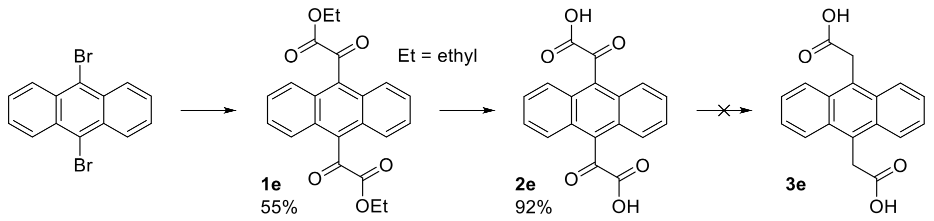


**Figure S2.** Synthetic route to compound **2e** (including the halogenated starting material) and unsuccessful conversion into compound **3e**.

## 1.2 Instrumentation

Purification by preparative GPC was carried out on a LaboACE LC-5060 (Japan Analytical Industry Co., Tokyo, JAPAN) recycling GPC system equipped with a JAIGEL-2HR column and a TOYDAD800-S detector. $CHCl_3$ was used as the eluent at a flow rate of 10 mL min$^{-1}$.
NMR spectra were recorded at 400 MHz for $^1$H and 101 MHz for $^{13}$C on a Bruker AV-400 spectrometer. High-resolution mass spectrometry was carried out on systems from Thermo Scientific for atmospheric pressure chemical ionization (APCI) and Waters for electrospray ionization (ESI). While the Thermo Scientific system gives the actual mass of the ionized compounds, the Waters system is calibrated to give the mass of the neutral compounds. This was considered when calculating the *m/z* values for comparison with the measurements.

## 1.3 Synthesis of compounds 1a-e

The procedures for synthesis of compounds **1a-e** were inspired and adapted from previous reports.[2-4]

### 1.3.1 α$^1$,α$^4$-Dioxo-1,4-benzenediacetic acid, 1,4-diethyl ester (1a)

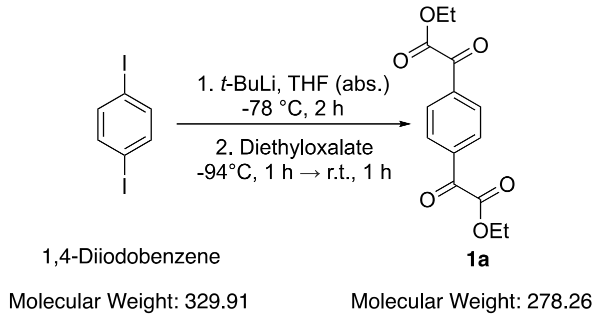


1,4-Diiodobenzene (3.50 g, 10.6 mmol, 1.0 equiv.) was dissolved in 150 mL dry degassed THF under nitrogen and cooled to -78 °C with an acetone/dry ice bath. *t*-BuLi (1.7 M in pentane, 25 mL, 42.5 mmol, 4.0 equiv.) was then added slowly by cannula transfer under vigorous stirring. The resulting mixture was kept at -78 °C for 2 h and was then cooled to -94 °C with an acetone/$N_2$(liq.) bath for the quick addition of diethyl oxalate (14 mL, 105 mmol, 9.9 equiv.). The reaction was kept at -94 °C for 1 h and was then allowed to slowly warm to r.t. After stirring at r.t. for 1 h, the reaction was poured into 2 M aqueous HCl (200 mL) and extracted three times with $CH_2Cl_2$. The combined organic phases were extracted with 2 x 150 mL sat. aqu. $Na_2SO_3$ solution and dried over $MgSO_4$. The solvent was evaporated

under reduced pressure and the remaining diethyl oxalate was distilled off under high vacuum. The compound was purified by column chromatography on silica gel using $CH_2Cl_2$/petroleum ether (1:1) as eluent. For removing trace amounts of diethyl oxalate, the now crystalline product was suspended in hexane and filtered off, yielding compound **1a** as pure, slightly yellow crystals (1.30 g, 4.67 mmol, 44%). $^1$H NMR (400 MHz, $CDCl_3$): δ = 8.16 (s, 4H), 4.47 (q, *J* = 7.1 Hz, 4H), 1.44 (t, *J* = 7.2 Hz, 6H) ppm. $^{13}$C NMR (101 MHz, $CDCl_3$): δ = 185.3, 162.9, 136.9, 130.4, 63.0, 14.2 ppm. NMR spectra in accordance with the literature.[5] HRMS (ESI): *m/z* calcd for $C_{12}H_9O_6^-$: 249.0399 [$M$−$C_2H_5$]; found 249.0390.

### 1.3.2 α²,α⁶-Dioxo-2,6-naphthalenediacetic acid, 2,6-diethyl ester (1b)

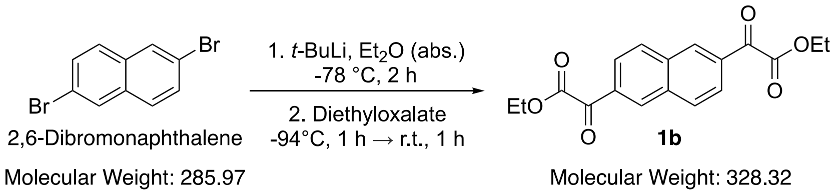


2,6-Dibromonaphthalene (3.04 g, 10.6 mmol, 1.0 equiv.) was dissolved in 150 mL dry degassed $Et_2O$ under nitrogen and cooled to -78 °C with an acetone/dry ice bath. *t*-BuLi (1.7 M in pentane, 25 mL, 42.5 mmol, 4.0 equiv.) was then added slowly by cannula transfer under vigorous stirring. The resulting mixture was kept at -78 °C for 2 h and was then cooled to -94 °C with an acetone/$N_2$(liq.) bath for the quick addition of diethyl oxalate (14 mL, 105 mmol, 9.9 equiv.). The reaction was kept at -94 °C for 1 h and was then allowed to slowly warm to r.t. After stirring at r.t. for 1 h, the reaction was poured into 2 M aqueous HCl (200 mL) and extracted three times with $CH_2Cl_2$. The combined organic phases were dried over $MgSO_4$, the solvent was evaporated under reduced pressure, and the remaining diethyl oxalate was distilled off under high vacuum. The compound was purified by column chromatography on silica gel using $CH_2Cl_2$/petroleum ether (1:1) as eluent. For removing trace amounts of diethyl oxalate, the now crystalline product was suspended in hexane and filtered off, yielding compound **1b** as pure white powder (2.24 g, 6.82 mmol, 64%). $^1$H NMR (400 MHz, $CDCl_3$): δ = 8.62 (m, 2H), 8.15 (dd, *J* = 8.6, 1.6 Hz, 2H), 8.10 (d, *J* = 8.6 Hz, 2H), 4.52 (q, *J* = 7.1 Hz, 4H), 1.47 (t, *J* = 7.1 Hz, 6H) ppm. $^{13}$C NMR (101 MHz, $CDCl_3$): δ = 185.8, 163.4, 135.4, 132.8, 132.7, 131.0, 125.5, 62.8, 14.3 ppm. HRMS (APCI): *m/z* calcd for $C_{18}H_{17}O_6^+$: 329.1020 [$M$+H]$^+$; found 329.1020.

### 1.3.3 α²,α⁶-Dioxo-2,6-anthracenediacetic acid, 2,6-diethyl ester (1c)

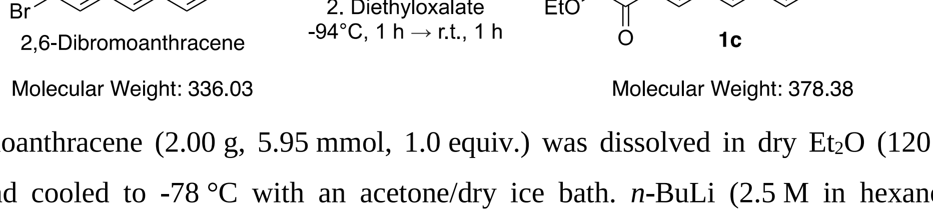


2,6-Dibromoanthracene (2.00 g, 5.95 mmol, 1.0 equiv.) was dissolved in dry $Et_2O$ (120 mL) under nitrogen and cooled to -78 °C with an acetone/dry ice bath. *n*-BuLi (2.5 M in hexanes, 9.5 mL,

23.8 mmol, 4.0 equiv.) was then added slowly under vigorous stirring. The resulting mixture was kept at -78 °C for 1 h and was then allowed to warm to r.t. After 2 h at r.t., the reaction was cooled to -94 °C with an acetone/$N_2$(liq.) bath for the quick addition of diethyl oxalate (8.0 mL, 58.9 mmol, 9.9 equiv.). The reaction was kept at -94 °C for 1 h and was then allowed to slowly warm to r.t. After stirring at r.t. for 1 h, the reaction was poured into 2 M aqueous HCl (200 mL) and extracted three times with $CH_2Cl_2$. The combined organic phases were dried over $MgSO_4$ and the solvent was evaporated under reduced pressure. The resulting crude product was filtered over a thick pad of silica using petroleum ether/$CH_2Cl_2$ (1:1) to elute diethyl oxalate and other impurities before eluting the pure product with $CH_2Cl_2$. After evaporation of the solvent, compound **1c** was obtained as pure yellow powder (0.69 g, 1.82 mmol, 31%). $^1$H NMR (400 MHz, $CDCl_3$): δ = 8.81 (d, *J* = 1.8 Hz, 2H), 8.65 (s, 2H), 8.14 (d, *J* = 8.9 Hz, 2H), 8.07 (dd, *J* = 8.9, 1.7 Hz, 2H), 4.54 (q, *J* = 7.2 Hz, 4H), 1.49 (t, *J* = 7.2 Hz, 6H) ppm. $^{13}$C NMR (101 MHz, $CDCl_3$): δ = 186.0, 163.7, 135.3, 133.7, 132.3, 131.1, 130.1, 129.9, 123.4, 62.8, 14.3 ppm. HRMS (APCI): *m/z* calcd for $C_{22}H_{19}O_6^+$: 379.1176 [*M*+H]$^+$; found: 379.1167.

### 1.3.4 α$^1$,α$^6$-Dioxo-1,6-pyrenediacetic acid, 1,6-diethyl ester (1d)

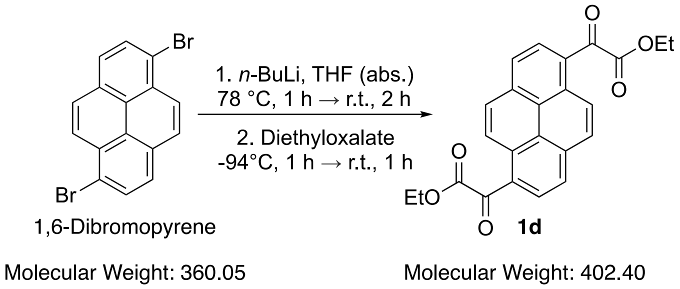


1,6-Dibromopyrene (3.00 g, 8.33 mmol, 1.0 equiv.) was dissolved in dry THF (150 mL) under nitrogen and cooled to -78 °C with an acetone/dry ice bath. *n*-BuLi (1.6 M in hexanes, 20.3 mL, 32.5 mmol, 3.9 equiv.) was then added slowly under vigorous stirring. The resulting mixture was kept at -78 °C for 1 h and was then allowed to warm to r.t. After 2 h at r.t., the reaction was cooled to -94 °C with an acetone/$N_2$(liq.) bath for the quick addition of diethyl oxalate (11 mL, 82.5 mmol, 9.9 equiv.). The reaction was kept at -94 °C for 1 h and was then allowed to slowly warm to r.t. After stirring at r.t. for 1 h, the reaction was poured into 2 M aqueous HCl (100 mL) and extracted three times with $CH_2Cl_2$. The combined organic phases were dried over $MgSO_4$ and the solvent was evaporated under reduced pressure. The resulting crude product was recrystallized from EtOH, yielding compound **1d** as pure, bright orange powder (2.02 g, 5.02 mmol, 60%). $^1$H NMR (400 MHz, $CDCl_3$): δ = 9.39 (d, *J* = 9.3 Hz, 2H), 8.43 (d, *J* = 8.1 Hz, 2H), 8.34 (d, *J* = 8.2 Hz, 2H), 8.31 (d, *J* = 9.4 Hz, 2H), 4.56 (q, *J* = 7.1 Hz, 4H), 1.50 (t, *J* = 7.2 Hz, 6H) ppm. $^{13}$C NMR (101 MHz, $CDCl_3$): δ = 189.1, 164.7, 134.6, 131.5, 130.67, 130.64, 127.8, 126.7, 126.0, 124.5, 62.9, 14.4 ppm. NMR spectra in good agreement with the literature.[4] HRMS (APCI): *m/z* calcd for $C_{24}H_{19}O_6^+$: 403.1176 [*M*+H]$^+$; found: 403.1177.

### 1.3.5 α⁹,α¹⁰-Dioxo-9,10-anthracenediacetic acid, 9,10-diethyl ester (1e)

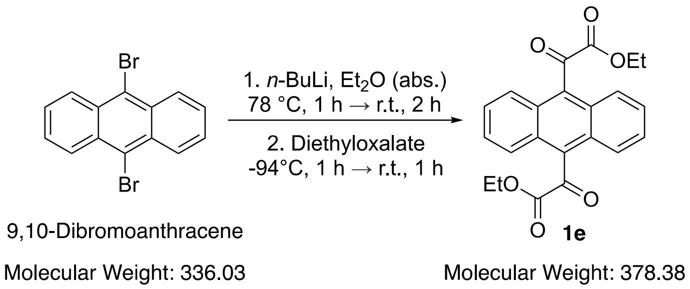


9,10-Dibromoanthracene (1.50 g, 4.46 mmol, 1.0 equiv.) was dissolved in dry $Et_2O$ (80 mL) under nitrogen and cooled to -78 °C with an acetone/dry ice bath. *n*-BuLi (2.5 M in hexanes, 11 mL, 26.8 mmol, 6.0 equiv.) was then added slowly under vigorous stirring. The resulting mixture was kept at -78 °C for 1 h and was then allowed to warm to r.t. After 2 h at r.t., the reaction was cooled to -94 °C with an acetone/$N_2$(liq.) bath for the quick addition of diethyl oxalate (6.0 mL, 44.2 mmol, 9.9 equiv.). The reaction was kept at -94 °C for 1 h and was then allowed to slowly warm to r.t. After stirring at r.t. for 1 h, the reaction was poured into 2 M aqueous HCl (200 mL) and extracted three times with $CH_2Cl_2$. The combined organic phases were dried over $MgSO_4$ and the solvent was evaporated under reduced pressure. The resulting crude product was recrystallized from EtOH, yielding compound **1e** as pure, bright yellow powder (0.93 g, 2.46 mmol, 55%). $^1$H NMR (400 MHz, $CDCl_3$): δ = 7.98 – 7.87 (m, 4H), 7.64 – 7.56 (m, 4H), 4.38 (q, $J$ = 7.1 Hz, 4H), 1.32 (t, $J$ = 7.1 Hz, 6H) ppm. $^{13}$C NMR (101 MHz, $CDCl_3$): δ = 192.3, 162.3, 134.1, 128.3, 127.8, 124.9, 63.4, 14.1 ppm. HRMS (APCI): *m/z* calcd for $C_{22}H_{19}O_6^+$: 379.1176 [$M$+H]$^+$; found: 379.1174.

## 1.4 Synthesis of compounds 2a-e

The synthesis of compounds **2a-e** was carried out using standard procedures for the saponification of carboxylic esters. The procedure for the synthesis of **2c-e** was further inspired by previous reports.[2-4]

### 1.4.1 α¹,α⁴-Dioxo-1,4-benzenediacetic acid (2a)

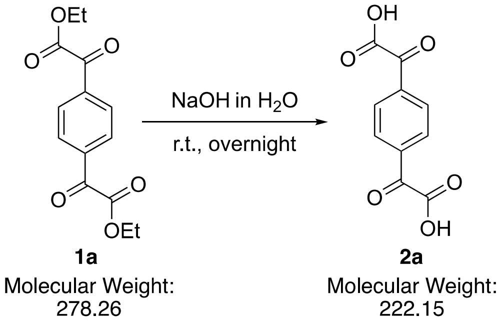


2 M aqueous NaOH (4.0 mL) was added to compound **1a** (0.40 g, 1.44 mmol, 1.0 equiv.) and the mixture stirred at r.t. overnight. The mixture was acidified to pH 1 with 4 M aqueous HCl and extracted three times with $Et_2O$. The combined organic layers were dried over $MgSO_4$ and the solvent was evaporated under reduced pressure to give compound **2a** as a white powder (0.30 g, 1.35 mmol, 94%).

$^1H$ NMR (400 MHz, DMSO-$d_6$): δ = 8.14 (s, 4H) ppm. $^{13}C$ NMR (101 MHz, DMSO-$d_6$): δ = 187.8, 165.0, 136.3, 130.1 ppm. HRMS (ESI): *m/z* calcd for $C_{10}H_5O_6^-$: 221.0086 [*M*−H]; found: 221.0093.

### 1.4.2 α²,α⁶-Dioxo-2,6-naphthalenediacetic acid (2b)

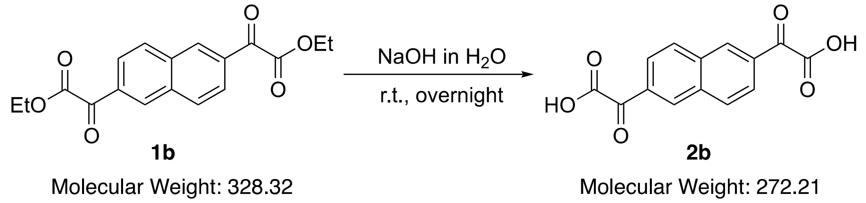


2 M aqueous NaOH (30 mL) was added to compound **1b** (1.50 g, 4.57 mmol, 1.0 equiv.) and the mixture stirred at r.t. overnight. The mixture was acidified to pH 1 with 4 M aqueous HCl and extracted three times with $Et_2O$. The combined organic layers were dried over $MgSO_4$ and the solvent was evaporated under reduced pressure to give compound **2b** as a white powder (1.20 g, 4.41 mmol, 96%). $^1H$ NMR (400 MHz, DMSO-$d_6$): δ = 8.72 (d, J = 2.0 Hz, 2H), 8.42 (d, *J* = 8.5 Hz, 2H), 8.08 (dd, *J* = 8.5, 1.7 Hz, 2H) ppm. $^{13}C$ NMR (101 MHz, DMSO-$d_6$): δ = 188.4, 165.6, 134.8, 132.2, 132.0, 131.2, 124.8 ppm. HRMS (ESI): *m/z* calcd for $C_{14}H_7O_6^-$: 271.0243 [*M*−H]; found: 271.0236.

### 1.4.3 α²,α⁶-Dioxo-2,6-anthracenediacetic acid (2c)

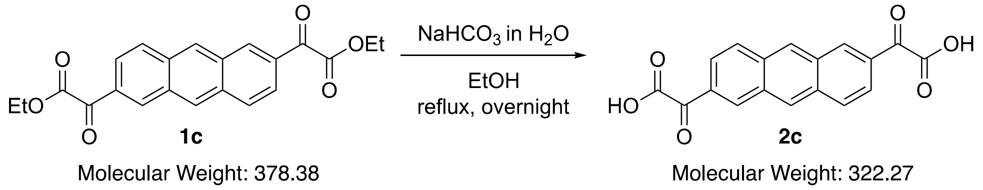


Compound **1c** (0.44 g, 1.16 mmol, 1.0 equiv.) was suspended in 30 mL EtOH. $NaHCO_3$ (2.63 g, 31.3 mmol, 27 equiv.) dissolved in 15 mL $H_2O$ was then added and the reaction was heated to reflux overnight. The reaction was then allowed to cool to r.t., poured into 150 mL 2 M aqueous HCl, and extracted three times with $Et_2O$. The combined organic phases were dried over $MgSO_4$ and the solvent was evaporated under reduced pressure. For removing residues of $H_2O$, the compound was redissolved in acetone and the solvent was evaporated again under reduced pressure to give compound **2c** as a red powder (0.36 g, 1.12 mmol, 97%). $^1H$ NMR (400 MHz, DMSO-$d_6$): δ = 9.07 (s, 2H), 8.88 (d, *J* = 1.7 Hz, 2H), 8.31 (d, *J* = 9.0 Hz, 2H), 7.99 (dd, *J* = 8.9, 1.7 Hz, 2H) ppm. $^{13}C$ NMR (101 MHz, DMSO-$d_6$): δ = 188.5, 165.9, 134.7, 133.0, 131.7, 130.2, 130.1 (two C according to HSQC spectrum), 122.6 ppm. HRMS (ESI): *m/z* calcd for $C_{18}H_9O_6^-$: 321.0399 [*M*−H]; found: 321.0405.

### 1.4.4 α¹,α⁶-Dioxo-1,6-pyrenediacetic acid (2d)

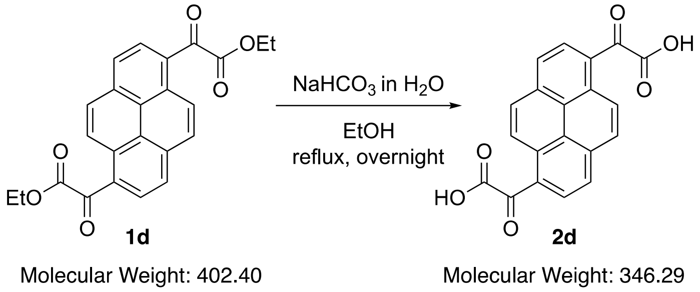


Compound **1d** (2.30 g, 5.72 mmol, 1.0 equiv.) was suspended in 60 mL EtOH. $NaHCO_3$ (13.0 g, 154 mmol, 27 equiv.) dissolved in 160 mL $H_2O$ was then added and the reaction was heated to reflux overnight. The reaction was then allowed to cool to r.t., poured into 200 mL 2 M aqueous HCl, and the resulting precipitate was filtered off using a sintered glass funnel. For removing residues of $H_2O$, the compound was dissolved in acetone and the solvent was evaporated under reduced pressure to give compound **2d** as a red powder (1.78 g, 5.14 mmol, 90%). $^1$H NMR (400 MHz, DMSO-$d_6$): δ = 9.24 (d, *J* = 9.3 Hz, 2H), 8.58 (d, *J* = 8.1 Hz, 2H), 8.56 – 8.49 (m, 4H) ppm.$^{13}$C NMR (101 MHz, DMSO-$d_6$): δ = 191.2, 166.2, 133.7, 130.6 (two C according to HSQC spectrum), 130.1, 126.7, 126.5, 126.4, 123.4 ppm. NMR spectra in accordance with the literature.[4] HRMS (ESI): *m/z* calcd for $C_{20}H_9O_6^-$: 345.0399 [*M*−H]; found: 345.0405.

### 1.4.5 α⁹,α¹⁰-Dioxo-9,10-anthracenediacetic acid (2e)

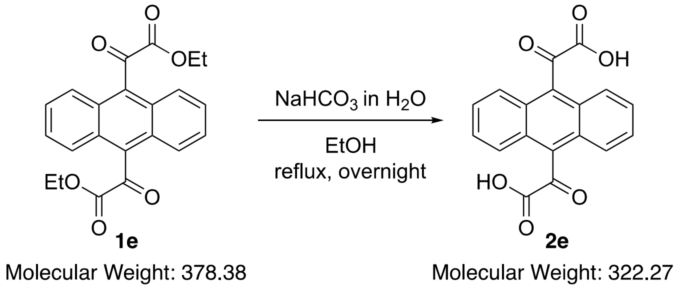


Compound **1e** (1.83 g, 4.84 mmol, 1.0 equiv.) was suspended in 100 mL EtOH. $NaHCO_3$ (11.0 g, 131 mmol, 27 equiv.) dissolved in 270 mL $H_2O$ was then added and the reaction was heated to reflux overnight. The reaction was then allowed to cool to r.t., poured into 200 mL 2 M aqueous HCl, and extracted three times with $Et_2O$. The combined organic phases were dried over $MgSO_4$ and the solvent was evaporated under reduced pressure to give compound **2e** as a yellow powder (1.44 g, 4.47 mmol, 92%). $^1$H NMR (400 MHz, DMSO-$d_6$): δ = 7.97 – 7.88 (m, 4H), 7.81 – 7.65 (m, 4H) ppm. $^{13}$C NMR (101 MHz, DMSO-$d_6$): δ = 194.4, 163.6, 134.3, 128.0, 127.1, 124.6. HRMS (ESI): *m/z* calcd for $C_{17}H_9O_4^-$: 277.0501 [*M*−COOH]; found: 277.0510.

## 1.5 Synthesis of compounds 3b-d

The synthesis of compounds **3b-d** was carried out by adapting previously reported procedures.[6,7]

**Notes:** (i) Compound **3a** was obtained from commercial suppliers. (ii) The synthesis of compound **3e** was not successful following the procedure used for the synthesis of compounds **3b-d**.

### 1.5.1 2,6-Naphthalenediacetic acid (3b)

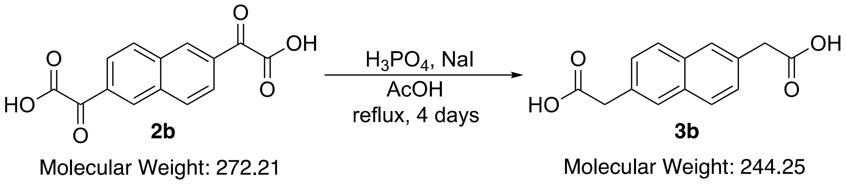


Compound **2b** (0.50 g, 1.84 mmol, 1.0 equiv.) and NaI (0.33 g, 2.20 mmol, 1.2 equiv.) were dissolved in 10 mL acetic acid in a reaction vial equipped with a septum. $H_3PO_2$ (50 wt% in $H_2O$, 0.5 mL, 4.78 mmol, 2.6 equiv.) was added and the mixture was degassed with nitrogen for 15 min and subsequently heated to reflux under nitrogen for 4 days. After cooling to r.t., water (2 mL) was added and the precipitate was filtered off and dried *in vacuo*, yielding compound **3b** (0.24 g, 0.98 mmol, 53%) as a white powder. $^{1}$H NMR (400 MHz, DMSO-$d_6$): δ = 12.35 (bs, 2H), 7.81 (d, *J* = 8.4 Hz, 2H), 7.74 (m, 2H), 7.41 (dd, *J* = 8.4, 1.6 Hz, 2H), 3.73 (s, 4H) ppm. $^{13}$C NMR (101 MHz, DMSO-$d_6$): δ = 172.7, 132.4, 131.8, 128.1, 127.4, 127.3, 40.8 ppm. NMR spectra in good agreement with spectra measured in acetone-$d_6$.[8] HRMS (ESI): *m/z* calcd for $C_{14}H_{11}O_4^-$: 243.0657 [*M*−H], found: 243.0661.

### 1.5.2 2,6-Anthracenediacetic acid (3c)

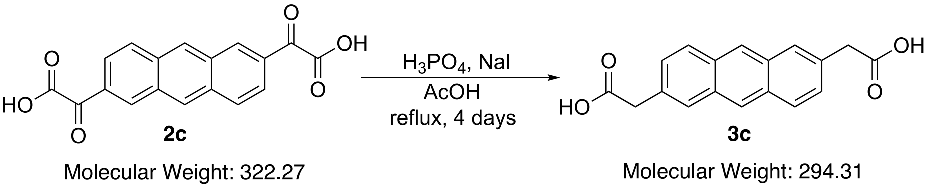


Compound **2c** (0.50 g, 1.55 mmol, 1.0 equiv.) and NaI (0.22 g, 1.47 mmol, 0.95 equiv.) were dissolved in 12.5 mL acetic acid in a reaction vial equipped with a septum. $H_3PO_2$ (50 wt% in $H_2O$, 0.5 mL, 4.8 mmol, 3.1 equiv.) was added and the mixture was degassed with nitrogen for 15 min and subsequently heated to reflux under nitrogen for 4 days. After cooling to r.t., water (5 mL) was added and the precipitate was filtered off and dried *in vacuo*, yielding compound **3c** (0.35 g, 1.19 mmol, 77%) as an off-white powder. $^{1}$H NMR (400 MHz, DMSO-$d_6$): δ = 12.41 (bs, 2H), 8.48 (s, 2H), 8.03 (d, *J* = 8.7 Hz, 2H), 7.92 (d, *J* = 1.6 Hz, 2H), 7.42 (dd, *J* = 8.7, 1.7 Hz, 2H), 3.79 (s, 4H) ppm. $^{13}$C NMR (101 MHz, DMSO-$d_6$): δ = 172.6, 132.2, 131.0, 130.4, 127.90, 127.86 127.5, 125.3, 40.9 ppm. HRMS (APCI): *m/z* calcd. for $C_{18}H_{15}O_4^+$: 295.0965 [*M*+H]$^+$; found: 295.0960.

### 1.5.3 1,6-Pyrenediacetic acid (3d)

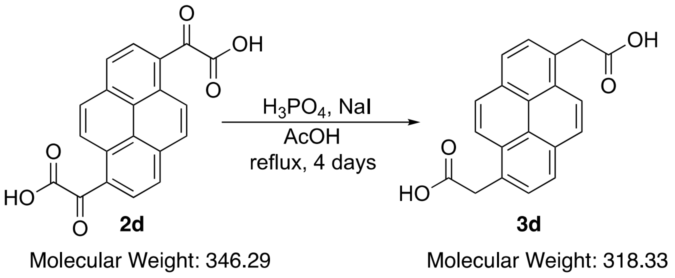


Compound **2d** (0.45 g, 1.30 mmol, 1.0 equiv.) and NaI (0.19 g, 1.27 mmol, 0.98 equiv.) were dissolved in 5.7 mL acetic acid in a reaction vial equipped with a septum. $H_3PO_2$ (50 wt% in $H_2O$, 0.44 mL, 4.0 mmol, 3.1 equiv.) was added and the mixture was degassed with nitrogen for 15 min and subsequently heated to reflux under nitrogen for 4 days. After cooling to r.t., THF (17 mL) was added and the precipitate was filtered off and dried *in vacuo*, yielding compound **3d** (0.39 g, 1.23 mmol, 94%) as a pale-yellow powder. $^{1}H$ NMR (400 MHz, DMSO-$d_6$): δ = 12.51 (bs, 2H), 8.30 – 8.18 (m, 6H), 8.01 (d, $J$ = 7.9 Hz, 2H), 4.35 (s, 4H) ppm. $^{13}C$ NMR (101 MHz, DMSO-$d_6$): δ = 172.8, 129.8, 129.6, 129.2, 128.9, 127.5, 124.8, 124.2, 123.3 ppm (note: methylene carbon signal hidden by solvent signal). NMR spectra in accordance with the literature.[7] HRMS (APCI): $m/z$ calcd. for $C_{20}H_{15}O_4^+$: 319.0965 $[M+H]^+$; found: 319.0962.

## 1.6 Synthesis of the *π*-conjugated macrocycles

The macrocycles were prepared adapting a previously reported procedure.[7]

### 1.6.1 General procedure

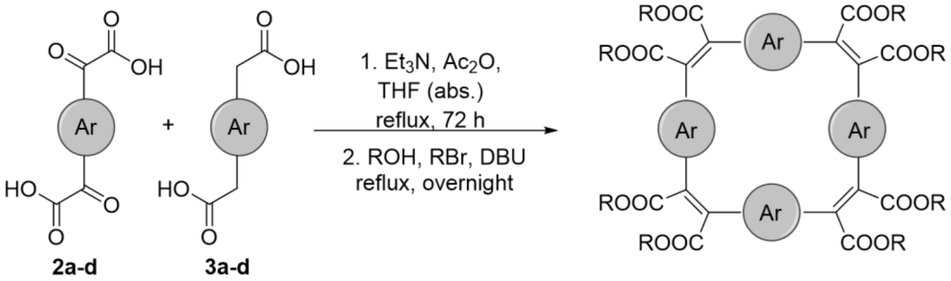


To a mixture of the respective dioxodiacetic acid **2a-d** (1.0 equiv.) and diacetic acid **3a-d** (1.0 equiv.) in varying amounts of dry degassed THF under nitrogen, triethylamine (9.5 equiv., $Et_3N$) and acetic anhydride (15 equiv., $Ac_2O$) were added. The reaction mixture was heated to reflux under nitrogen for 3 days before the respective alkyl bromide (44 equiv., RBr), the respective alcohol (82 equiv., ROH), and 1,8-diazabicyclo[5.4.0]undec-7-ene (21 equiv., DBU) were added. After refluxing for another 24 h, the reaction was allowed to cool to r.t., poured into 1 M aqueous HCl (350 mL), extracted three times with $CHCl_3$, and dried over $MgSO_4$. Evaporation of the solvent under reduced pressure gave the crude product, which was split into two fractions for purification by gel permeation chromatography (GPC).

Each fraction was dissolved in $CHCl_3$ and filtered through a 0.2 µm syringe filter before loading the GPC column with the resulting solution ($CHCl_3$ was used as eluent for the GPC).

*Alternative procedure using a syringe pump for the addition of the dioxodiacetic and diacetic acid:*
To a refluxing mixture of $Et_3N$ (9.5 equiv.) and $Ac_2O$ (15 equiv.) in dry degassed THF (400 mL) under nitrogen, the respective dioxodiacetic acid (1.0 equiv.) and diacetic acid (1.0 equiv.) dissolved in 20 mL dry degassed THF were added over 10 h (2 mL $h^{-1}$) using a syringe pump. The reaction was refluxed for 3 days (starting with the addition of the reactants) before adding the respective alkyl bromide (44 equiv., RBr), the respective alcohol (82 equiv., ROH) and DBU (21 equiv.) and proceeding with next steps in accordance with the general procedure described above.

### 1.6.2 Macrocycle BCyc-Et

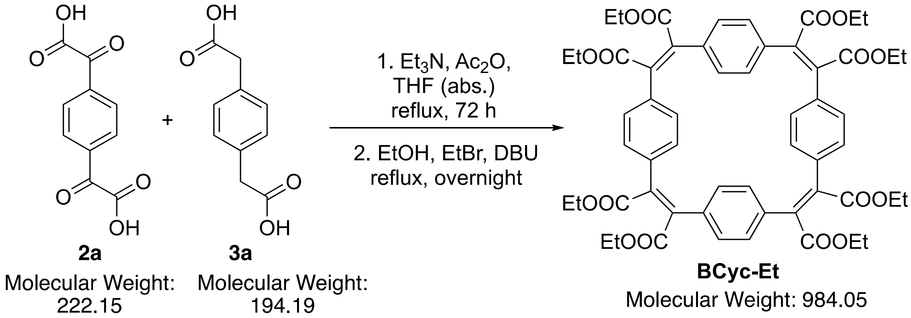


*Synthesis of macrocycle **BCyc-Et** following the general procedure:*
$\alpha^1,\alpha^4$-Dioxo-1,4-benzenediacetic acid **2a** (250 mg, 1.13 mmol, 1.0 equiv.), 1,4-benzenediacetic acid **3a** (219 mg, 1.13 mmol, 1.0 equiv.), dry degassed THF (400 mL), $Et_3N$ (1.5 mL, 10.7 mmol, 9.5 equiv.), $Ac_2O$ (1.6 mL, 16.9 mmol, 15 equiv.), EtBr (3.7 mL, 49.5 mmol, 44 equiv.), EtOH (5.4 mL, 92.3 mmol, 82 equiv.) and DBU (3.5 mL, 23.6 mmol, 21 equiv.) were used. Purification by GPC and evaporation of the solvent afforded macrocycle **BCyc-Et** as a dark yellow oil (87.9 mg, 0.089 mmol, 16%), which slowly crystallised over time and upon further drying *in vacuo* to give a yellow powder. $^1$H NMR (400 MHz, $CDCl_3$): δ = 6.93 (s, 16H), 4.31 (q, *J* = 7.1 Hz, 16H), 1.31 (t, *J* = 7.2 Hz, 24H) ppm. $^{13}$C NMR (101 MHz, $CDCl_3$): δ = 167.8, 137.5, 134.6, 129.9, 62.1, 14.2 ppm. HRMS (APCI): *m/z* calcd. for $C_{56}H_{56}O_{16}^+$: 984.3563 $[M]^+$; found: 984.3525. HRMS (ESI): *m/z* calcd. for $C_{56}H_{56}O_{16}Na^+$: 1007.3466 [*M*+Na]; found: 1007.3480.

*Synthesis of macrocycle **BCyc-Et** following the alternative procedure (using a syringe pump):*
The same amounts of reagents and reactants as for the synthesis following the general procedure were used. Purification by GPC and evaporation of the solvent afforded macrocycle **BCyc-Et** (93.6 mg, 0.095 mmol, 17%) as a dark yellow oil, which slowly crystallised over time to give a yellow powder.

### 1.6.3 Macrocycle BCyc-Hx

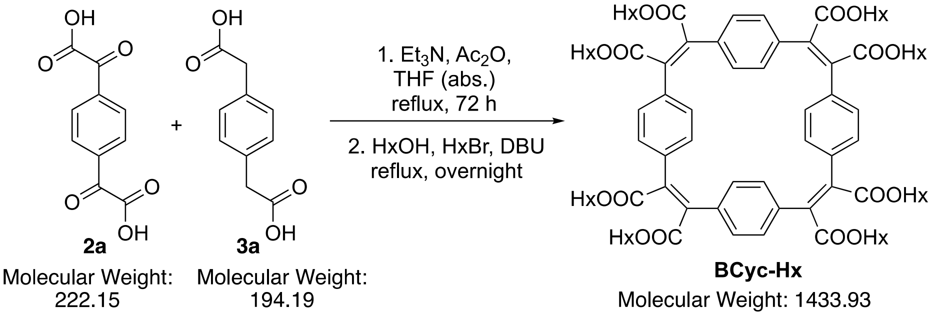


*Synthesis of macrocycle* ***BCyc-Hx*** *following the alternative procedure (using a syringe pump):*

$\alpha^1$,$\alpha^4$-dioxo-1,4-benzenediacetic acid **2a** (250 mg, 1.13 mmol, 1.0 equiv.), 1,4-benzenediacetic acid **3a** (219 mg, 1.13 mmol, 1.0 equiv.), dry degassed THF (400 mL), $Et_3N$ (1.5 mL, 10.7 mmol, 9.5 equiv.), $Ac_2O$ (1.6 mL, 16.9 mmol, 15 equiv.), 1-bromohexane (7.0 mL, 49.5 mmol, 44 equiv.), 1-hexanol (11.6 mL, 92.3 mmol, 82 equiv.) and DBU (3.5 mL, 23.7 mmol, 21 equiv.) were used. Purification by GPC and evaporation of the solvent afforded macrocycle **BCyc-Hx** (68.4 mg, 0.0477 mmol, 8%) as a brown oil. $^1$H NMR (400 MHz, $CDCl_3$): δ = 6.92 (s, 16H), 4.23 (t, *J* = 6.8 Hz, 16H), 1.66 (p, *J* = 7.0 Hz, 16H), 1.35 – 1.22 (m, 48H), 0.86 (t, *J* = 6.7 Hz, 24H) ppm. $^{13}$C NMR (101 MHz, $CDCl_3$): δ = 167.8, 137.5, 134.6, 129.9, 66.3, 31.5, 28.4, 25.6, 22.6, 14.1 ppm. HRMS (APCI): *m/z* calcd. for $C_{88}H_{120}O_{16}^+$:1432.8571 [*M*]$^+$, found: 1432.8540.

### 1.6.4 Macrocycle NCyc-Et

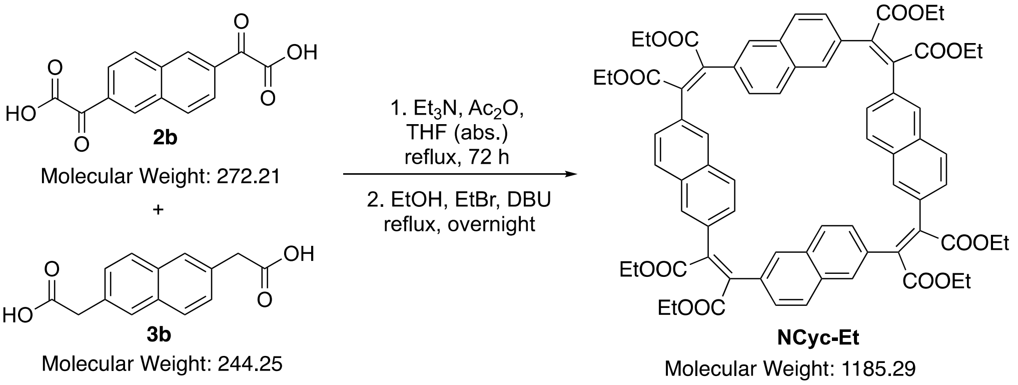


*Synthesis of macrocycle* ***NCyc-Et*** *following the general procedure:*

$\alpha^2$,$\alpha^6$-Dioxo-2,6-naphthalenediacetic acid **2b** (268 mg, 0.985 mmol, 1.0 equiv.), 2,6-naphthalene-diacetic acid **3b** (240 mg, 0.985 mmol, 1.0 equiv.), dry degassed THF (400 mL), $Et_3N$ (1.3 mL, 9.35 mmol, 9.5 equiv.), $Ac_2O$ (1.4 mL, 14.8 mmol, 15 equiv.), EtBr (3.2 mL, 43.3 mmol, 44 equiv.), EtOH (4.7 mL, 80.7 mmol, 82 equiv.) and DBU (3.1 mL, 20.7 mmol, 21 equiv.) were used. Purification by GPC and evaporation of the solvent afforded macrocycle **NCyc-Et** (46.1 mg, 0.0389 mmol, 8%) as a pale-yellow powder. $^1$H NMR (400 MHz, $CDCl_3$): δ = 7.51 (d, *J* = 1.6 Hz, 8H), 7.24 (d, *J* = 8.6 Hz, 8H), 6.88 (dd, *J* = 8.6, 1.7 Hz, 8H), 4.35 (q, *J* = 7.2 Hz, 16H), 1.32 (t, *J* = 7.2 Hz, 24H) ppm. $^{13}$C NMR

(101 MHz, $CDCl_3$): δ = 168.0, 138.4, 133.0, 132.3, 129.7, 128.1, 127.8, 62.1, 14.2 ppm. HRMS (APCI): *m/z* calcd. for $C_{72}H_{64}O_{16}^{+}$: 1184.4189 $[M]^{+}$, found: 1184.4194.

### 1.6.5 Macrocycle ACyc-Et

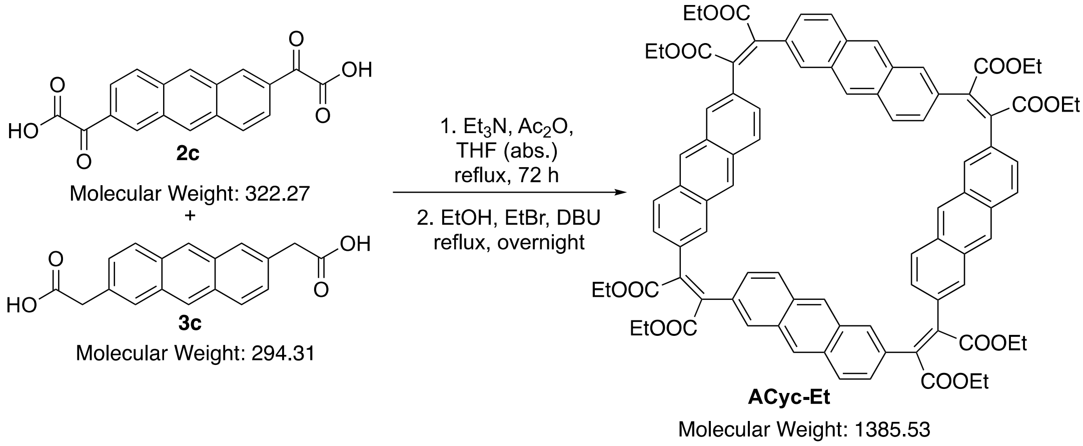


*Synthesis of macrocycle **ACyc-Et** following the general procedure:*

$\alpha^2,\alpha^6$-Dioxo-2,6-anthracenediacetic acid **2c** (250 mg, 0.776 mmol, 1.0 equiv.), 2,6-anthracenediacetic acid **3c** (228 mg, 0.776 mmol, 1.0 equiv.), dry degassed THF (400 mL), $Et_3N$ (1.0 mL, 7.37 mmol, 9.5 equiv.), $Ac_2O$ (1.1 mL, 11.6 mmol, 15 equiv.), EtBr (2.5 mL, 34.1 mmol, 44 equiv.), EtOH (3.7 mL, 63.6 mmol, 82 equiv.) and DBU (2.4 mL, 16.3 mmol, 21 equiv.) were used. Purification by GPC and evaporation of the solvent afforded macrocycle **ACyc-Et** (36.0 mg, 0.0260 mmol, 7%) as a dark orange powder. $^{1}$H NMR (400 MHz, $CDCl_3$): δ = 8.08 (s, 8H), 7.87 (d, $J$ = 1.7 Hz, 8H), 7.61 (d, $J$ = 8.9 Hz, 8H), 7.06 (dd, $J$ = 8.9, 1.8 Hz, 8H), 4.37 (q, 16H), 1.32 (t, $J$ = 7.2 Hz, 24H) ppm. $^{13}$C NMR (101 MHz, $CDCl_3$): δ = 168.3, 138.2, 132.1, 131.7, 131.1 130.4, 128.2, 127.01, 126.95, 62.0, 14.2 ppm.

**Important note:** Although we found the synthesis of all precursors and all other macrocycles to be reproducible, we struggled to re-synthesize **ACyc-Et** in later attempts. However, as highly interesting effects were observed for this macrocycle in both the computations and experiments, we decided not to omit the available data here.

### 1.6.6 Macrocycle PCyc-Et

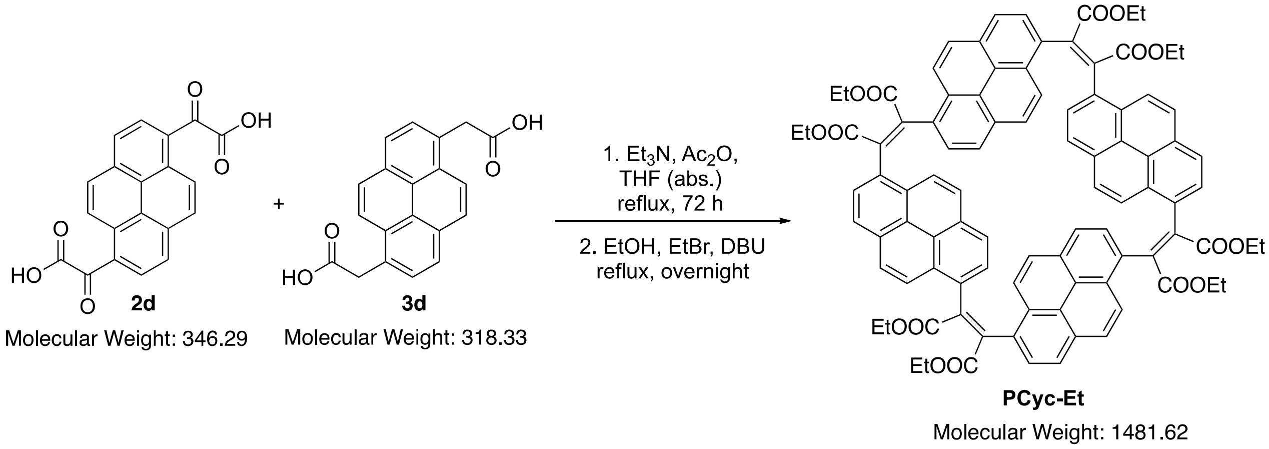

*Synthesis of macrocycle **PCyc-Et** following the general procedure:*

$\alpha^1$,$\alpha^6$-Dioxo-1,6-pyrenediacetic acid **2d** (235 mg, 0.679 mmol, 1.0 equiv.), 1,6-pyrenediacetic acid **3d** (216 mg, 0.679 mmol, 1.0 equiv.), dry degassed THF (350 mL), $Et_3N$ (0.90 mL, 6.45 mmol, 9.5 equiv.), $Ac_2O$ (0.96 mL, 10.2 mmol, 15 equiv.), EtBr (2.2 mL, 29.9 mmol, 44 equiv.), EtOH (3.2 mL, 55.6 mmol, 82 equiv.) and DBU (2.1 mL, 14.3 mmol, 21 equiv.) were used. Purification by GPC and evaporation of the solvent afforded macrocycle **PCyc-Et** (37.9 mg, 0.0256 mmol, 8%) as an orange powder. $^1$H NMR (400 MHz, $CDCl_3$): δ = 8.24 (d, *J* = 9.2 Hz, 8H), 7.86 (d, *J* = 8.0 Hz, 8H), 7.77 (d, *J* = 8.0 Hz, 8H), 7.70 (d, *J* = 9.2 Hz, 8H), 4.21 – 4.13 (m, 16H), 1.13 (t, *J* = 7.1 Hz, 24H). $^{13}$C NMR (101 MHz, $CDCl_3$): δ = 168.2, 139.8, 130.9, 129.7, 129.0, 127.9, 127.7, 124.9, 124.7, 124.6, 62.0, 14.0 ppm. NMR spectra in good agreement with the spectra in $CD_2Cl_2$ in the literature.[7] HRMS (APCI): *m/z* calcd. for $C_{96}H_{73}O_{16}^+$: 1481.4848 [*M*+H]$^+$; found: 1481.4835.

### 1.6.7 Macrocycle PCyc-Hx

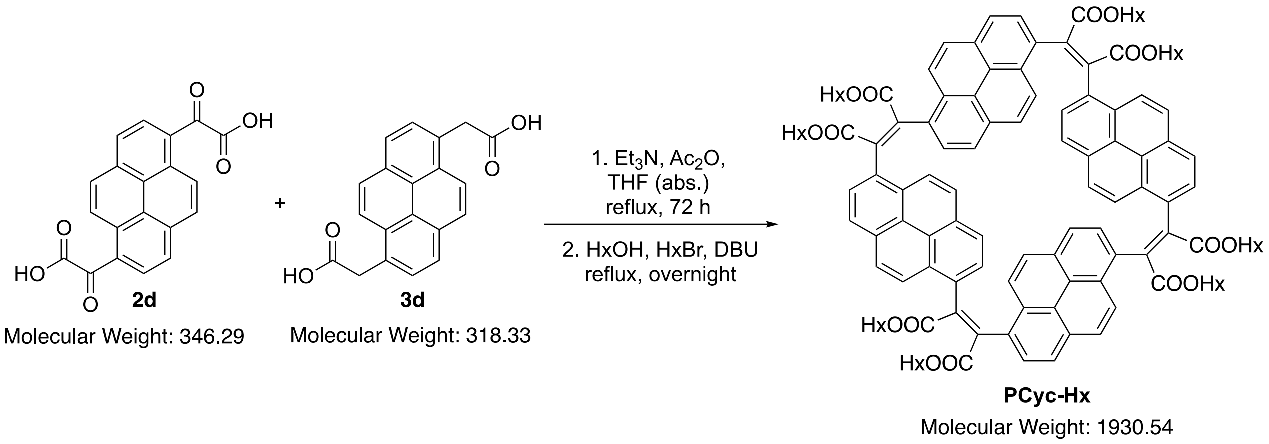


*Synthesis of macrocycle **PCyc-Et** following the general procedure:*

$\alpha^1$,$\alpha^6$-Dioxo-1,6-pyrenediacetic acid **2d** (250 mg, 0.722 mmol, 1.0 equiv.), 1,6-pyrenediacetic acid **3d** (230 mg, 0.722 mmol, 1.0 equiv.), dry degassed THF (300 mL), $Et_3N$ (0.96 mL, 6.86 mmol, 9.5 equiv.), $Ac_2O$ (1.0 mL, 10.8 mmol, 15 equiv.), 1-bromohexane (4.5 mL, 31.8 mmol, 44 equiv.), 1-hexanol (7.4 mL, 59.2 mmol, 82 equiv.) and DBU (2.3 mL, 15.2 mmol, 21 equiv.) were used. Purification by GPC and evaporation of the solvent afforded macrocycle **PCyc-Hx** (37.5 mg, 0.0194 mmol, 5%) as a red oil. $^1$H NMR (400 MHz, $CDCl_3$): δ = 8.23 (d, *J* = 9.2 Hz, 8H), 7.86 (d, *J* = 8.0 Hz, 8H), 7.77 (d, *J* = 8.0 Hz, 8H), 7.70 (d, *J* = 9.3 Hz, 8H), 4.13 – 4.00 (m, 16H), 1.45 – 1.38 (m, 16H), 1.04 – 0.88 (m, 48H), 0.69 – 0.61 (m, 24H) ppm. $^{13}$C NMR (101 MHz, $CDCl_3$): δ = 168.2, 139.9, 130.8, 129.7, 129.0, 127.9, 127.6, 124.9, 124.6, 124.5, 66.0, 31.2, 28.3, 25.4, 22.4, 13.9 ppm. HRMS (APCI): *m/z* calcd. for $C_{128}H_{137}O_{16}^+$: 1929.9856 [*M*+H]$^+$; found: 1929.9817.

## 2 $^{1}$H and $^{13}$C NMR spectra

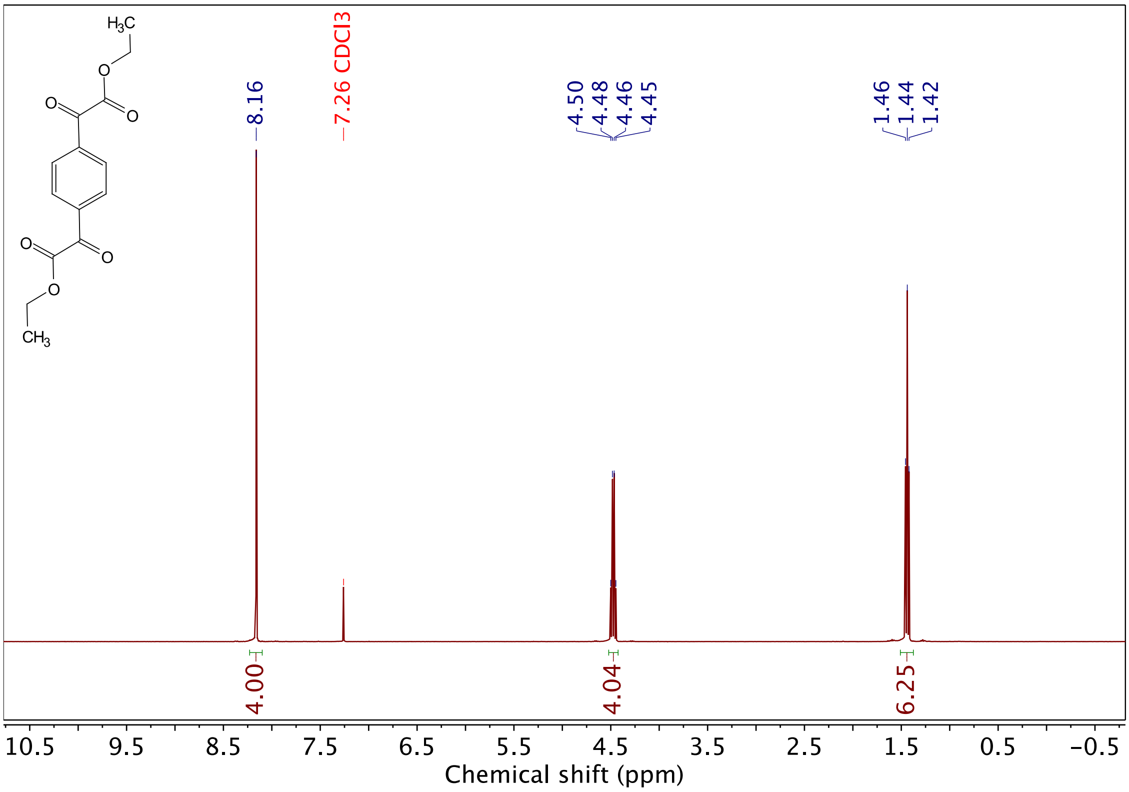


**Figure S3.** $^{1}$H NMR (400 MHz, $CDCl_3$) of compound **1a**.

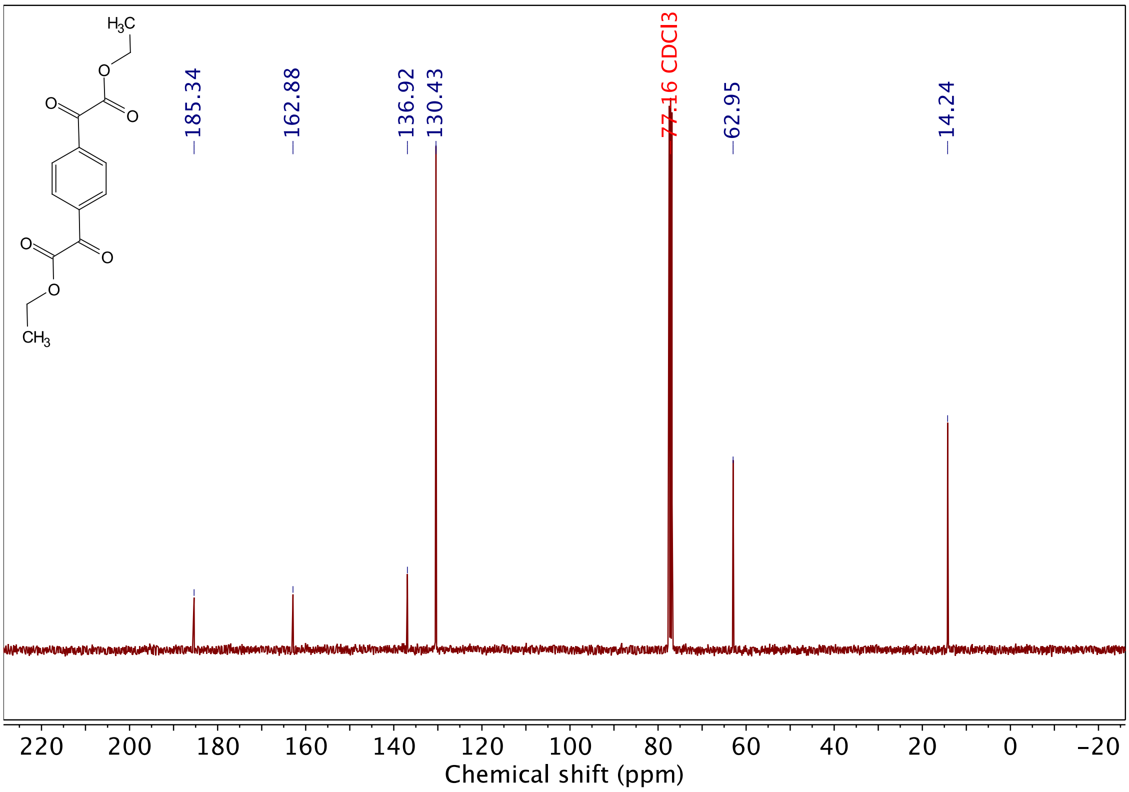


**Figure S4.** $^{13}$C NMR (101 MHz, $CDCl_3$) of compound **1a**.

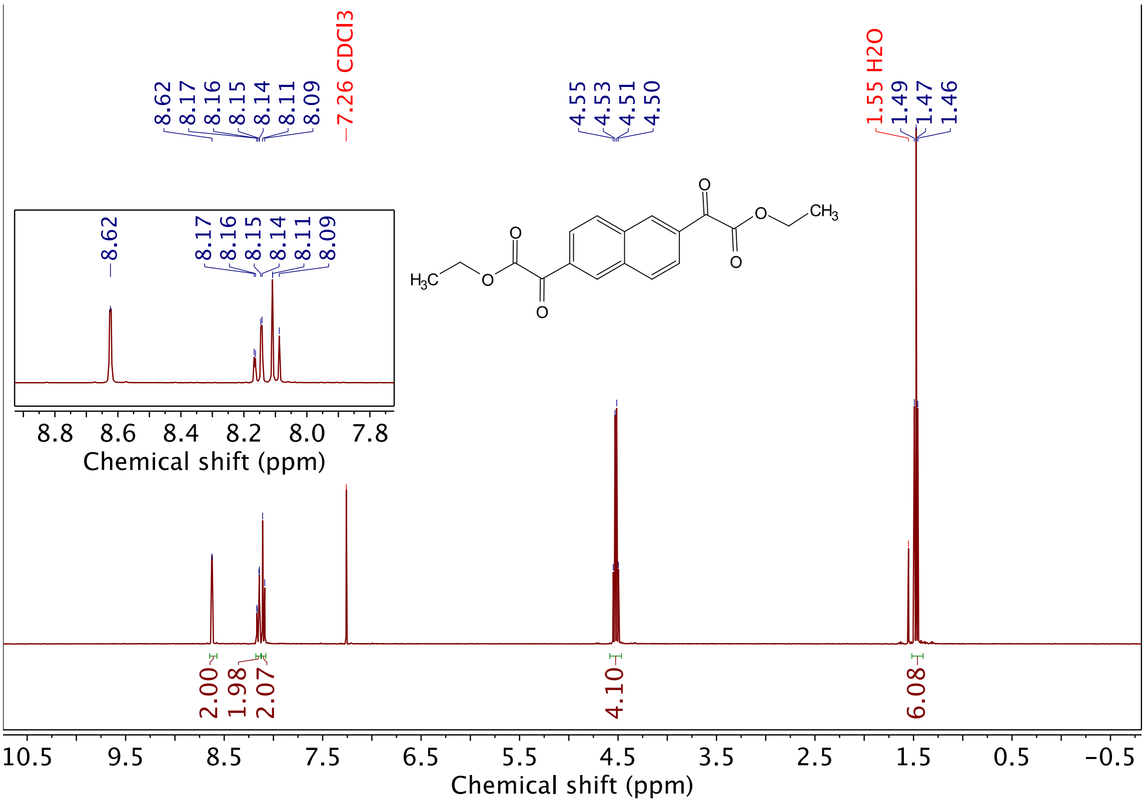


**Figure S5.** $^{1}H$ NMR (400 MHz, $CDCl_3$) of compound **1b**.

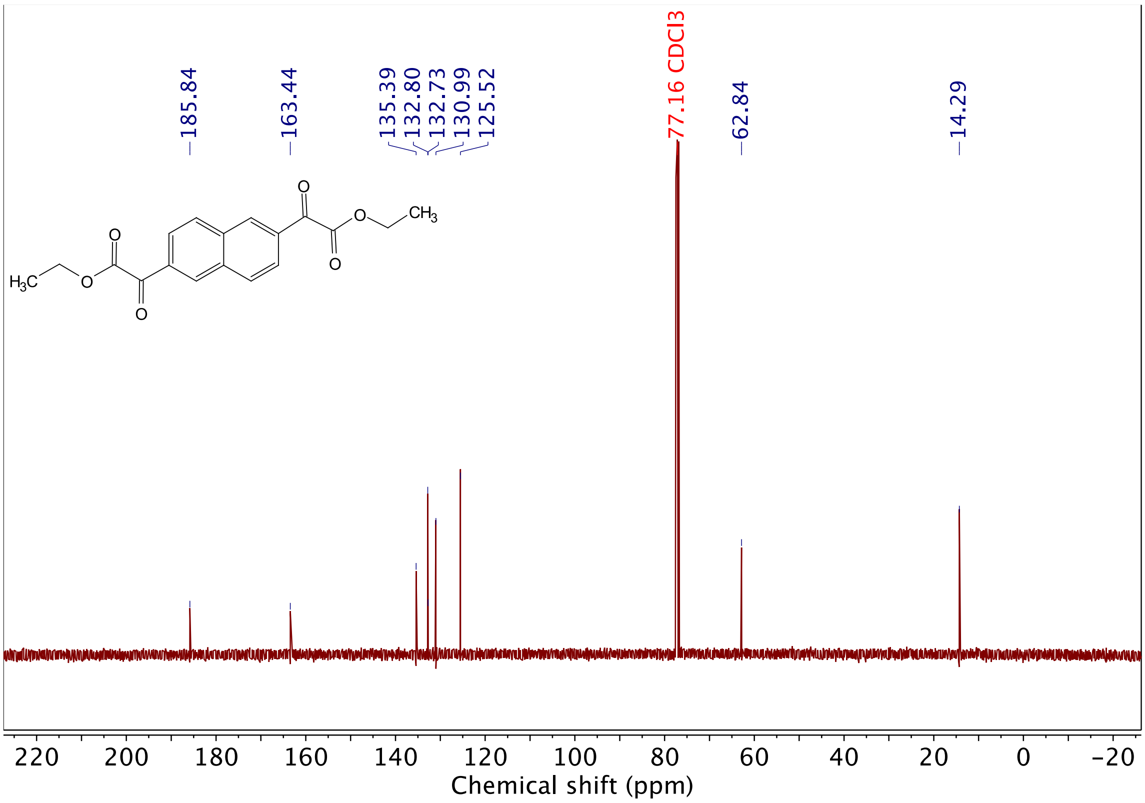


**Figure S6.** $^{13}C$ NMR (101 MHz, $CDCl_3$) of compound **1b**.

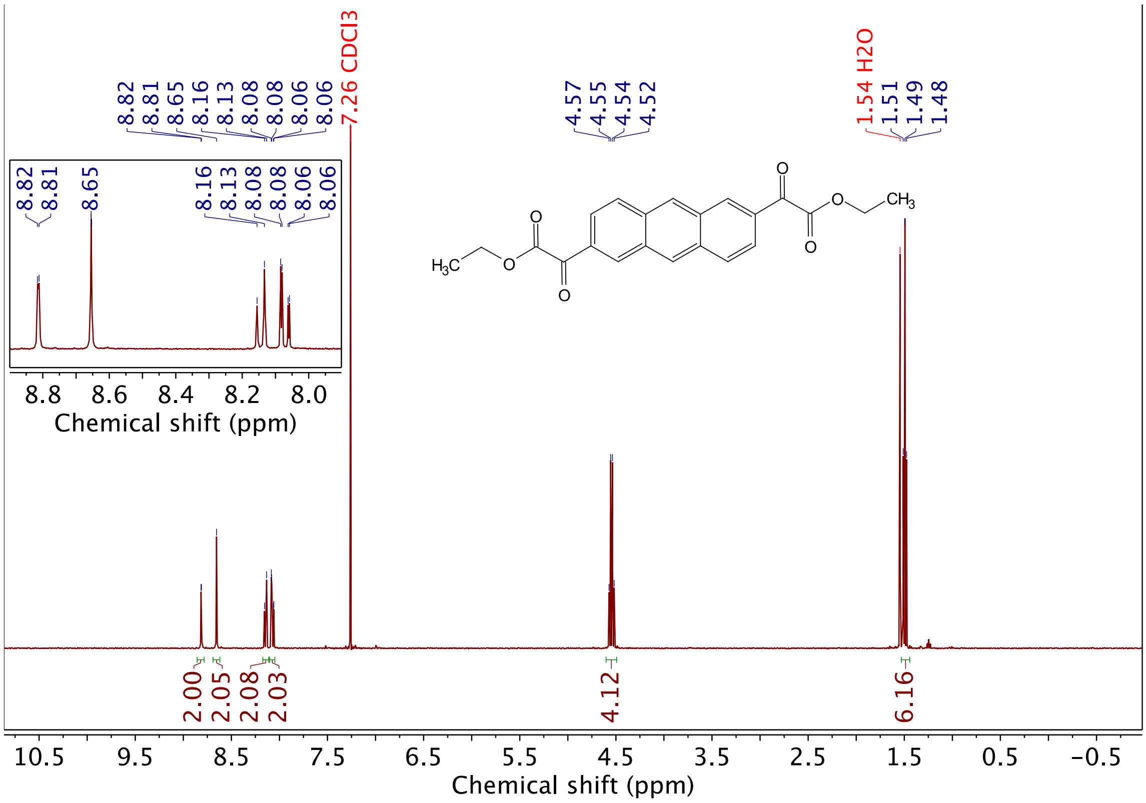


**Figure S7.** $^{1}$H NMR (400 MHz, $CDCl_3$) of compound **1c**.

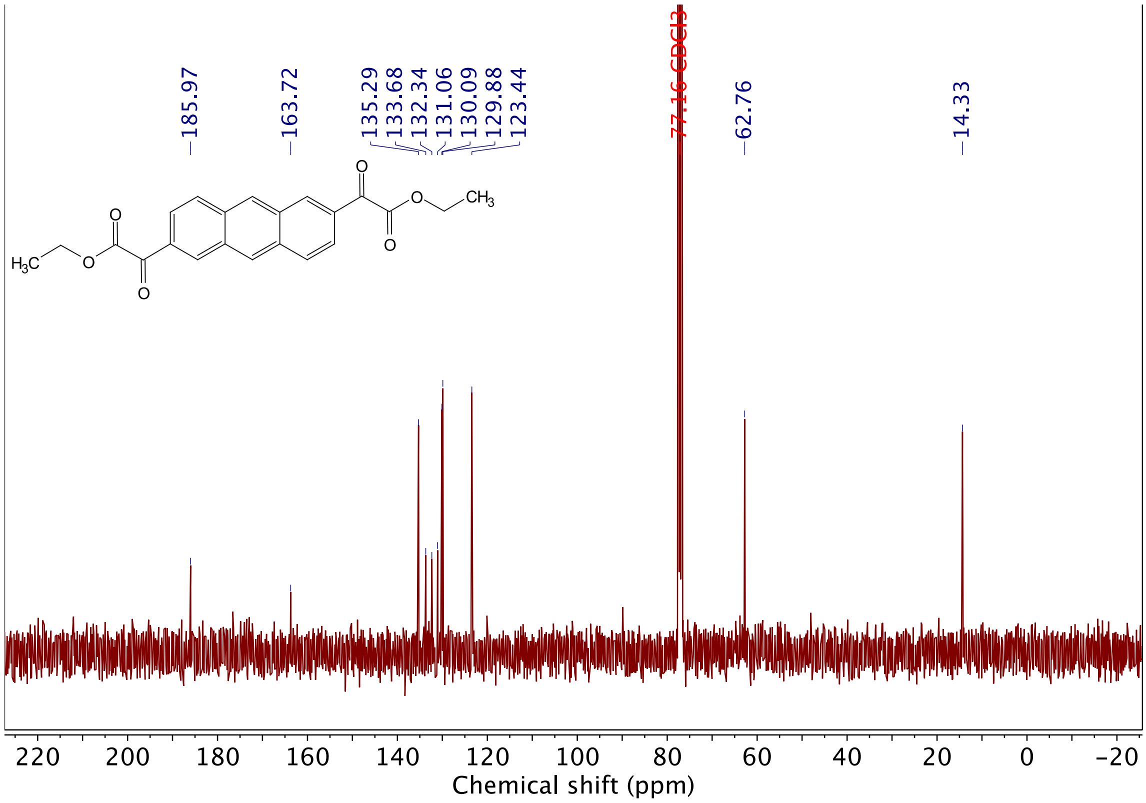


**Figure S8.** $^{13}$C NMR (101 MHz, $CDCl_3$) of compound **1c**.

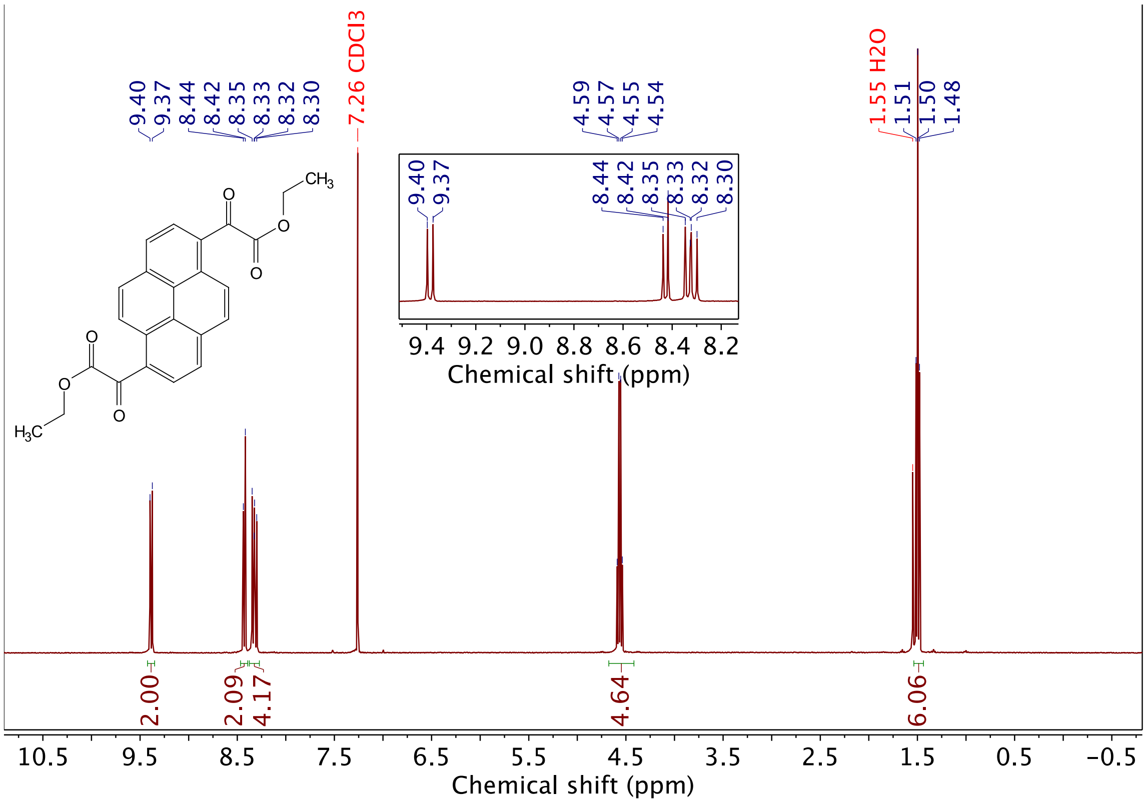


**Figure S9.** $^{1}H$ NMR (400 MHz, $CDCl_3$) of compound **1d**.

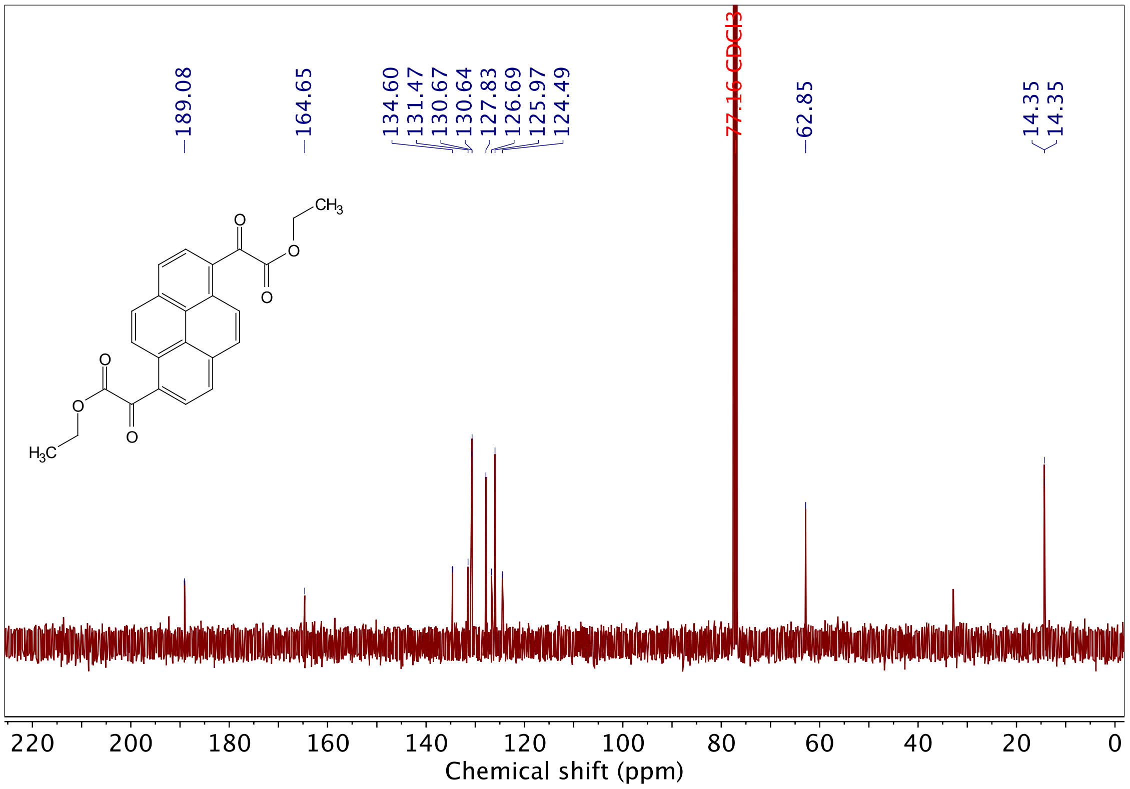


**Figure S10.** $^{13}C$ NMR (101 MHz, $CDCl_3$) of compound **1d**.

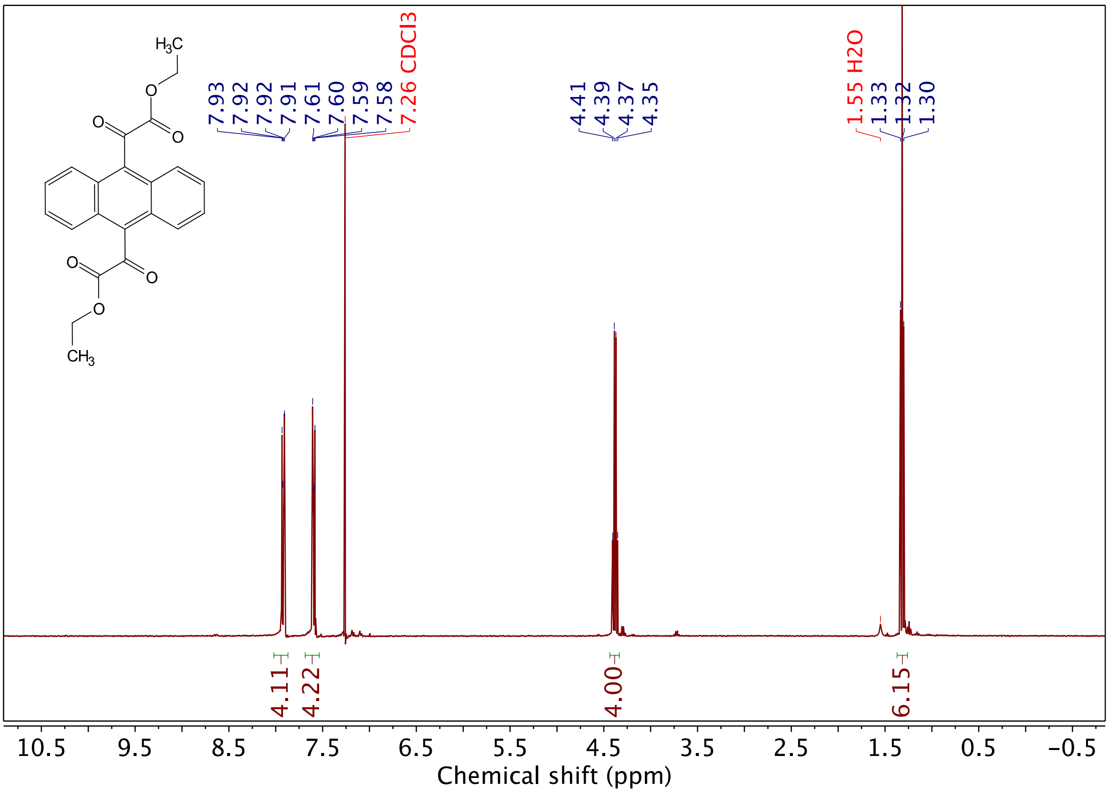


**Figure S11.** $^{1}$H NMR (400 MHz, $CDCl_3$) of compound **1e**.

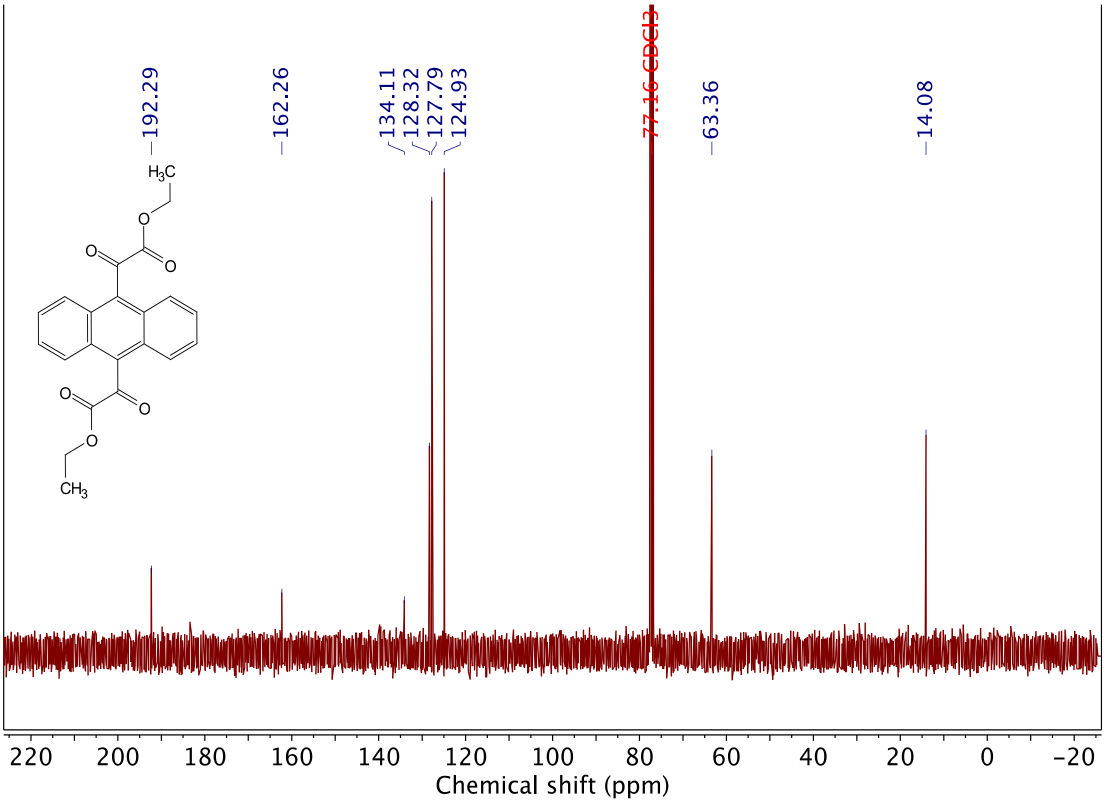


**Figure S12.** $^{13}$C NMR (101 MHz, $CDCl_3$) of compound **1e**.

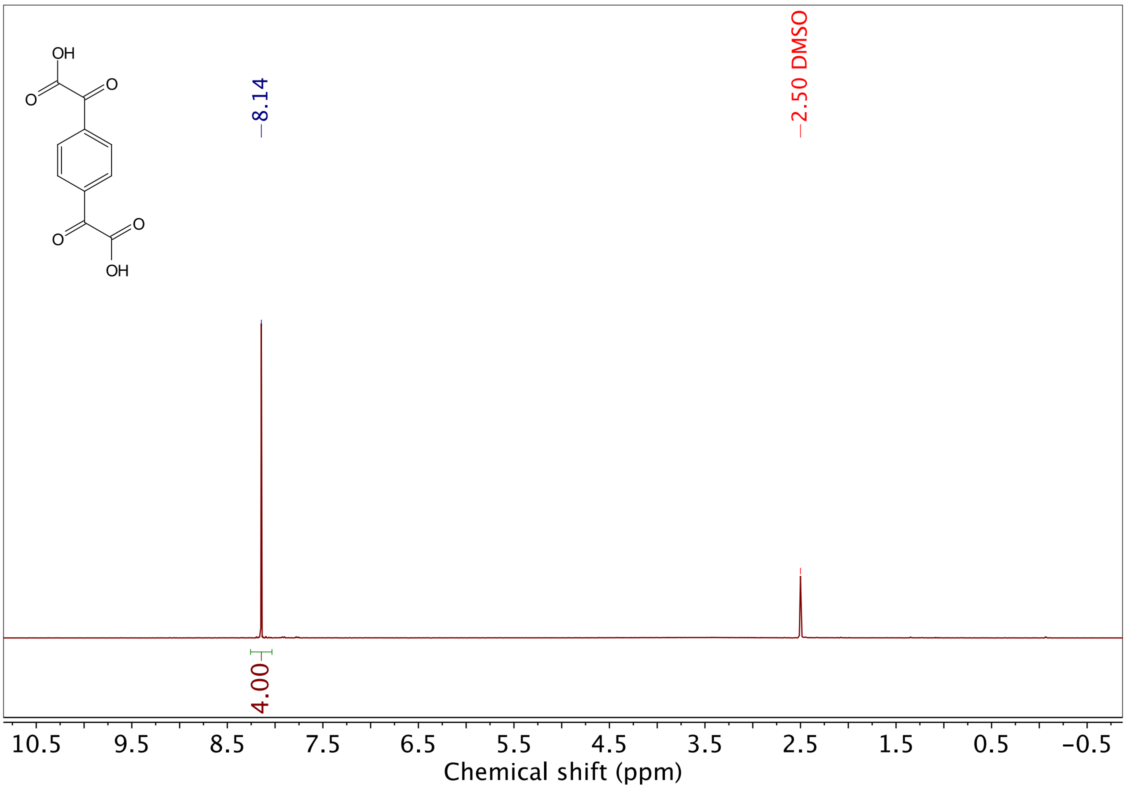


**Figure S13.** $^{1}H$ NMR (400 MHz, DMSO-*d6*) of compound **2a**.

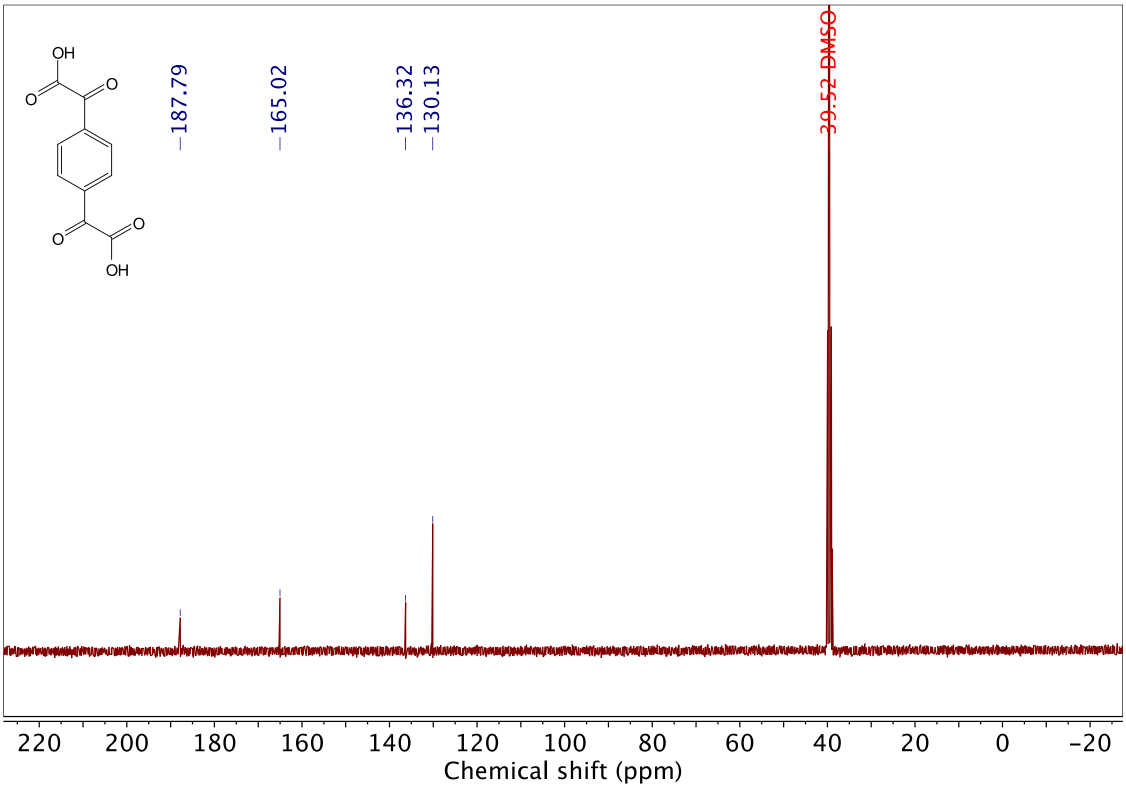


**Figure S14.** $^{13}C$ NMR (101 MHz, DMSO-*d6*) of compound **2a**.

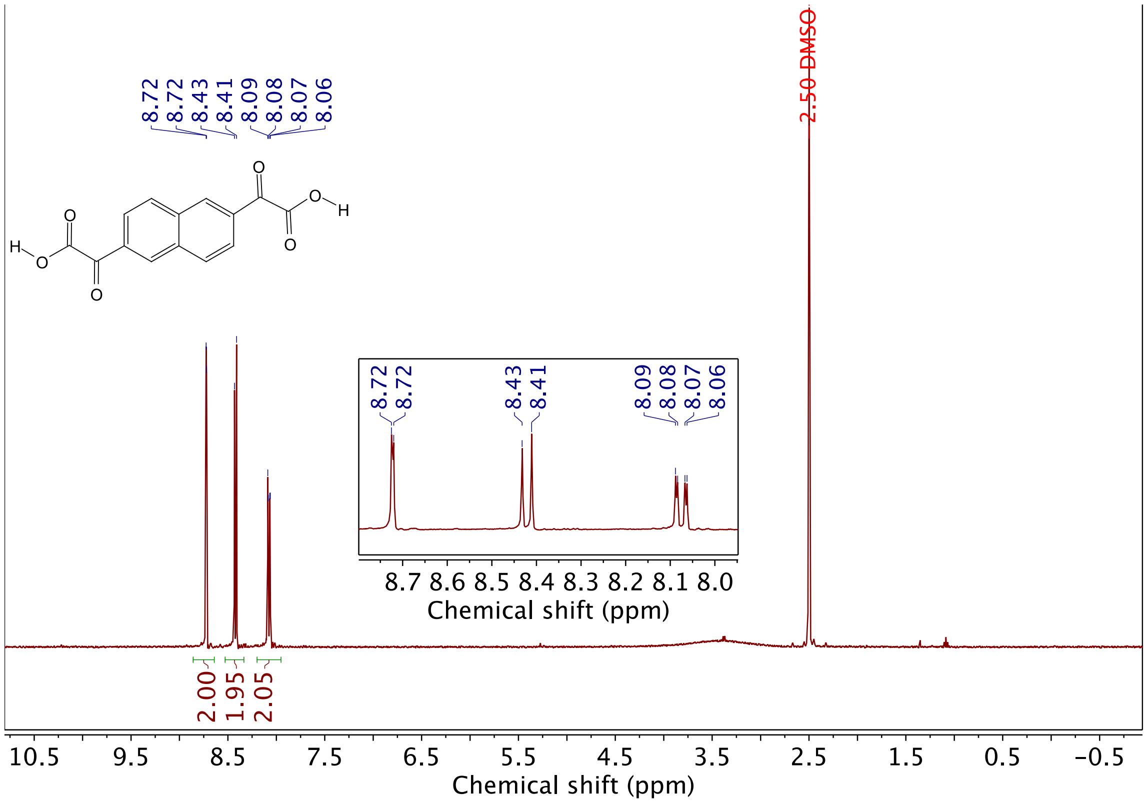


**Figure S15.** $^1$H NMR (400 MHz, DMSO-*d6*) of compound **2b**.

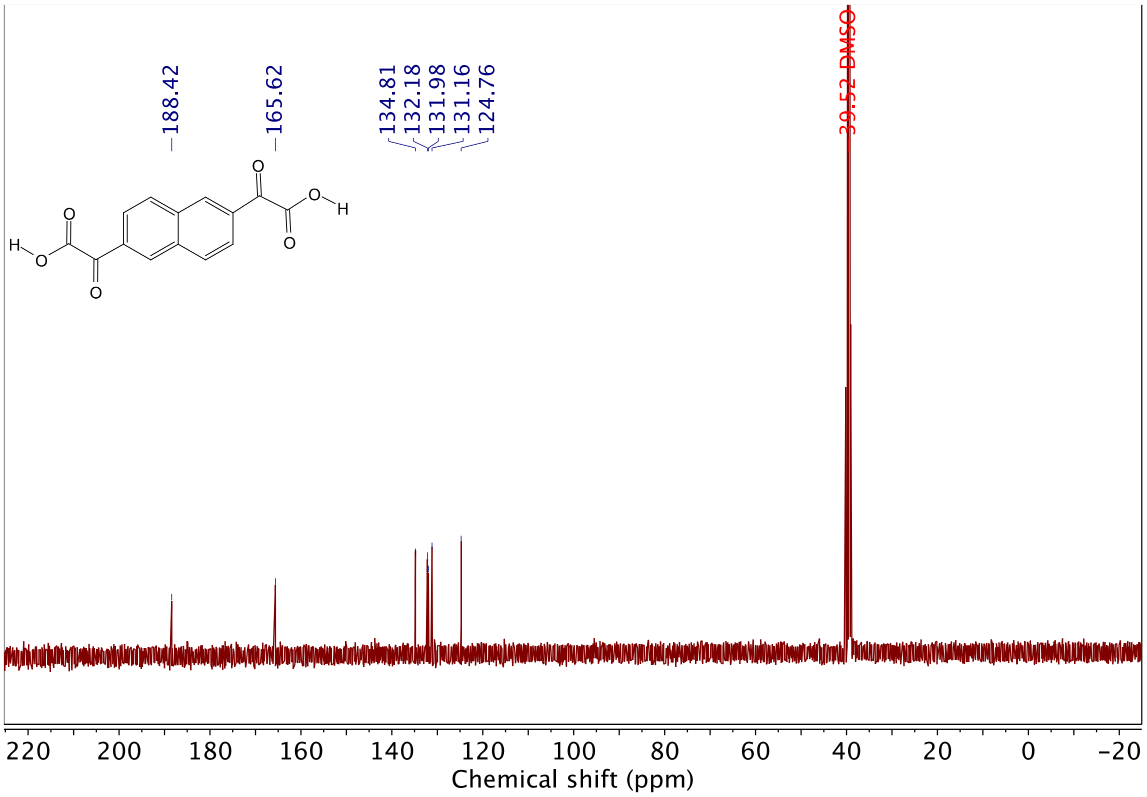


**Figure S16.** $^{13}$C NMR (101 MHz, DMSO-*d6*) of compound **2b**.

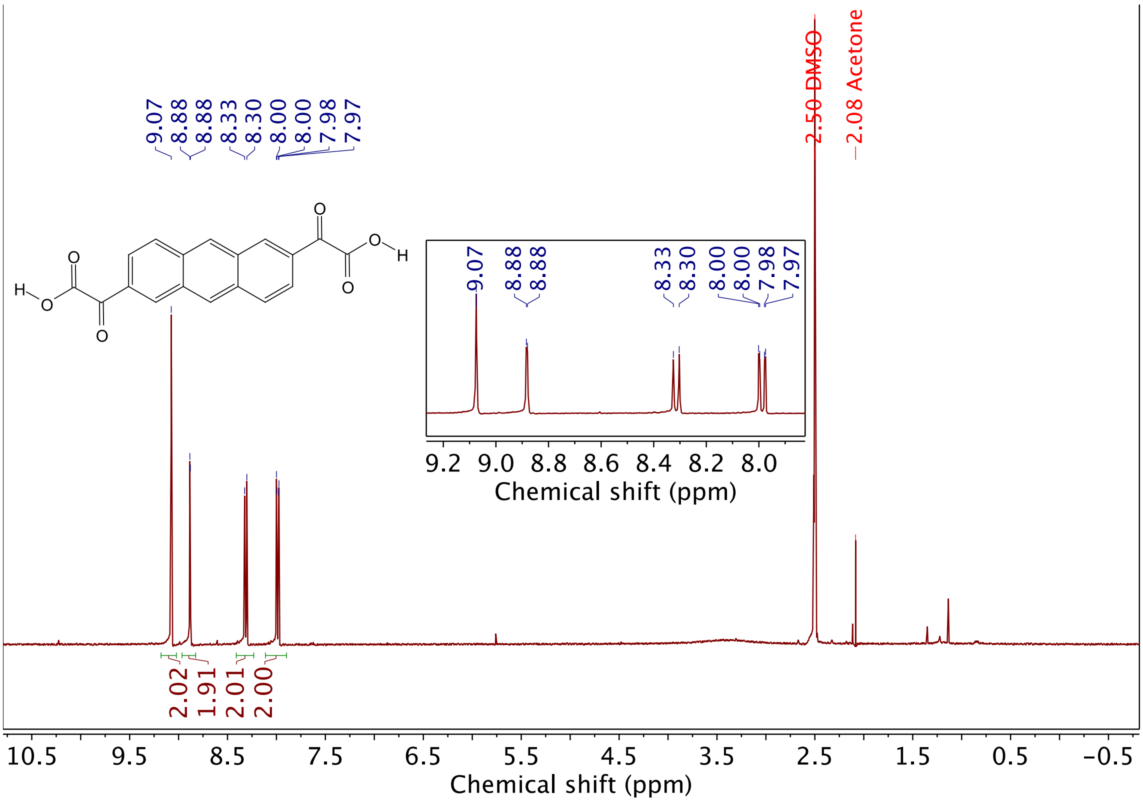


**Figure S17.** $^{1}$H NMR (400 MHz, DMSO-*d6*) of compound **2c**.

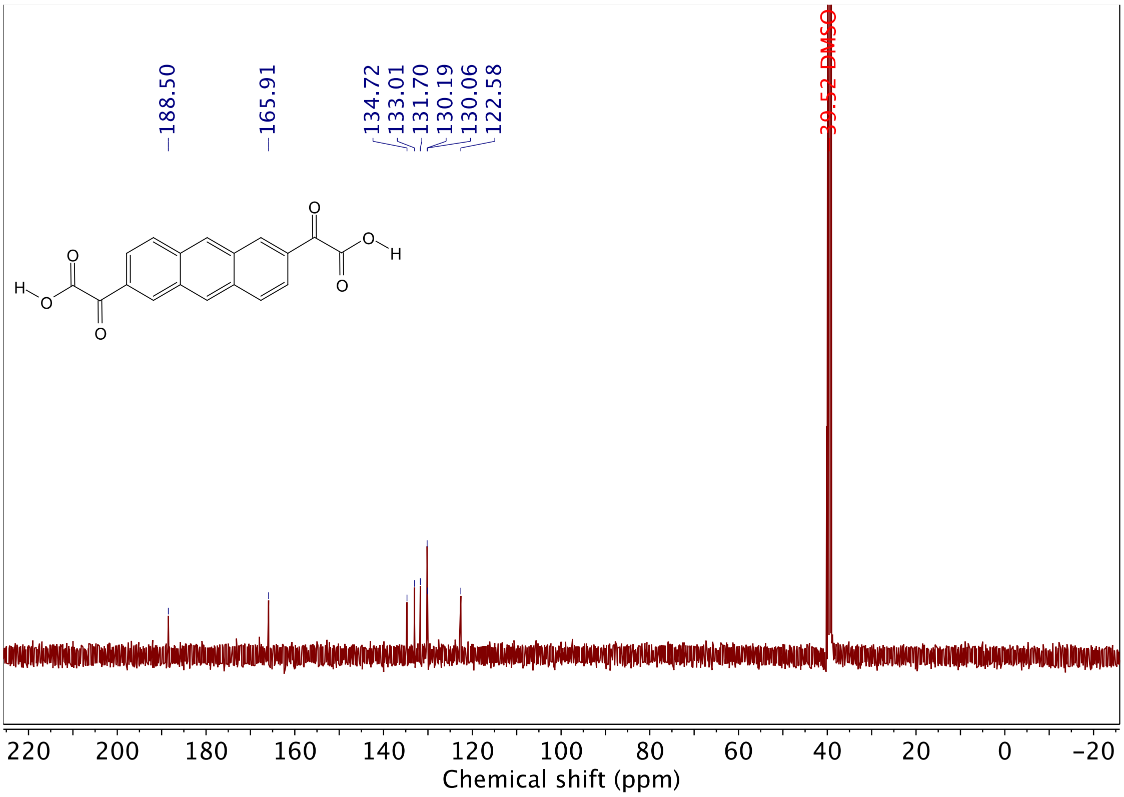


**Figure S18.** $^{13}$C NMR (101 MHz, DMSO-*d6*) of compound **2c**.

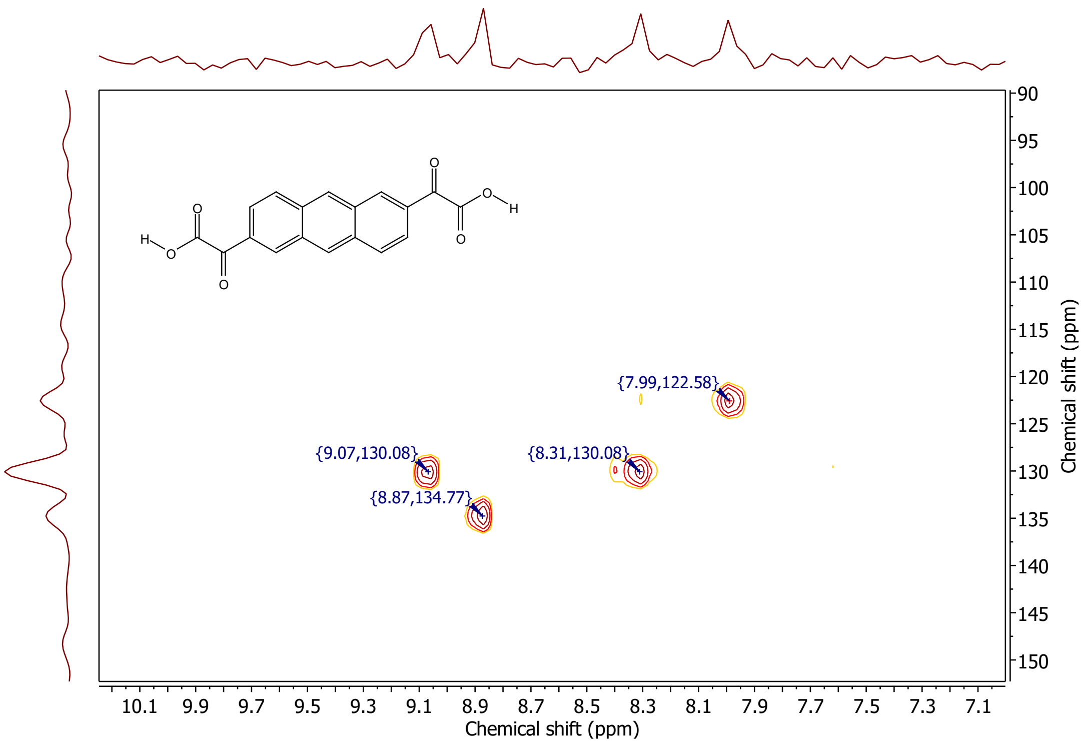


**Figure S19.** $^{1}H$-$^{13}C$ HSQC spectrum (400 MHz, 101 MHz, DMSO-*d6*) of compound **2c**.

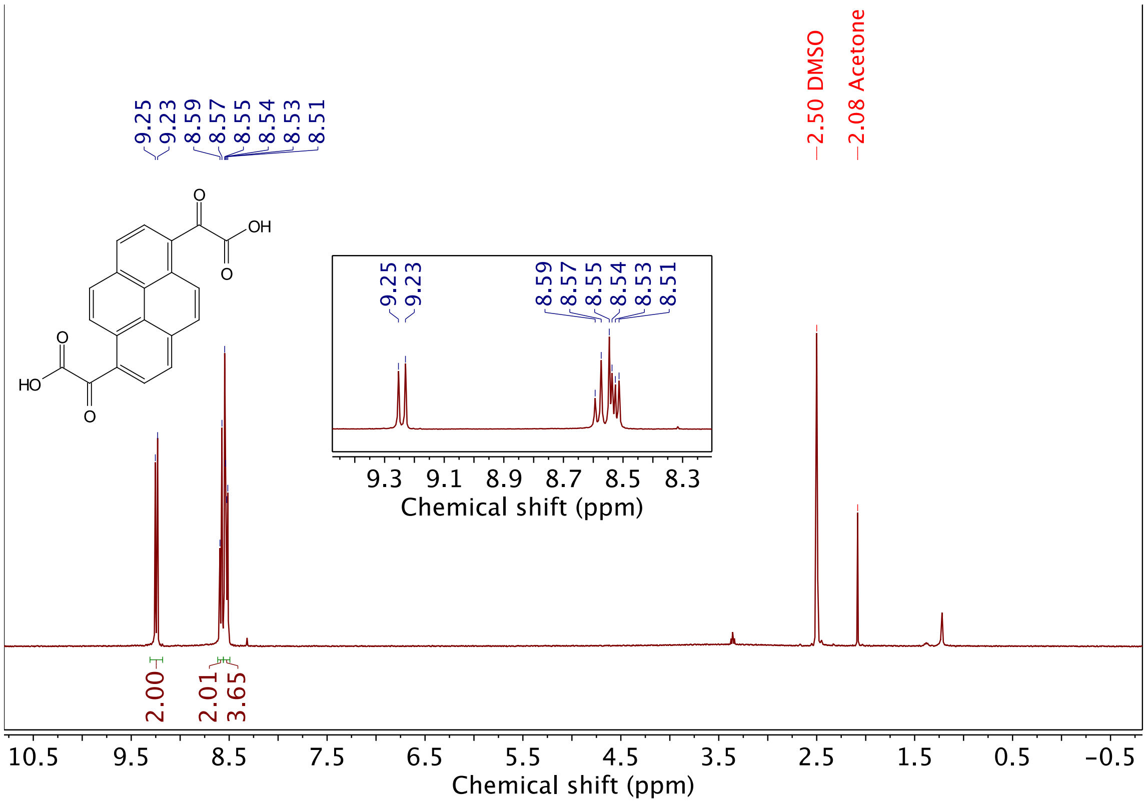


**Figure S20.** $^{1}H$ NMR (400 MHz, DMSO-*d6*) of compound **2d**.

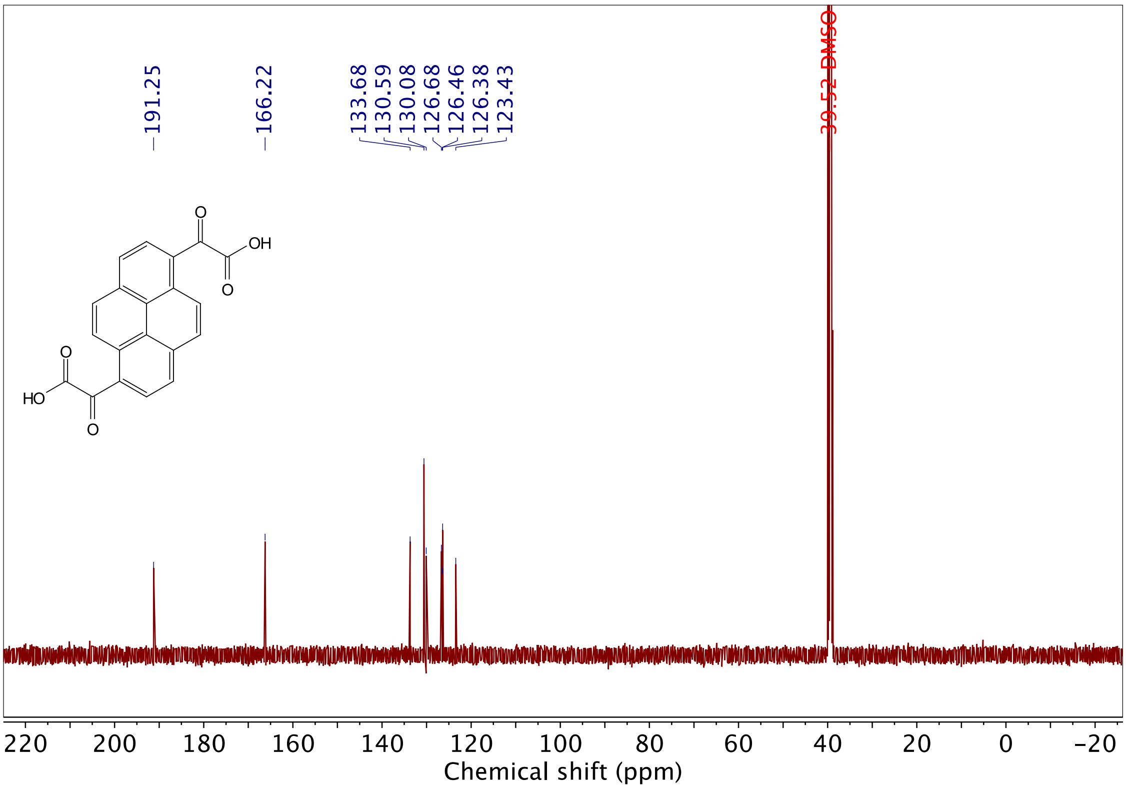


**Figure S21.** $^{13}$C NMR (101 MHz, DMSO-*d6*) of compound **2d**.

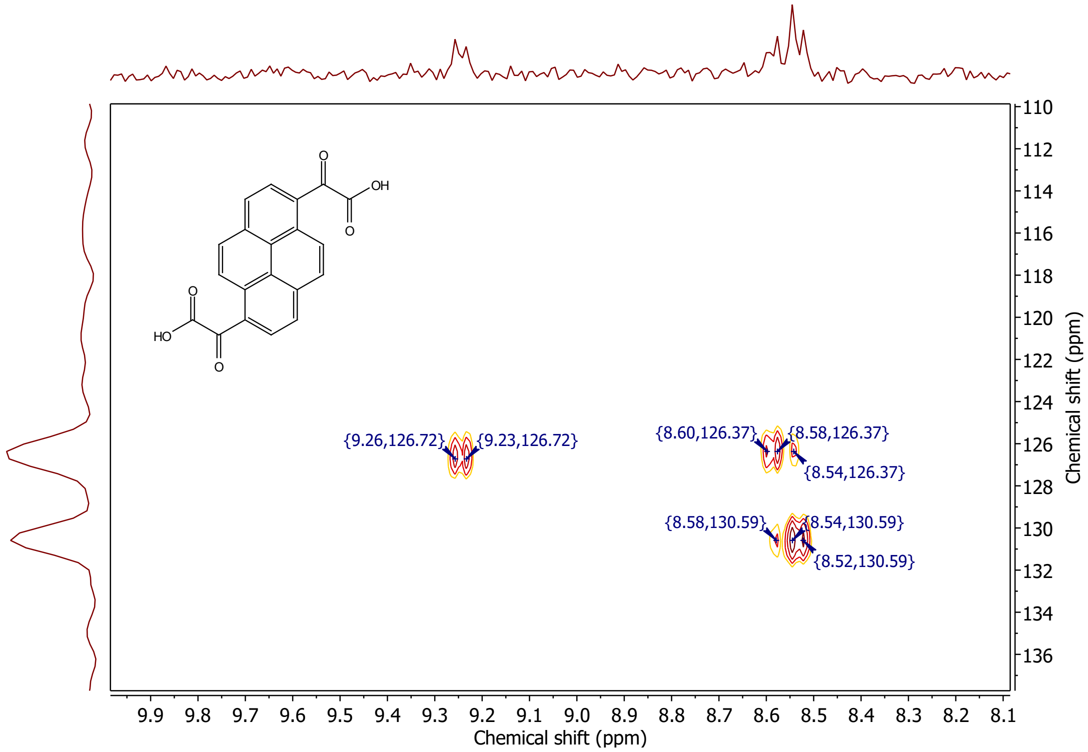


**Figure S22.** $^{1}$H-$^{13}$C HSQC spectrum (400 MHz, 101 MHz, DMSO-*d6*) of compound **2d**.

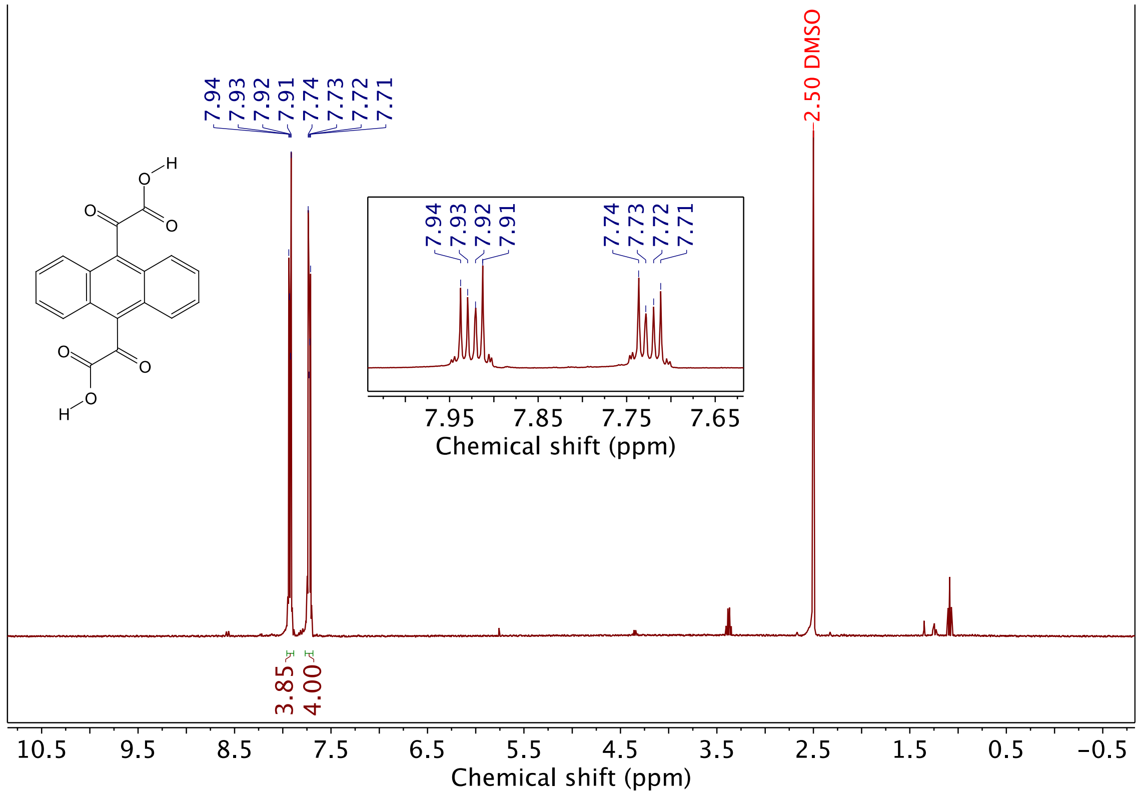


**Figure S23.** $^{1}$H NMR (400 MHz, DMSO-*d6*) of compound **2e**.

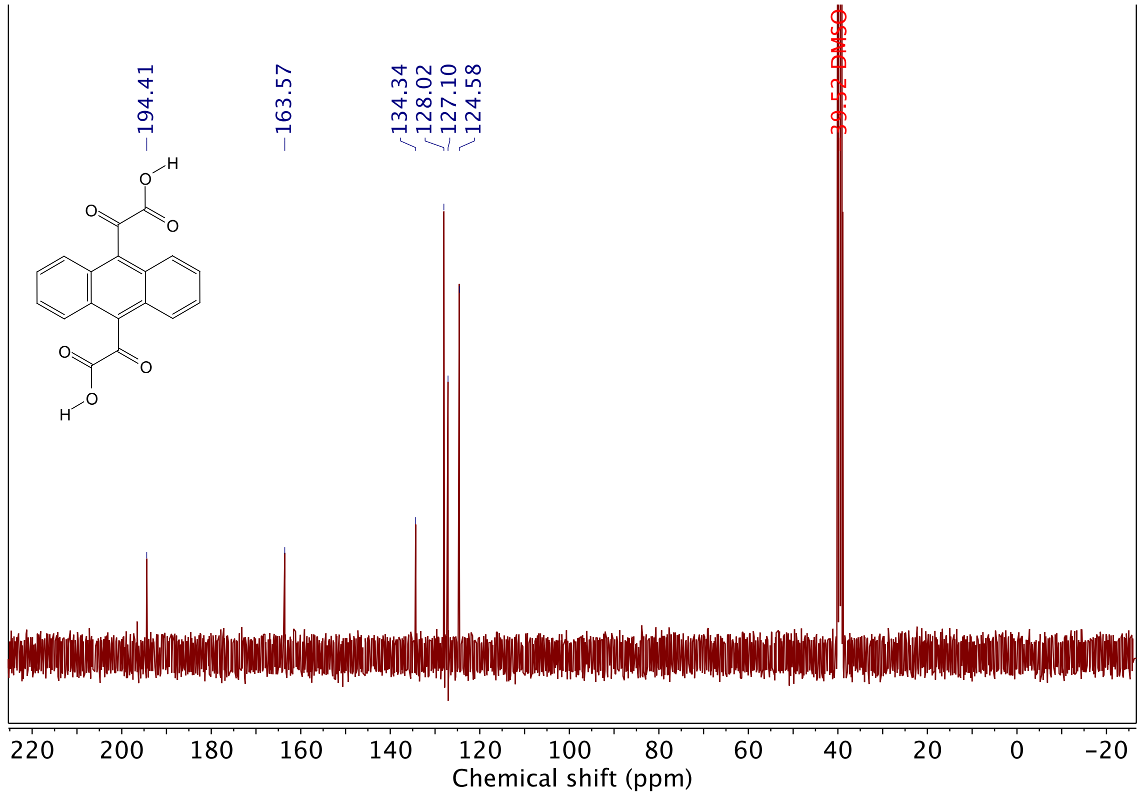


**Figure S24.** $^{13}$C NMR (101 MHz, DMSO-*d6*) of compound **2e**.

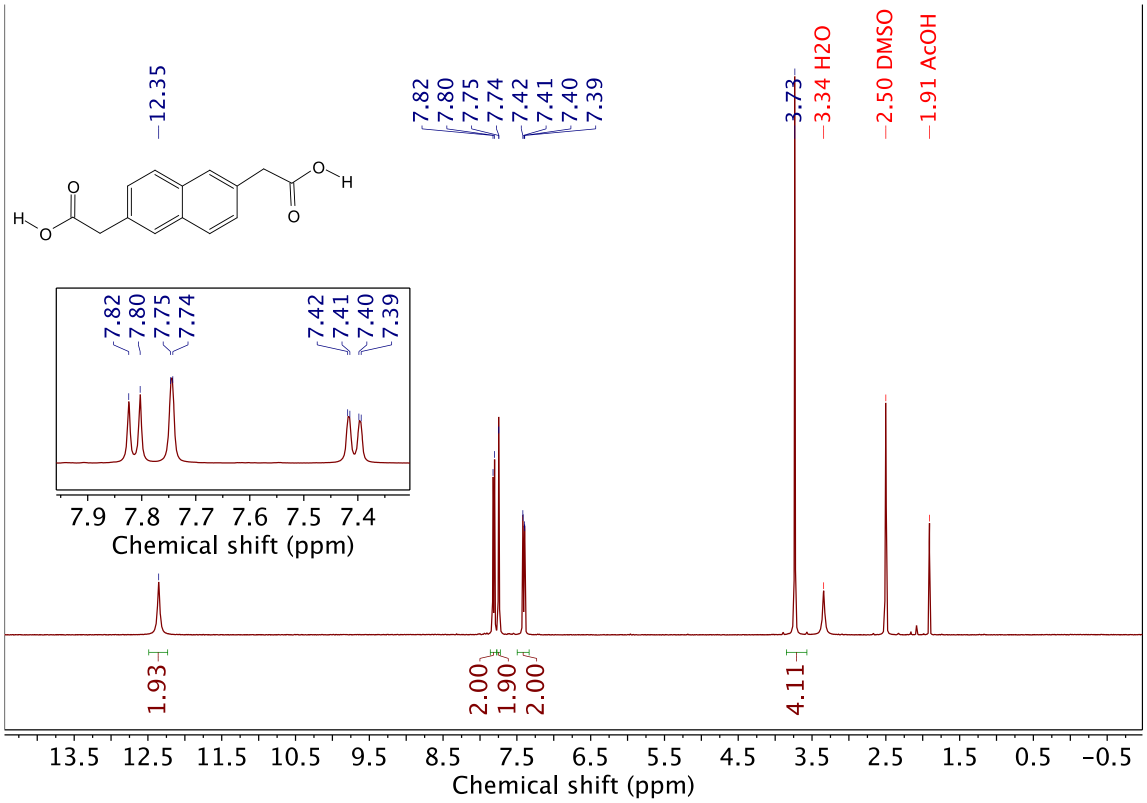

**Figure S25.** $^{1}$H NMR (400 MHz, DMSO-*d6*) of compound **3b**.

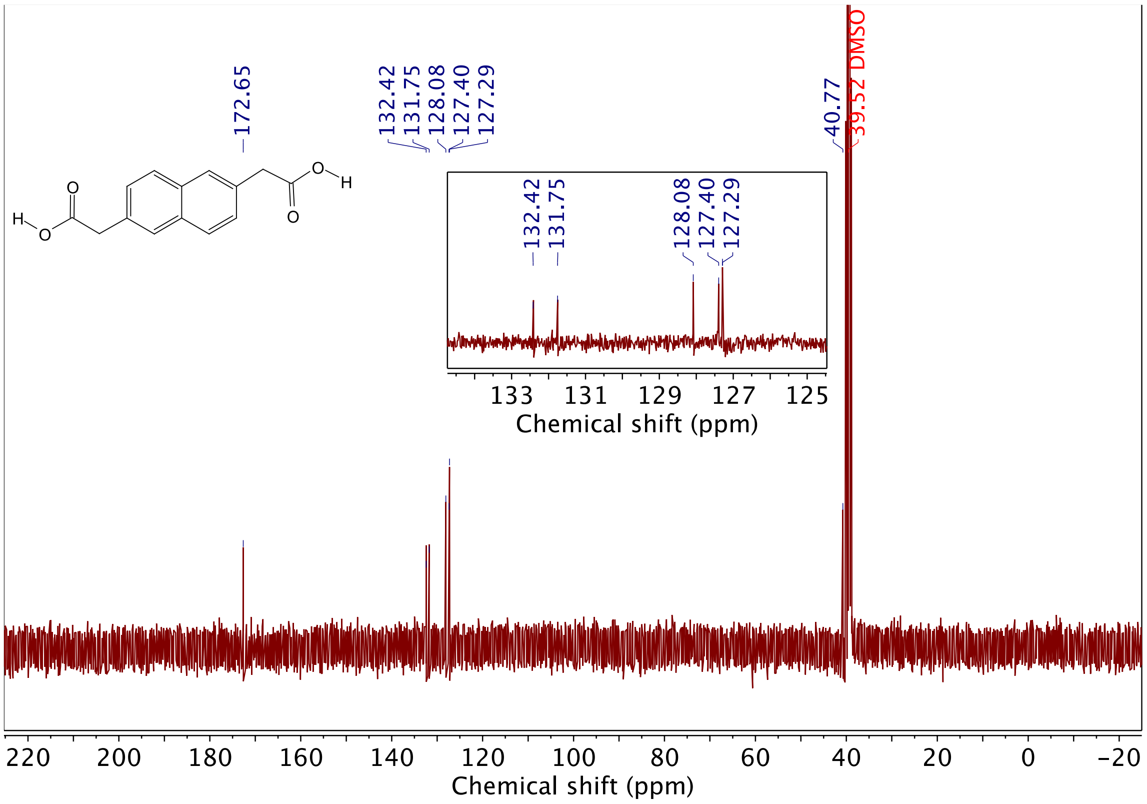

**Figure S26.** $^{13}$C NMR (101 MHz, DMSO-*d6*) of compound **3b**.

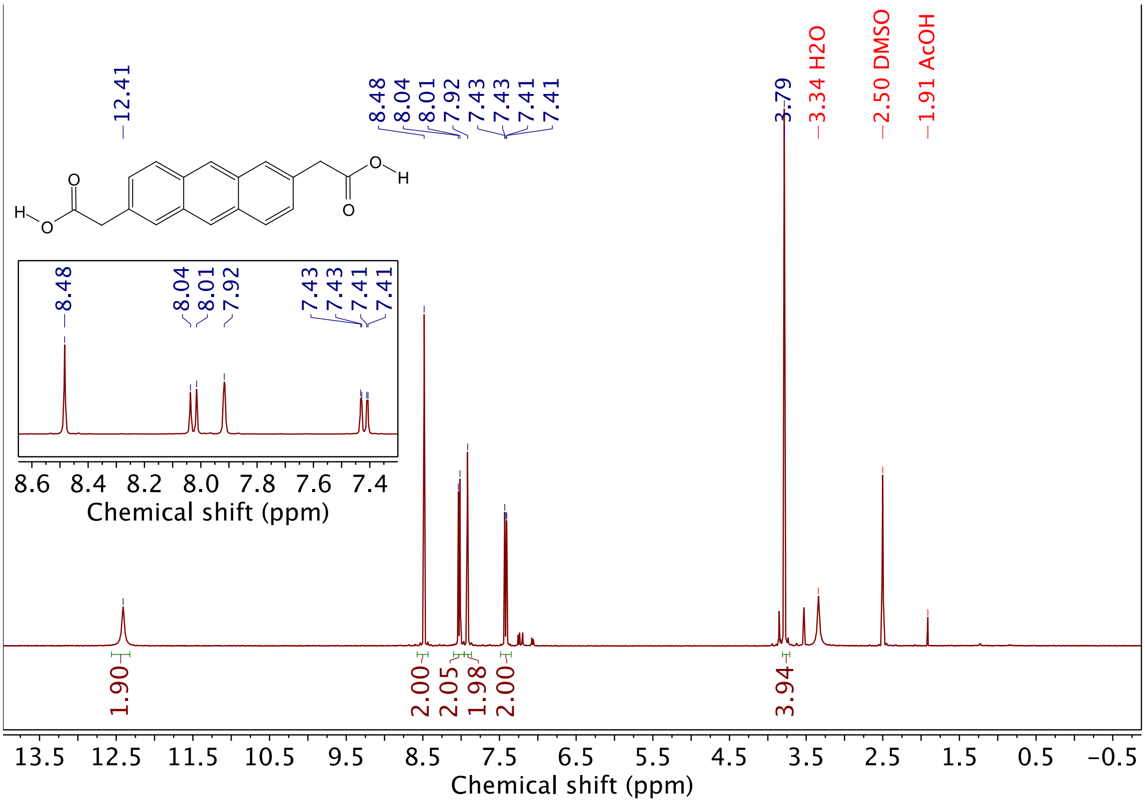


**Figure S27.** $^{1}$H NMR (400 MHz, DMSO-*d6*) of compound **3c**.

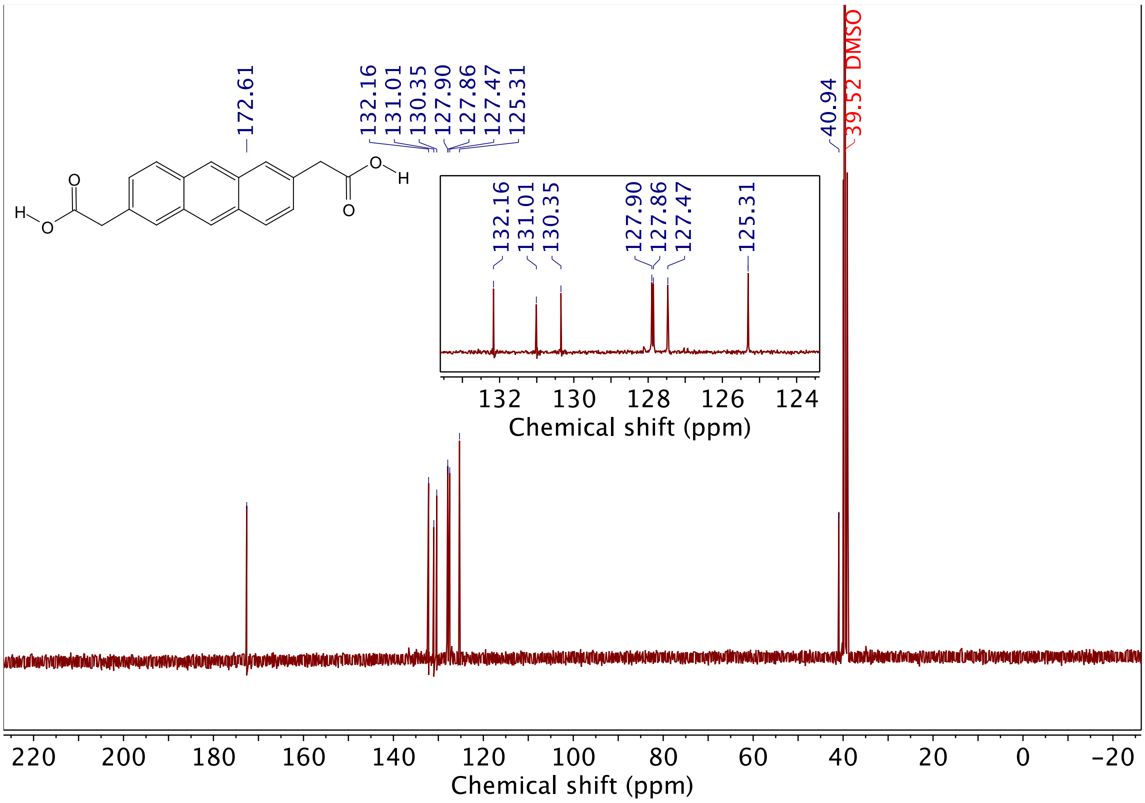


**Figure S28.** $^{13}$C NMR (101 MHz, DMSO-*d6*) of compound **3c**.

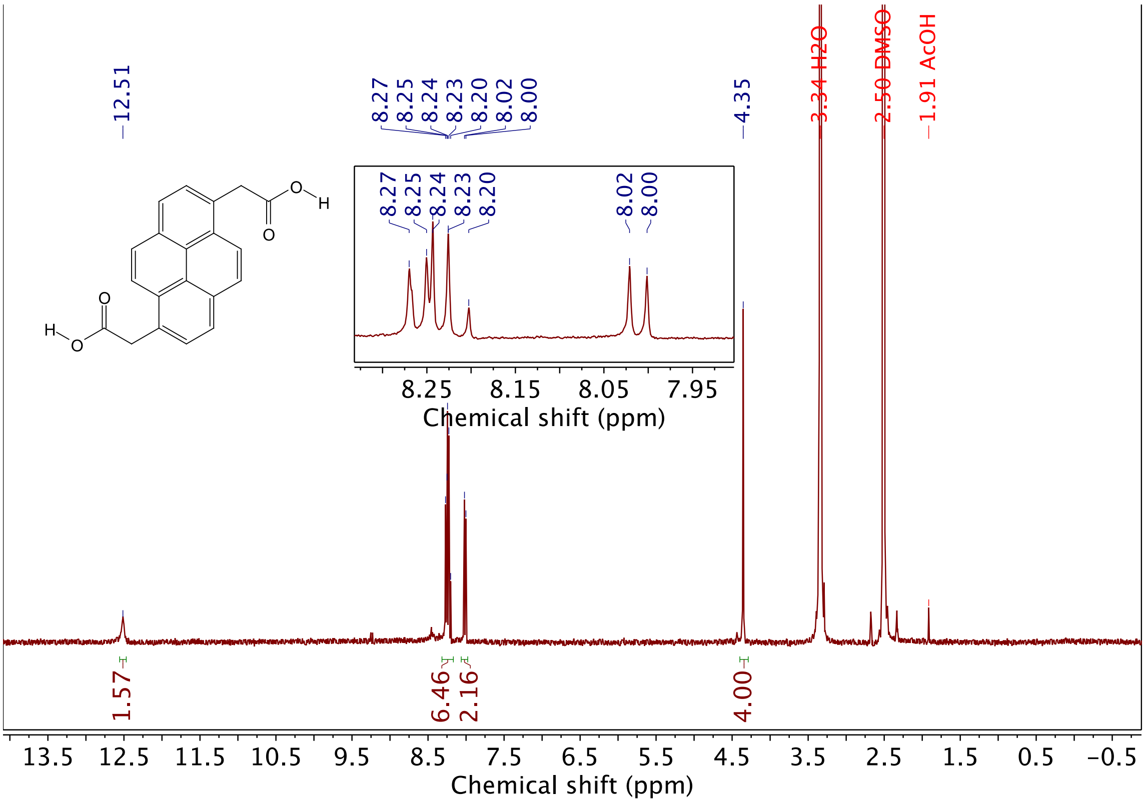


**Figure S29.** $^{1}H$ NMR (400 MHz, DMSO-*d6*) of compound **3d**

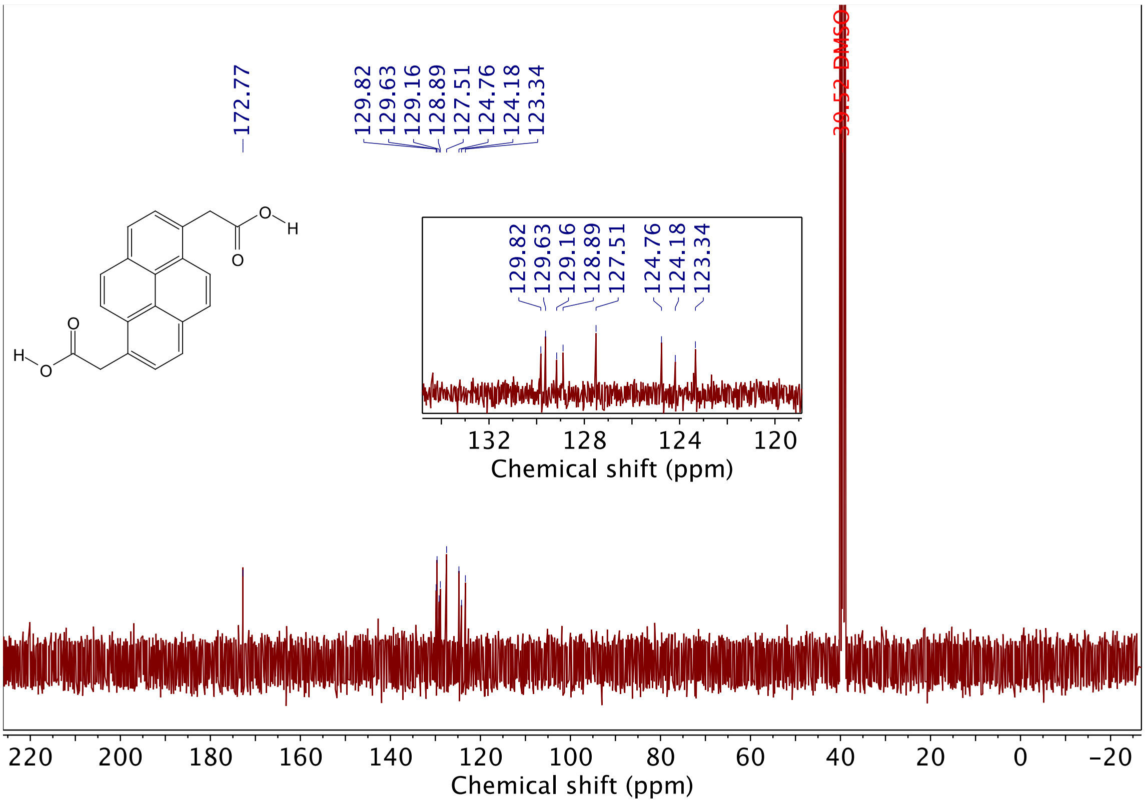


**Figure S30.** $^{13}C$ NMR (101 MHz, DMSO-*d6*) of compound **3d**.

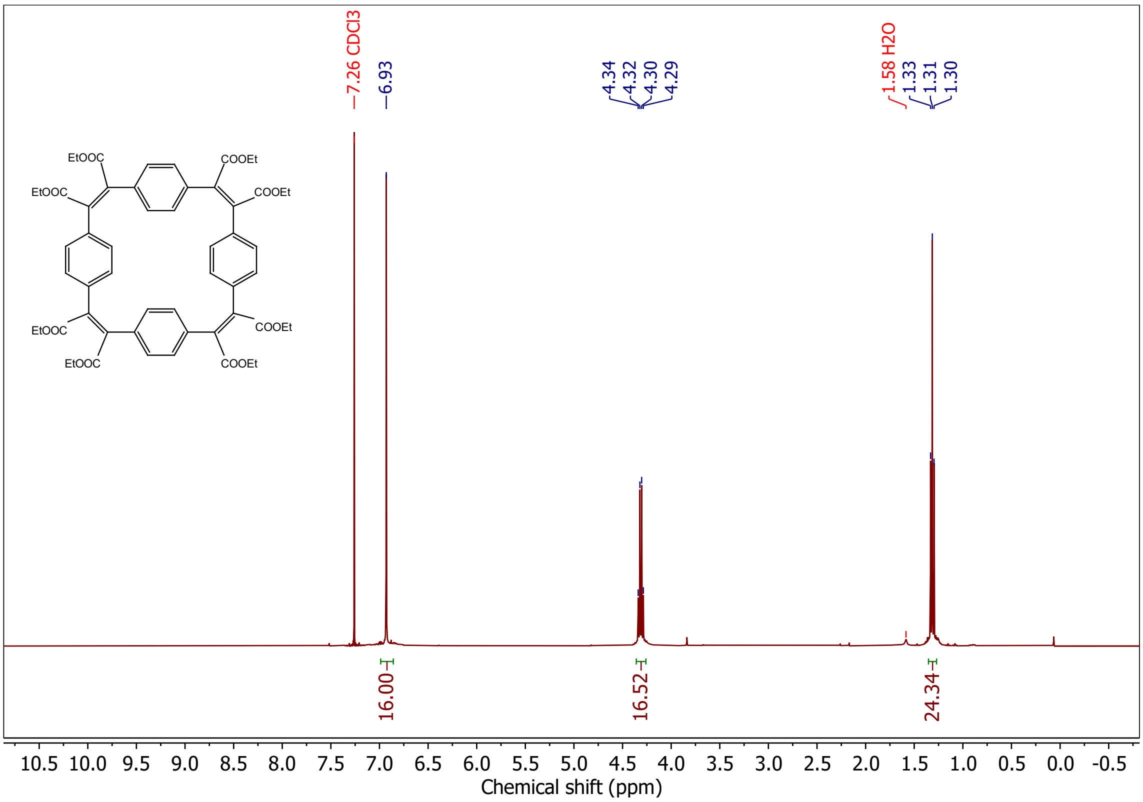


**Figure S31.** $^{1}$H NMR (400 MHz, $CDCl_3$) of macrocycle **BCyc-Et**.

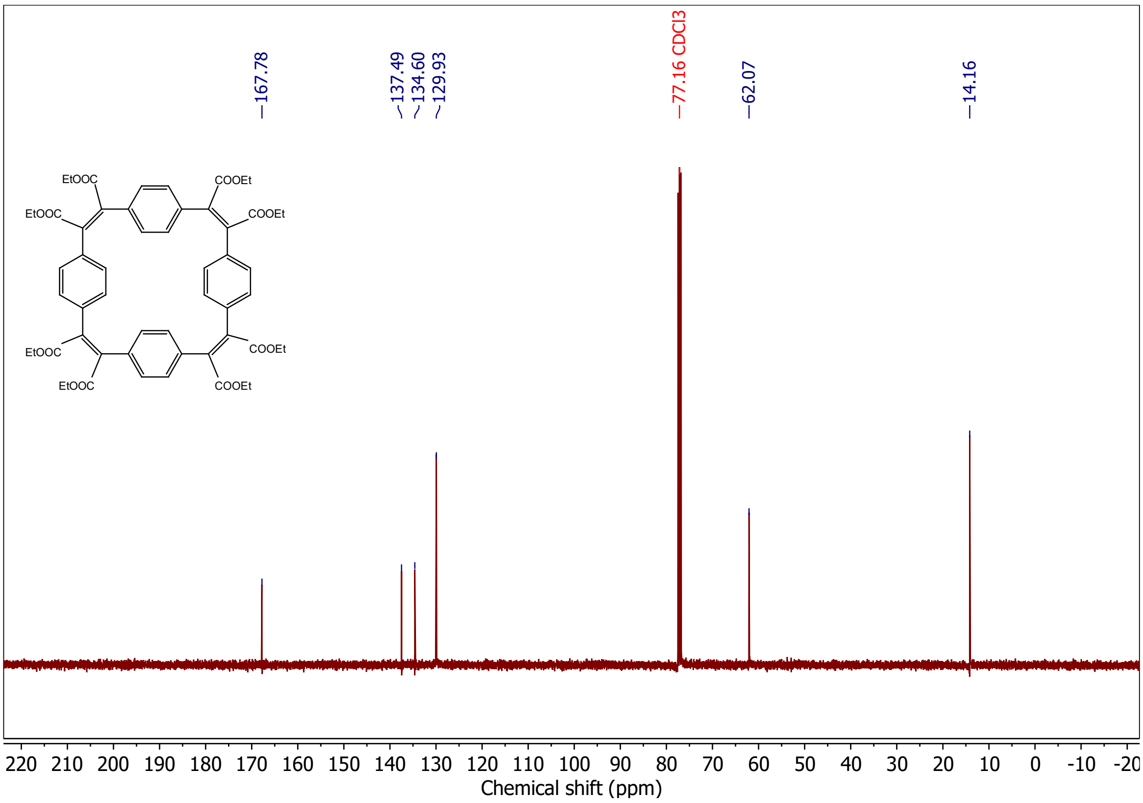


**Figure S32.** $^{13}$C NMR (101 MHz, $CDCl_3$) of macrocycle **BCyc-Et**.

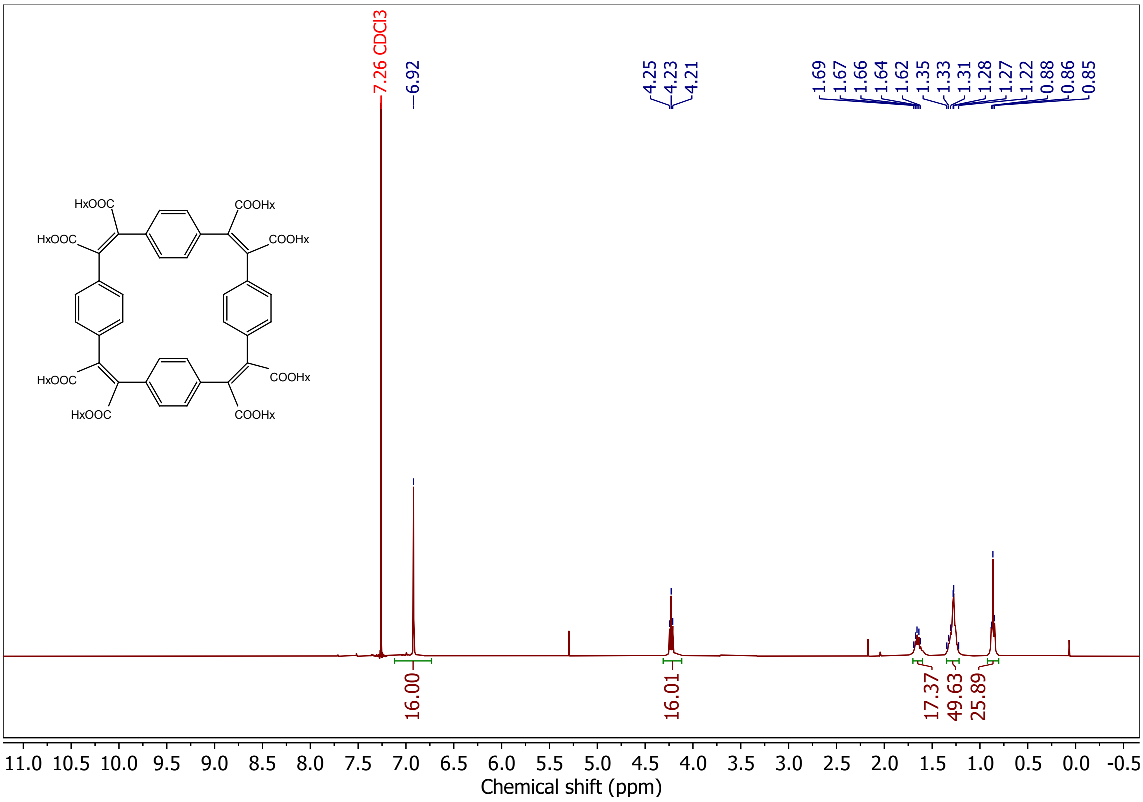


**Figure S33.** $^{1}H$ NMR (400 MHz, $CDCl_3$) of macrocycle **BCyc-Hx**.

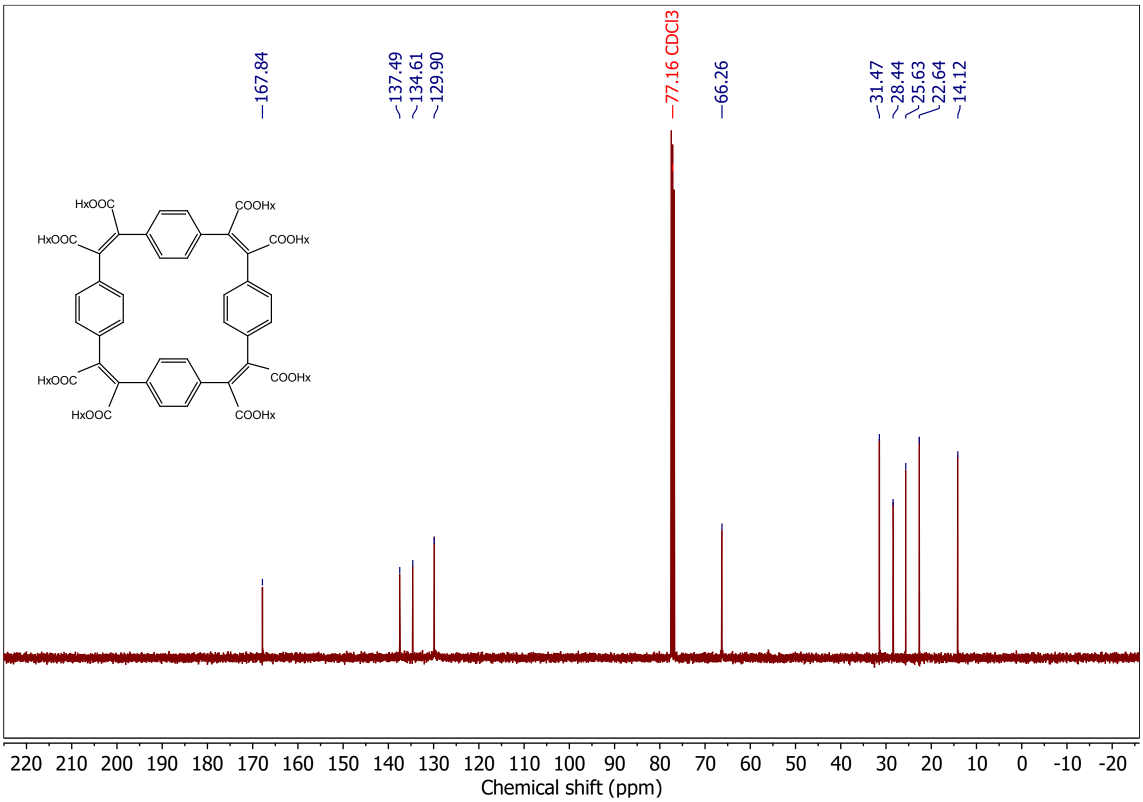


**Figure S34.** $^{13}C$ NMR (101 MHz, $CDCl_3$) of macrocycle **BCyc-Hx**.

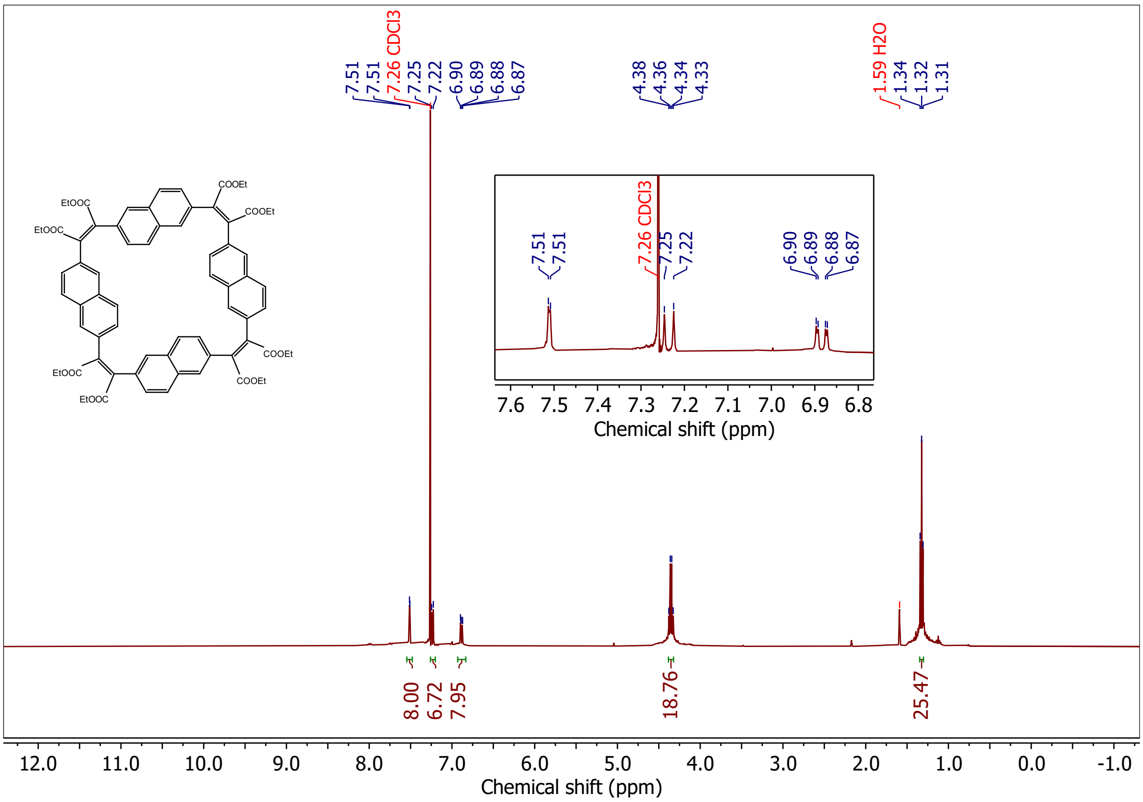


**Figure S35.** $^{1}H$ NMR (400 MHz, $CDCl_3$) of macrocycle **NCyc-Et**.

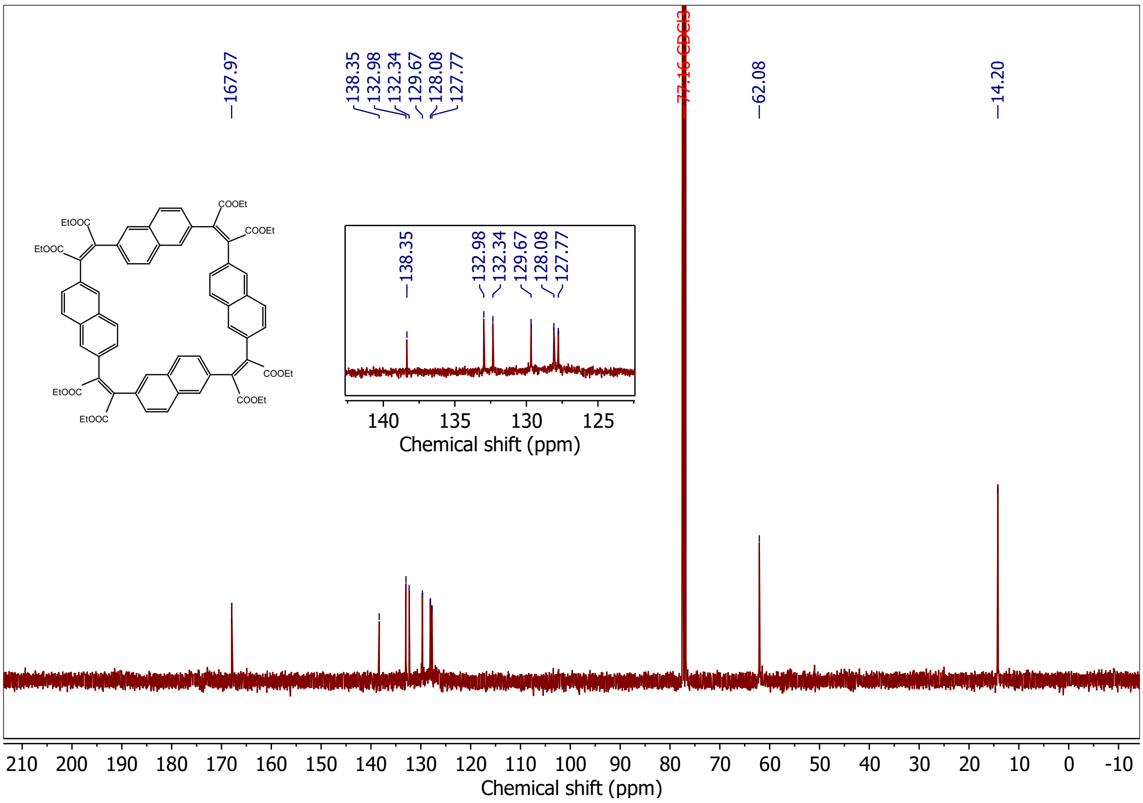


**Figure S36.** $^{13}C$ NMR (101 MHz, $CDCl_3$) of macrocycle **NCyc-Et**.

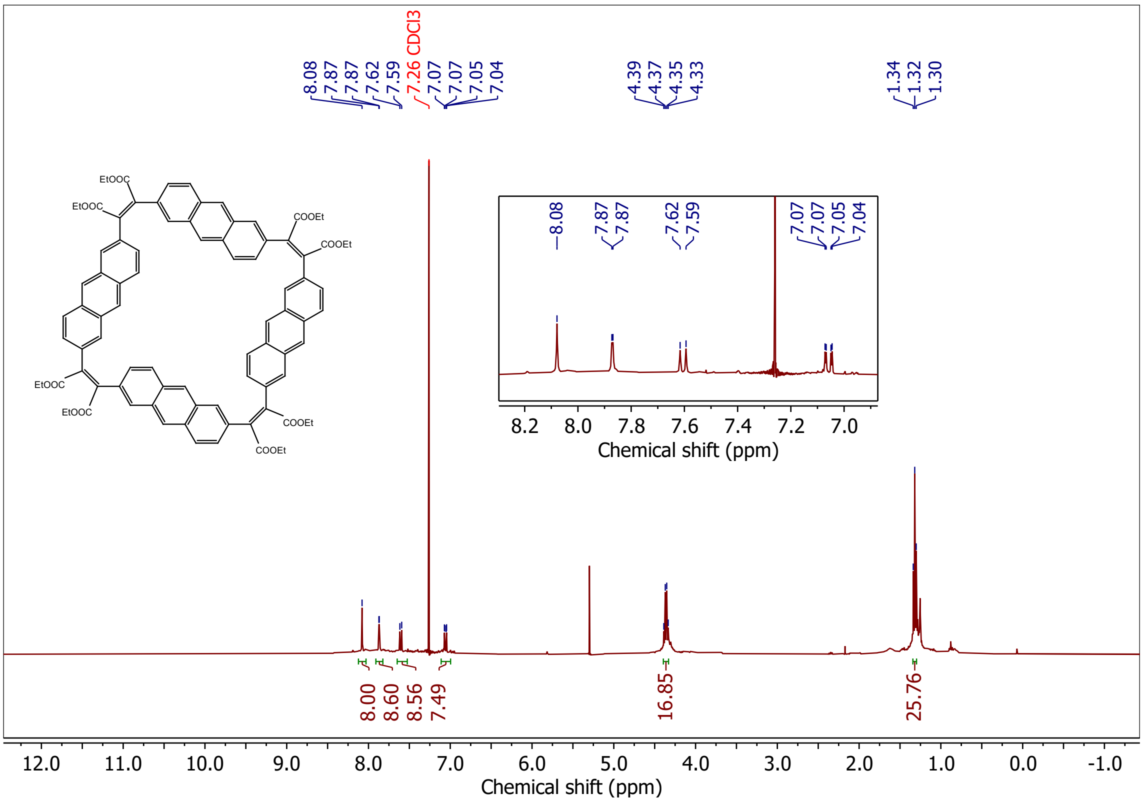


**Figure S37.** $^{1}$H NMR (400 MHz, $CDCl_3$) of macrocycle **ACyc-Et**.

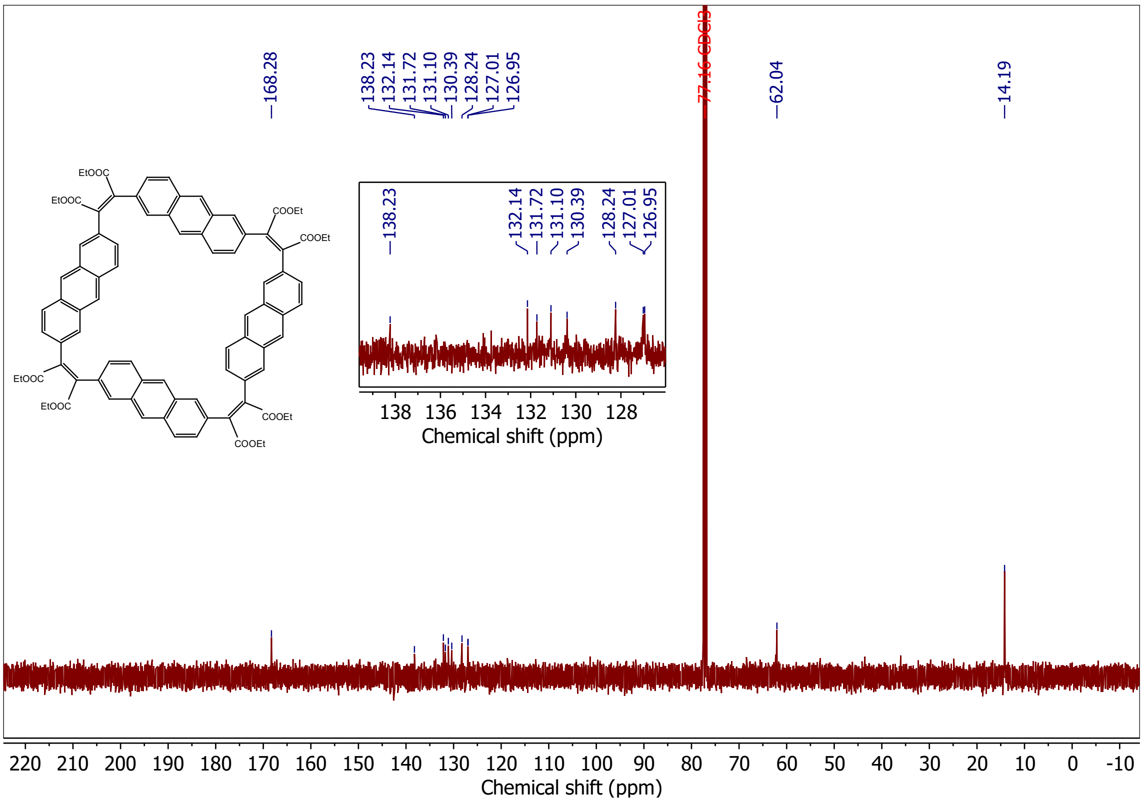


**Figure S38.** $^{13}$C NMR (101 MHz, $CDCl_3$) of macrocycle **ACyc-Et**.

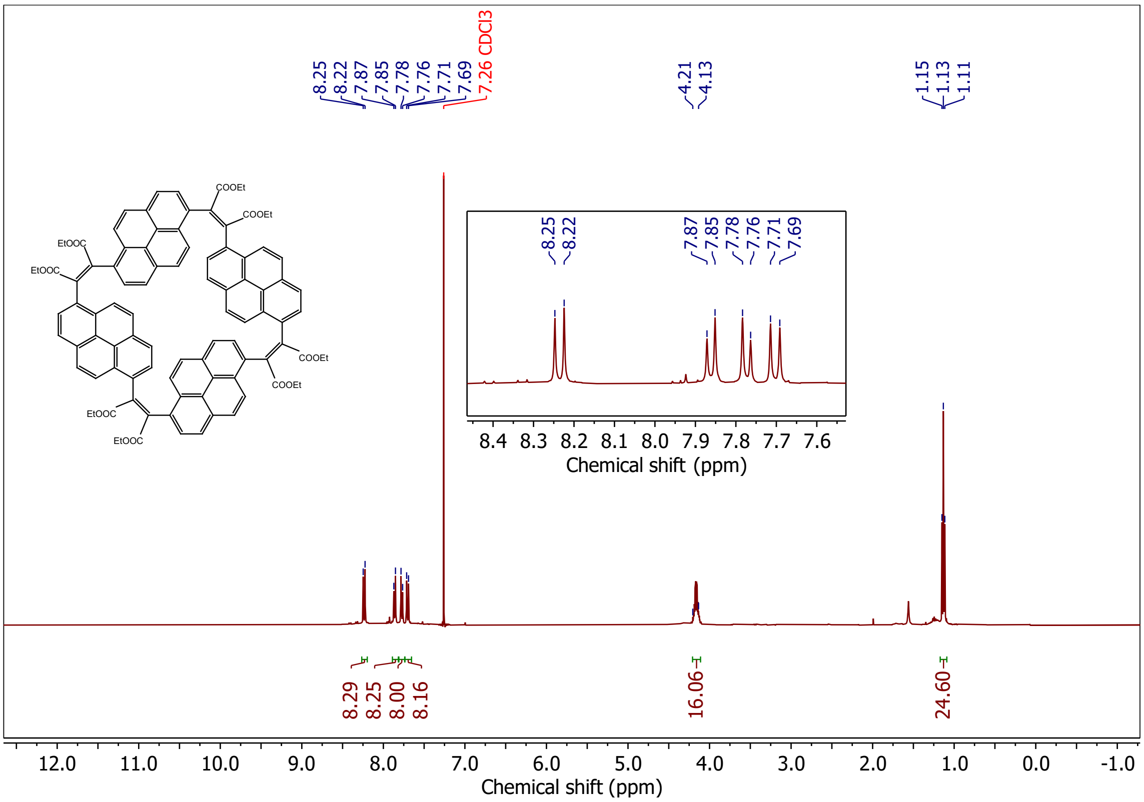


**Figure S39.** $^{1}H$ NMR (400 MHz, $CDCl_3$) of macrocycle **PCyc-Et**.

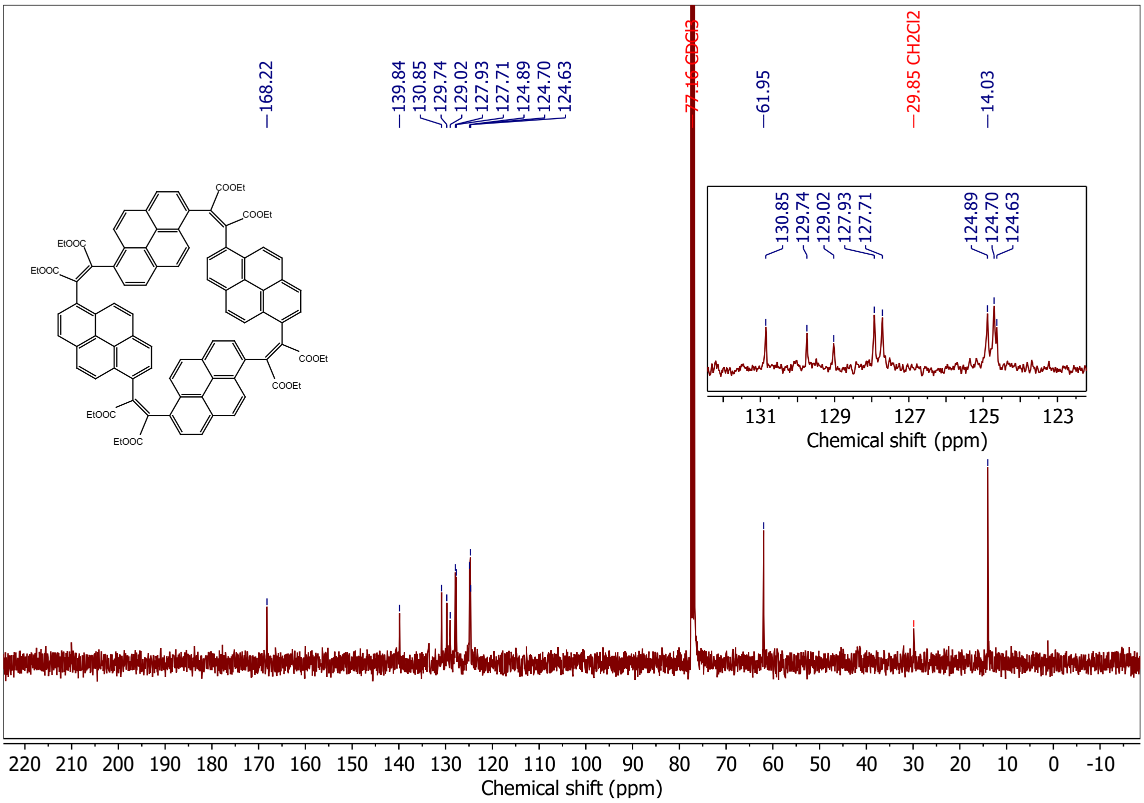


**Figure S40.** $^{13}C$ NMR (101 MHz, $CDCl_3$) of macrocycle **PCyc-Et**.

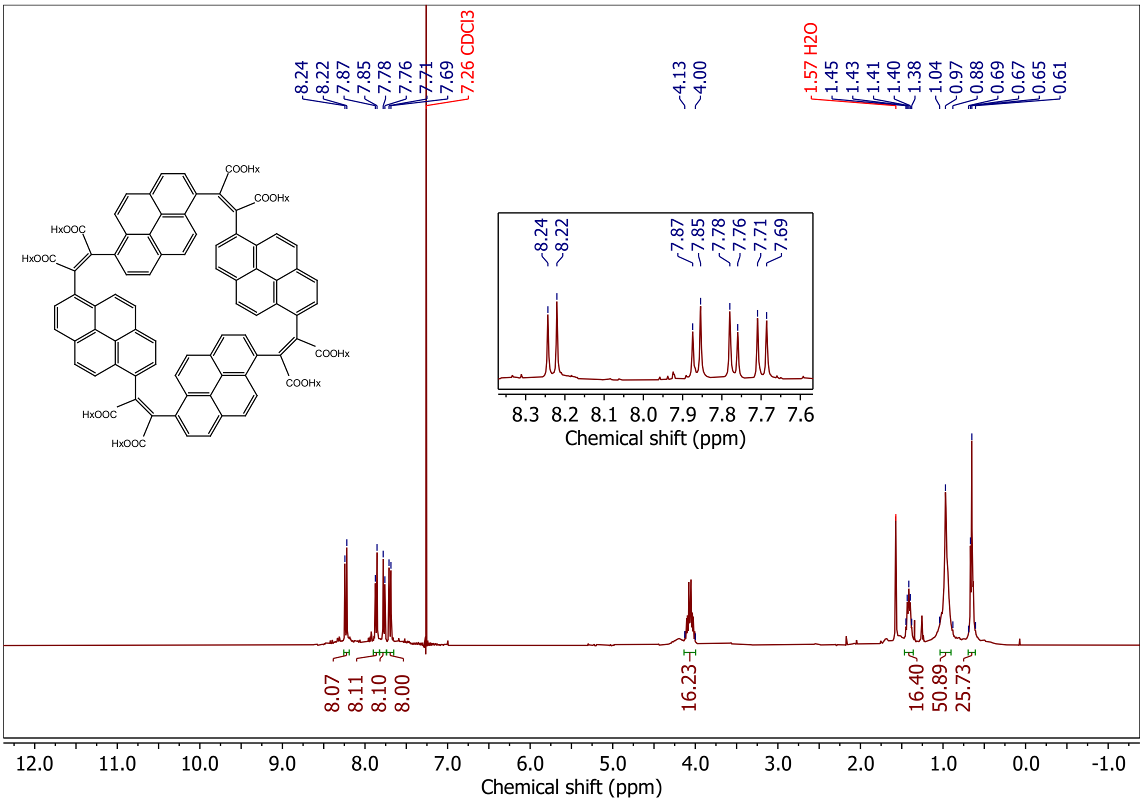

**Figure S41.** $^{1}$H NMR (400 MHz, $CDCl_3$) of macrocycle **PCyc-Hx**.

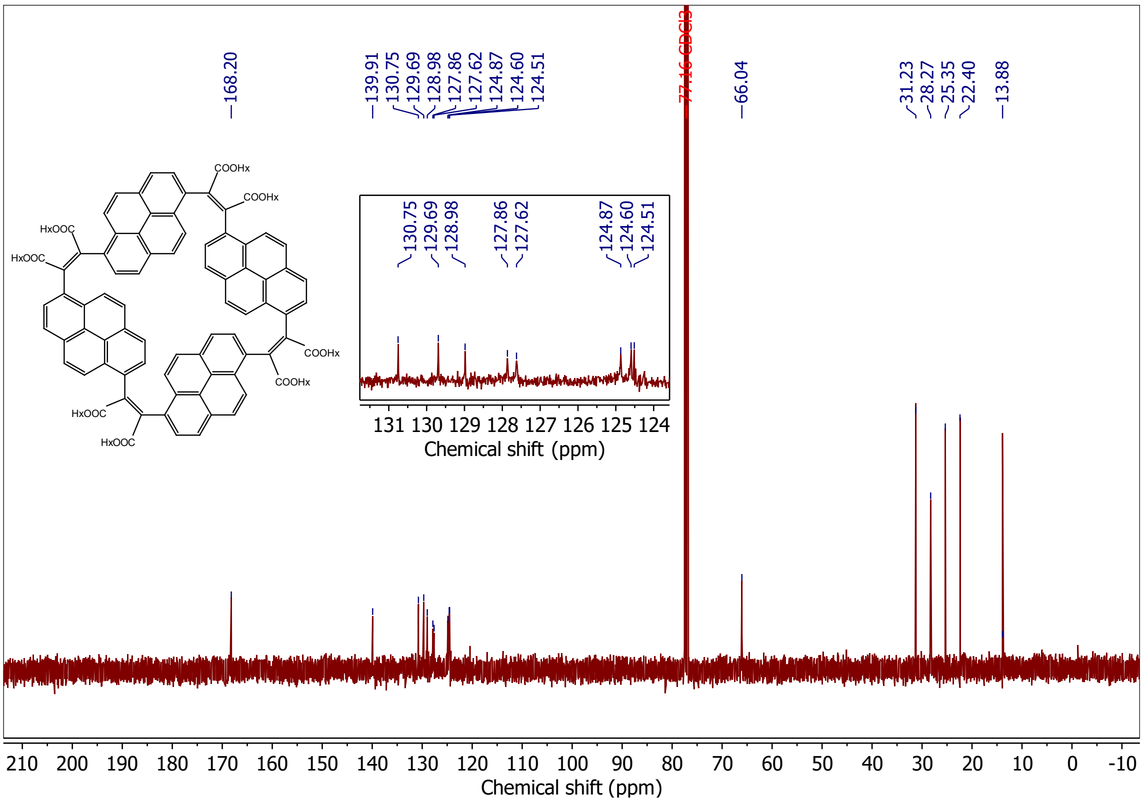

**Figure S42.** $^{13}$C NMR (101 MHz, $CDCl_3$) of macrocycle **PCyc-Hx**.

## 3 Photoluminescence spectra

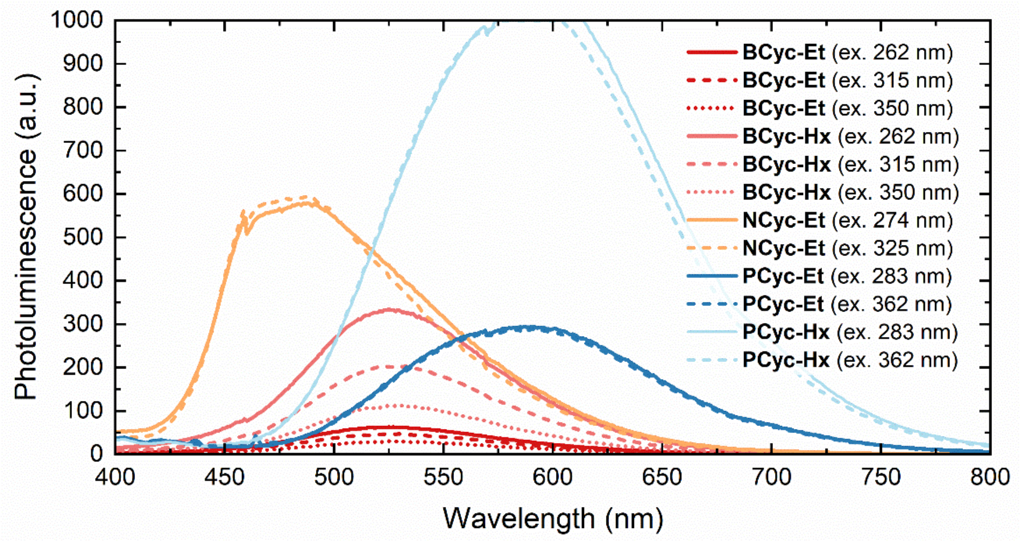


**Figure S43.** Photoluminescence spectra of the macrocycles recorded at different excitation wavelengths (shown in brackets) in $CHCl_3$ solution (5 µM) with the detector voltage set to 'high' (800 V). The dashed lines show the spectra provided in Figure 1.

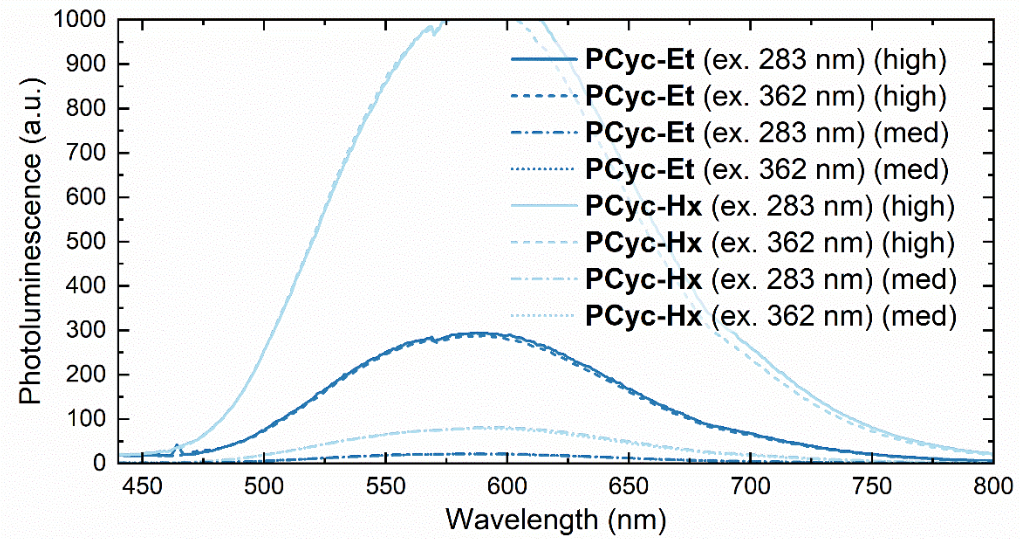


**Figure S44.** Photoluminescence spectra of **PCyc-Et** and **PCyc-Hx** recorded at two different excitation wavelengths in $CHCl_3$ solution (5 µM) with the detector voltage set to 'high' (800 V) or 'medium' (600 V).

## 4 Cyclic voltammetry (CV) in solution

### Chemicals and materials

Ferrocene (Fc, 98%) was obtained from Aldrich. 1,2-Dichloroethane (DCE, anhydrous, 99.8%), N,N-dimethylformamide (DMF, anhydrous, 99.8%), propylene carbonate (PC, anhydrous, 99.7%), and

tetrabutylammonium hexafluorophosphate ($NBu_4PF_6$, >98%) were from Sigma-Aldrich. Silver wire (0.5 mm dia.), platinum wires (0.5 mm, 0.1 mm and 0.025 mm diameter) were from Mint of Poland. 2 mm diameter disk platinum electrode (CHI303) was from CHi Instruments.

**Voltammetric measurements**

Details provided in the methods section of the paper.

## 4.1 Electroreduction and -oxidation of the macrocycles at arbitrary concentration in 1,2-dichloroethane

**Electroreduction:** For **BCyc-Et**, **PCyc-Et**, and **PCyc-Hx**, the mid-peak potential vs. $Fc/Fc^+$ (the midpoints between the anodic and cathodic peak potentials) were determined and are provided in the main part of the paper. For **NCyc-Et** and **ACyc-Et**, where the peak potentials could not be determined, the inflection-point potentials were determined by differentiating the cyclic voltammograms (Figure S45).

**Electrooxidation:** As the electrooxidation was chemically irreversible for all macrocycles, the inflection-point potentials were determined by differentiating the cyclic voltammograms (Figure S45).

The measurements were carried out at arbitrary concentration as the small amounts available for some of the macrocycles prevented the preparation of solutions with defined concentration. While the concentration has an impact on the current, the redox potential does not depend on the concentration.

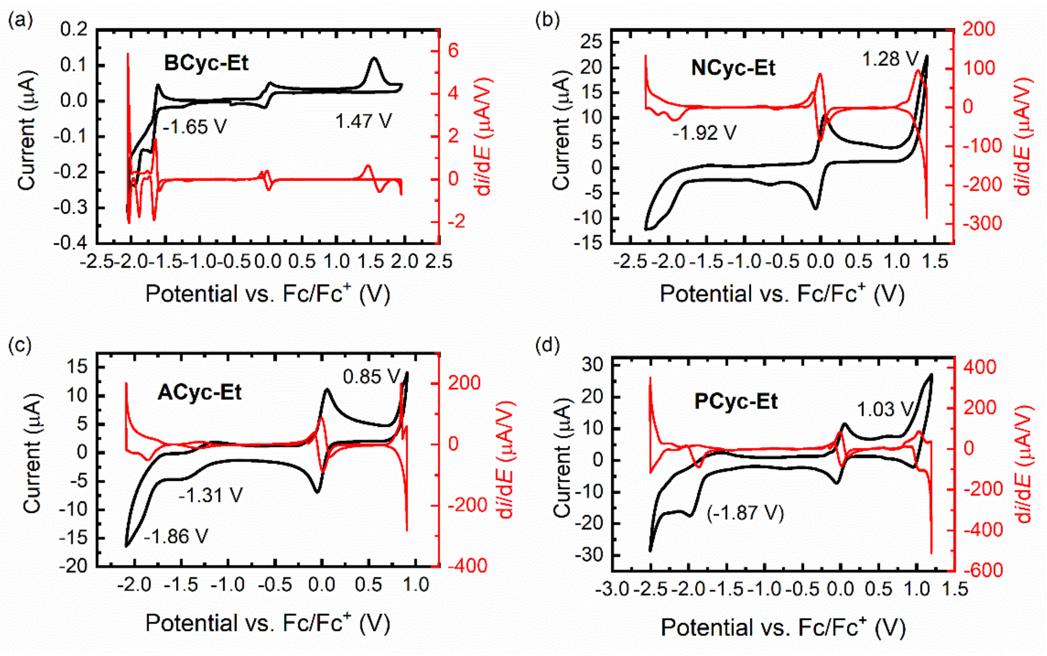


**Figure S45.** Cyclic voltammograms of (a) **BCyc-Et**, (b) **NCyc-Et**, (c) **ACyc-Et**, and (d) **PCyc-Et** and their first derivatives (with ferrocene as the internal reference), recorded in 1,2-dichloroethane (DCE) on platinum disk electrodes of 2 mm diameter. Scan rate v = 0.1 V $s^{-1}$, supporting electrolyte: 0.1 M $NBu_4PF_6$.

## 4.2 Electroreduction of BCyc-Et at defined concentrations

Cyclic voltammetry of **BCyc-Et** solutions in 1,2-dichloroethane (DCE), N,N-dimethylformamide (DMF), and propylene carbonate (PC), all containing 0.1 M $NBu_4PF_6$ as supporting electrolyte, were performed with 2 mm diameter platinum disk electrode. The obtained voltammograms (Figure S46) suggest two quasi-reversible electroreduction steps. With the intention of getting an indication of the stoichiometry and kinetic parameters of these steps, simulated voltammograms were fitted to the obtained voltammograms using DigiSim 3.03b software, assuming two consecutive one-electron reductions steps:

**BCyc-Et** + $e^-$ ⇌ **BCyc-Et**$^-$ $\quad E^{0}$'(1), $k_0$(1), α(1) $\quad$ (1)

**BCyc-Et**$^-$ + $e^-$ ⇌ **BCyc-Et**$^{2-}$ $\quad E^{0}$'(2), $k_0$(2), α(2) $\quad$ (2)

Similarly to non-substituted **BCyc** (also known as PCT),[9] the most reduced form in the assumed process (**BCyc-Et**$^{2-}$) is considered to be a reductant strong enough to be oxidized homogeneously, most probably by $H_2O$ and $O_2$ traces in solvents:

**BCyc-Et**$^{2-}$ (+ oxidant) $\xrightarrow{k}$ **BCyc-Et**$^-$ (+ reduced oxidant) $\quad$ (3)

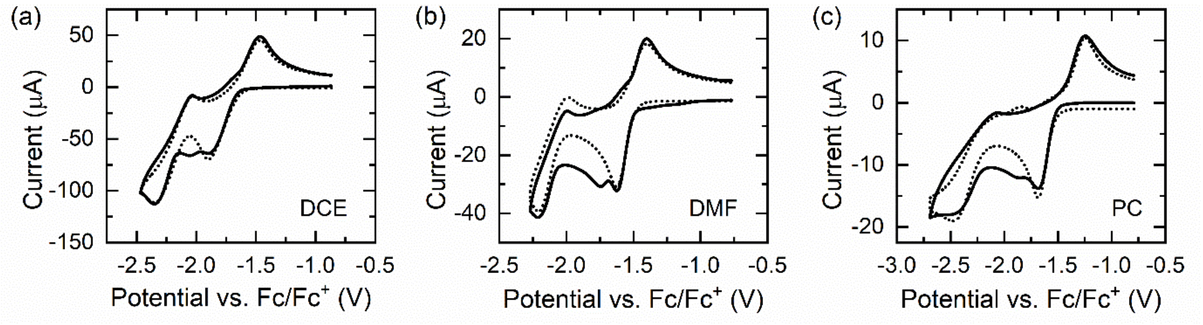


**Figure S46.** Cyclic voltammograms of **BCyc-Et** in (a) DCE, (b) DMF, and (c) PC recorded on platinum disk electrodes of 2 mm diameter (solid lines). Scan rate $v$ = 0.1 V $s^{-1}$, supporting electrolyte: 0.1 M $NBu_4PF_6$. Simulated cyclic voltammograms assuming two one-electron reduction steps (dotted lines) with fitted kinetic parameters, formal potentials, and diffusion coefficients. All parameters for the simulations are listed in Table S1.. Other parameters for the simulations: (a) uncompensated resistance ($R_u$): 1 kΩ, double layer capacitance ($C_{dl}$): 10 μF; (b) $R_u$ = 0 Ω, $C_{dl}$ = 15 μF; (c) $R_u$ = 0 Ω, $C_{dl}$ = 10 μF. Temperature: 298.2 K.

The recorded cyclic voltammograms of **BCyc-Et** in the different solvents (DCE, DMF, PC) are shown in Figure S46 (solid lines), together with the simulated voltammograms (dotted lines) assuming two consecutive one-electron reduction steps with fitted thermodynamic ($E^{0}$'(1,2) – formal potentials of reactions (1 and 2)), kinetic ($k_0$(1,2) – standard electron transfer (ET) rate constants, $\alpha$(1,2) – ET coefficients, $k$ – rate constant of pseudo-1st order reaction of homogeneous regeneration of **B-Et-Cycle**$^-$

(eq. 3)) and hydrodynamic ($D$ – diffusion coefficient, $r_H$ – effective hydrodynamic radius of **BCyc-Et**) parameters according to the abovementioned mechanism. The parameters found by fitting are listed in gray cells in Table S1. Hydrodynamic radii ($r_H$) were calculated from fitted diffusion coefficients and solvent viscosities using the Stokes-Einstein equation. Additional peaks of low current magnitude that are visible in the recorded voltammograms can be caused by adsorption of **BCyc-Et** on the platinum electrode.

Contrary to the previous approach used for the analysis of the voltammograms of **BCyc**, where diffusion coefficients were obtained from steady-state voltammograms recorded with an ultramicroelectrode (UME), here the diffusion limiting current could not be unambiguously read from voltammograms recorded with a 25 μm diameter UME (Figure S47). This is due to the slower kinetics of the electron transfer for **BCyc-Et** than for **BCyc**. Therefore, diffusion coefficients ($D$) of **BCyc-Et** were obtained by fitting to voltammograms recorded with a bigger (2 mm diameter) electrode. To verify the procedure of finding $D$ by fitting cyclic voltammograms recorded at a larger disk electrode, we applied the procedure to the voltammograms of **BCyc**. Diffusion coefficients of **BCyc** obtained this way differ less than 10% from the values obtained from the UME measurements.

**Table S1.** Physicochemical parameters of the electroreduction of **BCyc-Et** assuming two one-electron reduction steps (diffusion coefficient, $D$; concentration, $c$; hydrodynamic radius, $r_H$; formal potentials, $E^{0}$'; standard electron transfer rate constants, $k_0$; electron transfer coefficients, α) in three different solvents (dynamic viscosity, $\eta$; dipole moment, $\mu$) at 298.15 K. Parameters from fitting simulated voltammograms to voltammograms obtained using platinum disk electrodes of 2 mm diameter highlighted in gray. Hydrodynamic radii were calculated using the Stokes-Einstein equation and viscosities from literature.

| Solvent | $\eta$ (mPa·s) | $\mu$ (D) | $D$ (cm$^2$ s$^{-1}$) | $c$ (mmol dm$^{-3}$) | $r_H$ (nm) | $E^{0}$'(1) (V) | $k_0$(1) (cm s$^{-1}$) | α(1) | $E^{0}$'(2) (V) | $k_0$(2) (cm s$^{-1}$) | α(2) | $k$ (s$^{-1}$) |
|---|---|---|---|---|---|---|---|---|---|---|---|---|
| 1,2-dichloro-ethane (DCE) | 0.7644 (Ref. [10]) | 2.94 (Ref. [11]) | $5.0 \times 10^{-6}$ | 14.5 | 0.56 | -1.66 | $3.1 \times 10^{-4}$ | 0.49 | -2.12 | $1.6 \times 10^{-3}$ | 0.4 | 0.67 |
| N,N-Dimethyl-formamide (DMF) | 0.864 (Ref. [12]) | 3.82 (Ref. [11]) | $4.2 \times 10^{-6}$ | 6.2 | 0.59 | -1.52 | $6.5 \times 10^{-4}$ | 0.58 | -2.11 | $1.1 \times 10^{-3}$ | 0.48 | 0.4 |
| Propylene carbonate (PC) | 2.4711 (Ref. [13]) | 5.36 (Ref. [14]) | $1.4 \times 10^{-6}$ | 5.2 | 0.62 | -1.48 | $4.8 \times 10^{-5}$ | 0.55 | -2.03 | $10^{-5}$ | 0.32 | 0.4 |

**Discussion of the simulation results**

The hydrodynamic radii obtained from fitting voltammograms simulated under the assumption of two one-electron reduction steps ($r_H$ in Table S1) roughly correspond to the size of the macrocycle. However, estimating the hydrodynamic radius from the molecular geometry is difficult and neglects that interactions with solvent molecules can increase the actual hydrodynamic radius. Such interactions can be expected particularly for molecules with polar functional groups, such as ester groups.

Under the assumption of two two-electron reduction steps, larger hydrodynamic radii would be obtained from the simulations, which do not correspond well with the bare macrocycle but may in line with an increased hydrodynamic radius because of interactions with solvent molecules.

### Further measurements

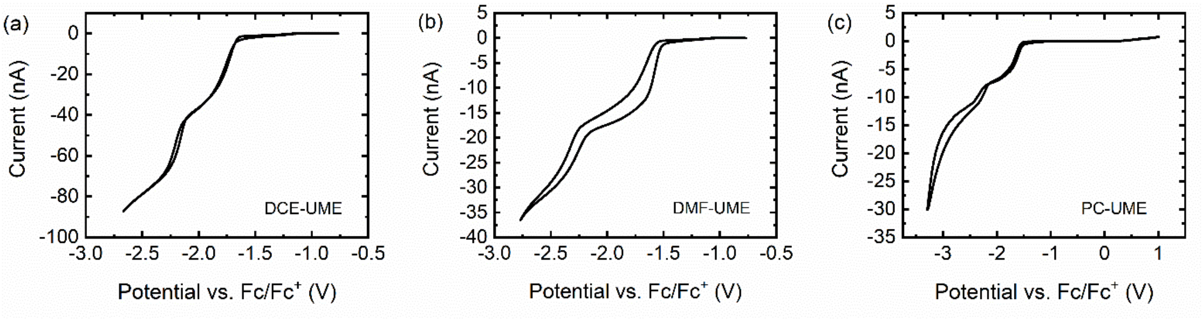


**Figure S47.** Steady-state cyclic voltammograms of **BCyc-Et** in (a) DCE, (b) DMF, and (c) PC recorded on platinum disk ultramicroelectrodes of 25 µm diameter. Scan rate $v$ = 10 mV s$^{-1}$, supporting electrolyte: 0.1 M $NBu_4PF_6$.

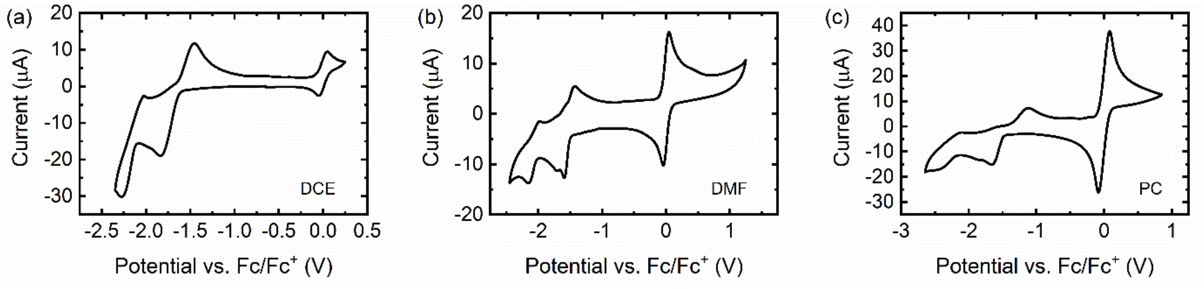


**Figure S48.** Cyclic voltammograms of **BCyc-Et** with ferrocene in (a) DCE, (b) DMF, and (c) PC recorded on platinum disk electrodes of 2 mm diameter. Scan rate $v$ = 0.1 V s$^{-1}$, supporting electrolyte: 0.1 M $NBu_4PF_6$.

# 5 Electron paramagnetic resonance (EPR) spectroelectrochemistry

### Chemicals

All chemicals were purchased from Sigma-Aldrich: 1,2-dichloroethane (DCE, 99.8%), tetrabutylammonium hexafluorophosphate ($NBu_4PF_6$, ≥99.0%), ferrocene (Fc, 98%), 4-amino-2,2,6,6-tetramethylpiperidinyl-1-oxyl (4-amino-TEMPO, 97%).

### Electrochemical measurement set-up

A three-electrode set-up was used for all electrochemical measurements. All electrochemical measurements were carried out in a quartz EPR tube (Wilmad, outer diameter 4 mm), sealed with a suba-seal rubber septum (**Figure S49**). The working (WE) and counter electrodes (CE) were two Teflon-coated Pt wires (ADVENT, purity 99.99%, bare wire diameter = 0.125 mm), coiled to enhance their surface area. The working electrode was encased in a glass capillary to avoid short-circuiting and

maintain the wire at the bottom of the EPR tube that is inserted into the EPR cavity. An Ag/AgCl reference electrode (RE, calibrated using a master saturated calomel electrode SCE) was used for aqueous electrochemical measurements. Teflon-coated silver wire (ADVENT, purity 99.99%, bare wire diameter = 0.125 mm) was used as a pseudo-reference electrode (pseudo-RE) for the measurements in DCE, with 5 mM ferrocene (Fc) added to calibrate the potential. The EPR tube was purged with nitrogen gas prior to electrochemical measurements performed with a µAutolabIII potentiostat in combination with NOVA software.

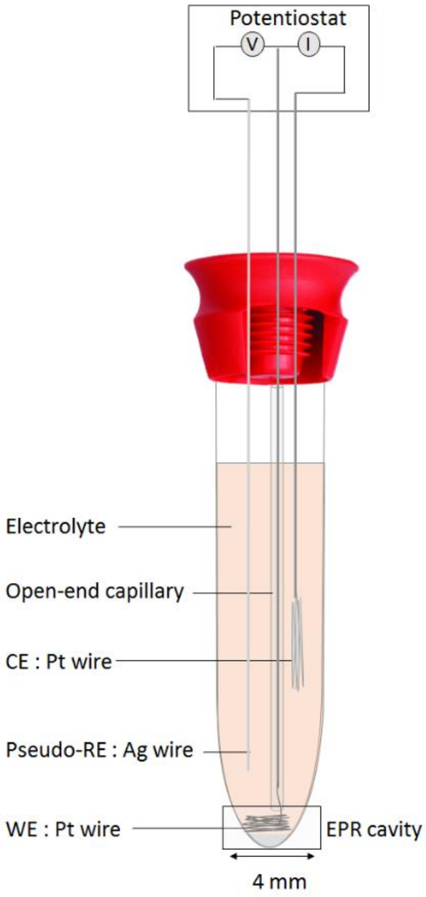


**Figure S49.** Spectroelectrochemical cell assembled in an EPR tube. The tube is inserted into the EPR setup, but only the bottom 2 mm of the tube are placed in the actual EPR cavity.

**EPR measurement set-up**

Continuous wave X-band EPR measurements were performed at room temperature in an X-band Super High Sensitivity Probehead ER 4122SHQE and an EMX-T-DU/L Bruker spectrometer.

The bottom 2 mm of the EPR tube containing the electrochemical cell, i.e. only the working electrode (see Figure S49), was placed inside the EPR cavity (resulting in Q values of approx. 1300-4500 due to the conductivity of the sample). After insertion of the EPR tube into the cavity and optimisation of its position, the electrodes were connected to the potentiostat and the electrochemical and EPR measurements were carried out simultaneously.

**Sample preparation**

**BCyc-Et** or 4-amino-TEMPO was dissolved in 0.4 ml DCE to a final concentration of 5 mM and supplemented with 0.1 M $NBu_4PF_6$. Aqueous electrochemical measurements with 4-amino-TEMPO (5 mM) were carried out in 0.5 M $Na_2CO_3$ in milliQ water, adjusted to pH 8.0.

## 5.1 EPR spectroelectrochemical measurements of 4-amino-TEMPO

To test the measurement set-up, electrochemical EPR measurements were first carried out on the EPR active compound 4-amino-TEMPO under aqueous conditions. As can be seen in **Figure S50**A, the cyclic voltammogram showed a redox potential $E_{m,\,pH\,8.0}$ = 0.631 V vs. Ag/AgCl (0.836 V vs. SHE) that is consistent with previously reported values ($E_{m,\,pH\,7.0}$ = 0.874 V vs. SHE).[15] The in operando EPR measurements show that the nitroxide EPR signal changes upon the redox reaction, but it does not disappear completely (Figure S50B), which is expected given that the redox reaction occurs just at the electrode.

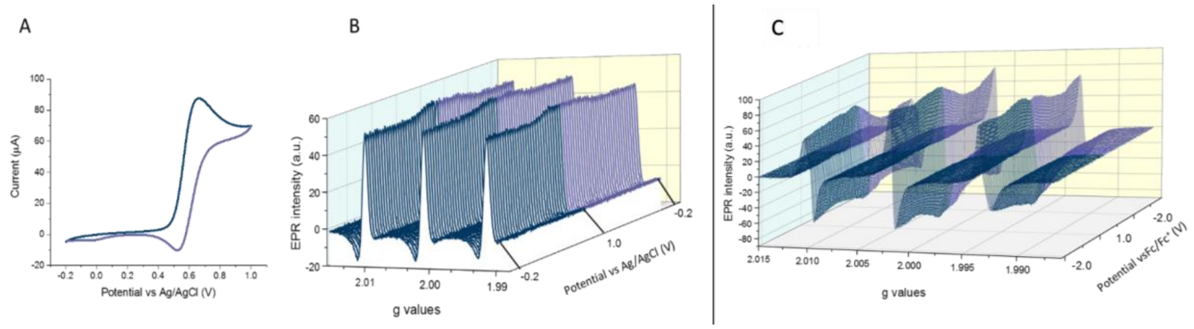


**Figure S50.** Spectroelectrochemical measurements of 5 mM 4-amino-TEMPO in aq. 0.5 M $Na_2CO_3$ at pH 8.0: **(A)** Cyclic voltammogram recorded at a scan rate of 5 mV $s^{-1}$ and **(B)** set of incremented EPR spectra registered while cycling the applied potential at 5 mV $s^{-1}$. **(C)** Spectroelectrochemical measurements of 5 mM 4-amino-TEMPO in 0.4 ml DCE with 0.1 M $nBu_4PF_6$: set of incremented EPR spectra registered while cycling the applied potential at a scan rate of 5 mV $s^{-1}$. Experimental conditions (EPR): microwave power = 20 mW, microwave frequency = 9.863 GHz **(B)**, 9.844 GHz **(C)**, modulation amplitude = 0.1 mT, sweep time = 5 s per scan, quality factor of the resonator as reported by the built-in Q indicator in the Xepr program = 1300 **(B)**, 4800 **(C)**.

The corresponding experiment under non-aqueous conditions (in DCE) showed more sluggish electron transfer, but redox cycling is nonetheless evident from the in operando electrochemical EPR measurements (**Figure S50**C).

## 5.2 EPR spectroelectrochemical measurements of BCyc-Et

In contrast to the measurements of 4-amino-TEMPO, in operando electrochemical EPR measurements with the **BCyc-Et** only gave EPR spectra with flat lines (**Figure S51**). Although the CV (Figure S51A) and the control measurements with 4-amino-TEMPO (Figure S50) suggest that the set-up needs to be further improved for optimal in-operando measurements, they enable us to conclude that it is highly unlikely that a paramagnetic intermediate is formed during redox cycling of **BCyc-Et**, corroborating that it does not proceed in single electron transfer steps.

Measurements at various scan rates including 5 mV $s^{-1}$ (the scan rate used for the measurements shown in Figure S50) were carried out, with no EPR signal appearing in any of these measurements. In situ chronoamperometry coupled to EPR measurements was also carried out. A potential range of -2.5 V to -1.0 V (vs. $Fc/Fc^+$) was investigated by holding the potential for more than 300 seconds at different values while recording EPR spectra. In agreement with the result obtained from in situ CV-EPR measurements, no EPR signal was observed over the scanned potential range.

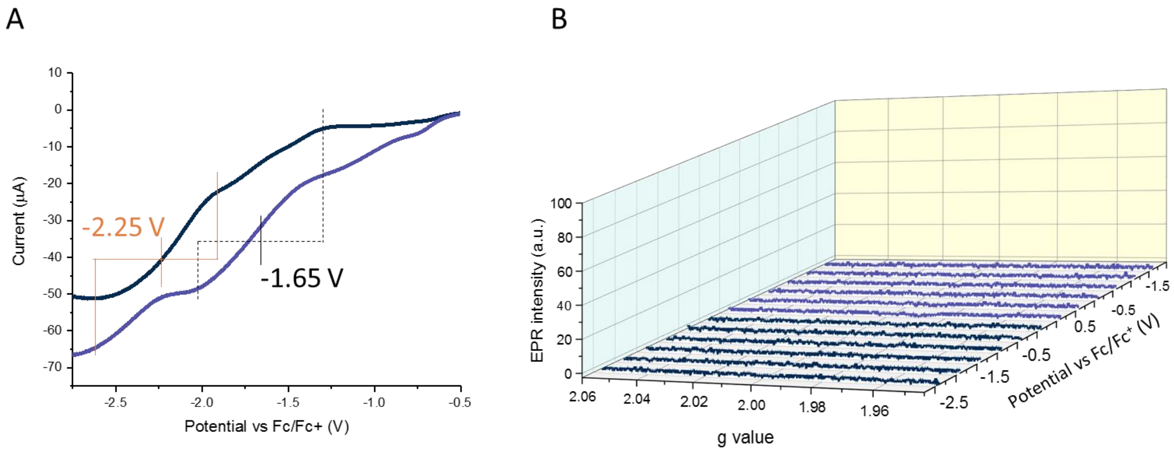


**Figure S51.** Spectroelectrochemical measurements of 5 mM **BCyc-Et** in 0.4 ml DCE with 0.1 M $NBu_4PF_6$. **(A)** Cyclic voltammogram recorded at a scan rate of 0.1 V $s^{-1}$ after subtraction of the blank measurement. **(B)** Set of incremented EPR spectra registered while cycling the applied potential at a scan rate of 0.1 V $s^{-1}$. Experimental conditions: microwave power = 100 mW, microwave frequency = 9.854 GHz, modulation amplitude = 0.1 mT, sweep time = 5 s, quality factor of the resonator as reported by the built-in Q indicator in the Xepr program = 3900.

# 6 Computational analysis

## 6.1 Redox potentials

**Table S2.** Calculated redox potentials vs. $Fc/Fc^{+}$ for the different redox couples of the macrocycles.

| | -6/-4 | -4/-2 | -2/-1 | -1/0 | 0/+1 | +1/+2 | +2/+4 | +4/+6 | -2/-4 (trip.) | +2/+4 (trip.) |
|---|---|---|---|---|---|---|---|---|---|---|
| **BCyc** | -5.43 | -4.01 | -2.47 | -2.08 | 0.64 | 1.14 | 2.65 | 4.50 | -4.10 | 2.80 |
| **BCyc-Et** | -3.74 | -2.48 | -1.78 | -1.66 | 1.52 | 1.91 | 3.00 | 4.83 | -2.62 | 3.15 |
| **NCyc-Et** | -3.15 | -2.16 | -1.96 | -1.82 | 1.41 | 1.72 | 2.37 | 3.66 | -2.33 | 2.43 |
| **ACyc-Et** | -3.27 | -2.22 | -1.90 | -1.62 | 0.72 | 1.19 | 1.68 | 3.28 | -2.40 | 1.87 |
| **PCyc-Et** | -2.97 | -2.17 | -1.84 | -1.68 | 1.01 | 1.45 | 1.83 | 3.10 | -2.20 | 2.02 |

## 6.2 Nucleus-independent chemical shifts (NICS)

**Table S3.** $NICS(0)_{zz}$ values of the macrocycles in the different charge and spin states.

| | BCyc | | BCyc-Et | | NCyc-Et | | ACyc-Et | | PCyc-Et | |
|---|---|---|---|---|---|---|---|---|---|---|
| **Charge state** | **$NICS(0)_{zz}$ (ppm)** singlet | triplet | **$NICS(0)_{zz}$ (ppm)** singlet | triplet | **$NICS(0)_{zz}$ (ppm)** singlet | triplet | **$NICS(0)_{zz}$ (ppm)** singlet | triplet | **$NICS(0)_{zz}$ (ppm)** singlet | triplet |
| **-6** | -45.95 | | -2.46 | | -8.07 | | -3.55 | | -13.68 | |
| **-4** | 96.70 | -43.24 | 9.98 | -14.38 | 7.93 | -16.75 | 10.07 | -18.43 | 11.10 | -14.94 |
| **-2** | -38.65 | | -23.68 | | -16.75 | | -6.09 | | -10.98 | |
| **-1** | -8.32 | | -3.87 | | -3.07 | | -2.12 | | -1.67 | |
| **0** | 10.98 | -35.79 | 7.51 | -23.57 | 5.49 | -15.56 | 0.52 | -4.41 | 4.17 | -8.20 |
| **+1** | -6.90 | | -3.57 | | -2.06 | | -0.89 | | 1.28 | |
| **+2** | -33.48 | | -20.87 | | -13.31 | | -3.02 | | -2.80 | |
| **+4** | 34.63 | -32.34 | 5.99 | -17.67 | 3.93 | -20.78 | 6.00 | -3.29 | 3.43 | -0.93 |
| **+6** | -27.72 | | 4.69 | | -17.96 | | -15.18 | | -2.62 | |

**Table S4.** Isotropic NICS(0) values of the macrocycles in the different charge and spin states.

| | BCyc | | BCyc-Et | | NCyc-Et | | ACyc-Et | | PCyc-Et | |
|---|---|---|---|---|---|---|---|---|---|---|
| **Charge state** | **NICS(0) (ppm)** singlet | triplet | **NICS(0) (ppm)** singlet | triplet | **NICS(0) (ppm)** singlet | triplet | **NICS(0) (ppm)** singlet | triplet | **NICS(0) (ppm)** singlet | triplet |
| **-6** | -16.75 | | -5.26 | | -5.89 | | -2.97 | | -9.13 | |
| **-4** | 30.42 | -16.23 | -0.53 | -9.09 | -0.18 | -8.78 | 1.09 | -8.53 | -1.04 | -9.79 |
| **-2** | -15.11 | | -11.97 | | -8.36 | | -2.61 | | -8.74 | |
| **-1** | -5.34 | | -5.79 | | -4.23 | | -1.32 | | -5.91 | |
| **0** | 0.44 | -14.54 | -2.75 | -12.22 | -2.18 | -8.28 | -0.90 | -1.71 | -4.57 | -7.77 |
| **+1** | -5.33 | | -6.16 | | -4.40 | | -1.37 | | -4.98 | |
| **+2** | -14.11 | | -11.69 | | -7.87 | | -2.31 | | -5.83 | |
| **+4** | 8.35 | -13.77 | -2.16 | -10.32 | -1.86 | -9.89 | 0.36 | -2.53 | -3.57 | -4.38 |
| **+6** | -12.08 | | -2.49 | | -8.86 | | -7.02 | | -3.84 | |

## 6.3 Current densities

For **BCyc** and **BCyc-Et**, we also computed current densities using the gauge-including magnetically induced current (GIMIC) method[16] based on PBE0/def2-SVP computations carried out in Turbomole 7.4.[17] Currents were integrated along a plane bisecting the C=C double bond in a vinylene unit. These results are presented in Table S5. Table S5 also presents NICS(0)$_{zz}$ values computed according to a simple conductor loop model. This model is given according to $\mathrm{NICS}(0)_{zz} = \frac{\mu_0 I}{2R}$ where $\mu_0$ is the vacuum permeability, $I$ is the induced current in nA/T obtained from GIMIC, and $2R$ is the diameter of the molecule (9.63 Å for **BCyc**; 9.23 Å for **BCyc-Et**). Comparison to the NICS(0)$_{zz}$ values computed directly from DFT shows excellent agreement for the strongly globally aromatic cases (-6/-2/+2/+6 of **BCyc** and -2/+2 of **BCyc-Et**). The good agreement between the two approaches suggests that the magnetic properties in these states are indeed dominated by just one global ring current around the perimeter. The most notable difference is found for the neutral states where the GIMIC analysis indicates only minuscule global antiaromaticity suggesting that the deshielding found at the center of the macrocycles derives mostly from diatropic currents in the benzene units rather than global paratropic currents.

**Table S5.** Integrated currents and NICS(0)$_{zz}$ values of **BCyc** and **BCyc-Et** in the different charge states (always considering singlet multiplicity). NICS(0)$_{zz}$ values are given according to the model outlined in the text above and compared with the values obtained directly from DFT.

| | **BCyc** | | | **BCyc-Et** | | |
|---|---|---|---|---|---|---|
| **Charge state** | **Current (nA/T)** | **NICS(0)$_{zz}$ (ppm) model** | **DFT** | **Current (nA/T)** | **NICS(0)$_{zz}$ (ppm) model** | **DFT** |
| **-6** | -36.193 | -47.20 | -45.95 | -5.860 | -7.98 | -2.46 |
| **-4** | 61.557 | 80.27 | 96.70 | 2.701 | 3.68 | 9.98 |
| **-2** | -31.566 | -41.16 | -38.65 | -20.331 | -27.69 | -23.68 |
| **0** | 0.772 | 1.01 | 10.98 | 1.118 | 1.52 | 7.51 |
| **+2** | -27.767 | -36.21 | -33.48 | -17.164 | -23.38 | -20.87 |
| **+4** | 18.466 | 24.08 | 34.63 | 3.704 | 5.04 | 5.99 |
| **+6** | -24.148 | -31.49 | -27.72 | -1.554 | -2.12 | 4.69 |

## 6.4 Visualization of chemical shielding tensors (VIST)

### 6.4.1 VIST plots of BCyc in different charge and spin states

**BCyc**

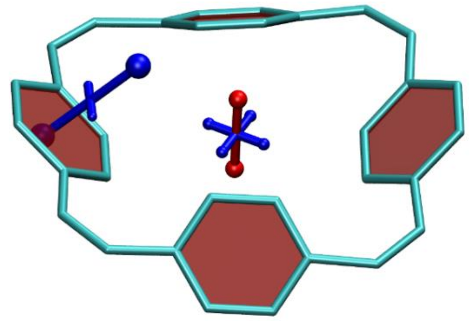

**BCyc (triplet)**

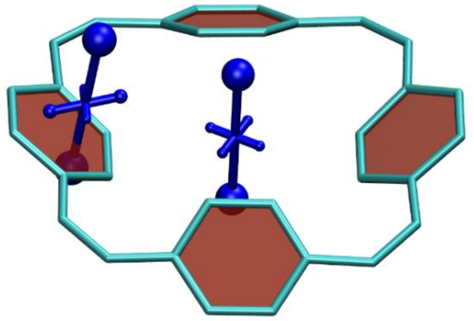

**BCyc$^{2-}$**

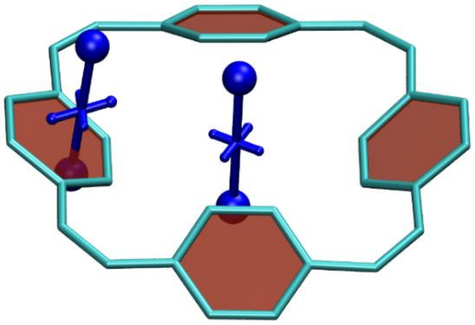

**BCyc$^{2+}$**

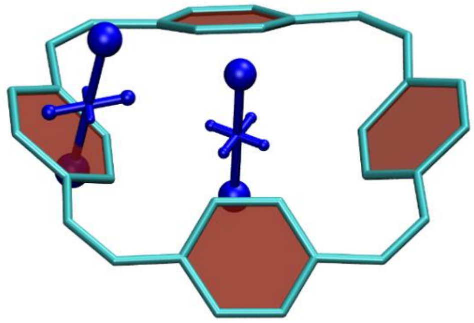

**BCyc$^{4-}$**

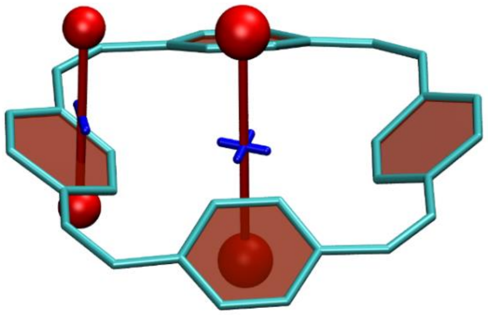

**BCyc$^{4+}$**

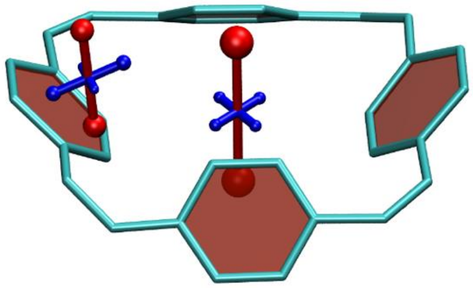

**BCyc$^{4-}$ (triplet)**

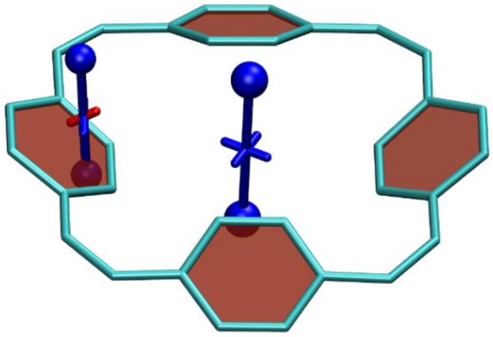

**BCyc$^{4+}$ (triplet)**

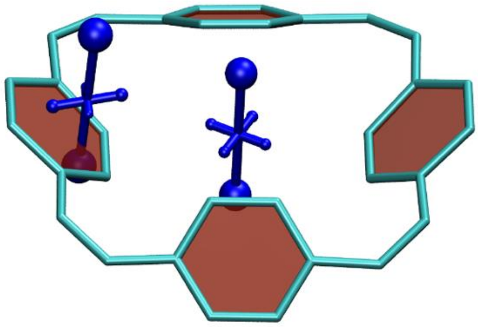

**BCyc$^{6-}$**

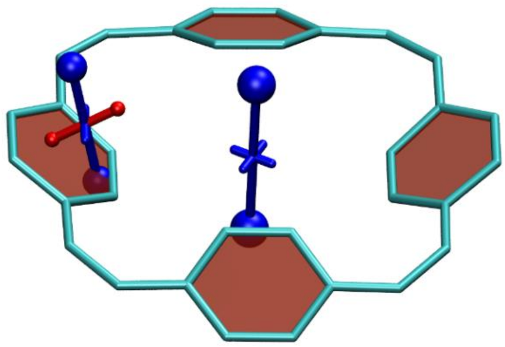

**BCyc$^{6+}$**

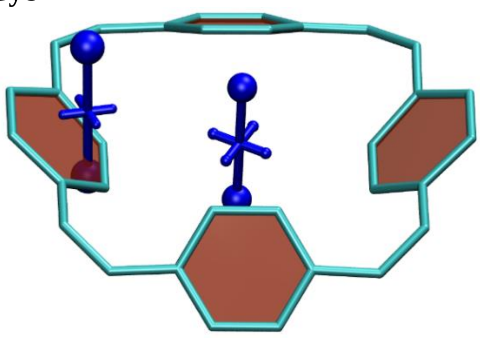

### 6.4.2 VIST plots of BCyc-Et in different charge and spin states

**BCyc-Et**

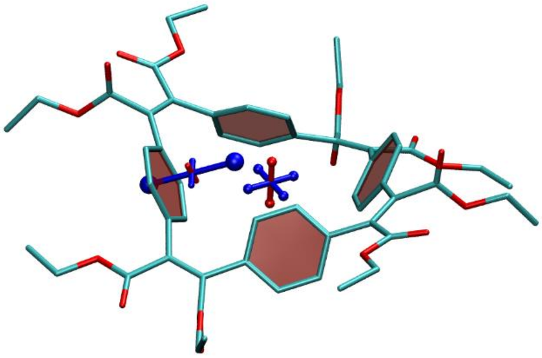

**BCyc-Et (triplet)**

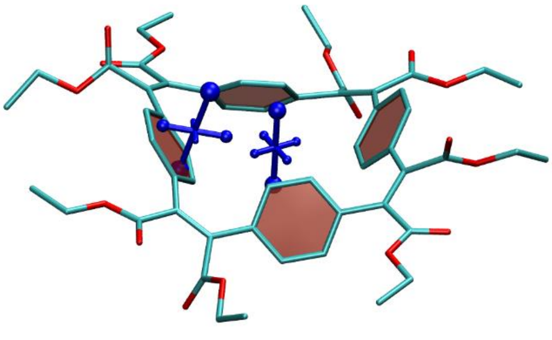

**BCyc-Et$^{2-}$**

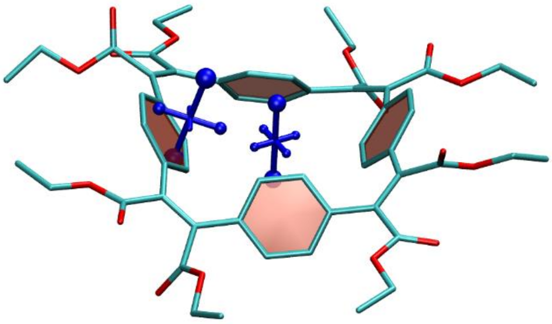

**BCyc-Et$^{2+}$**

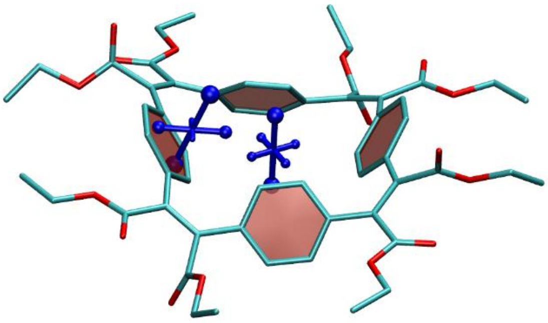

**BCyc-Et$^{4-}$**

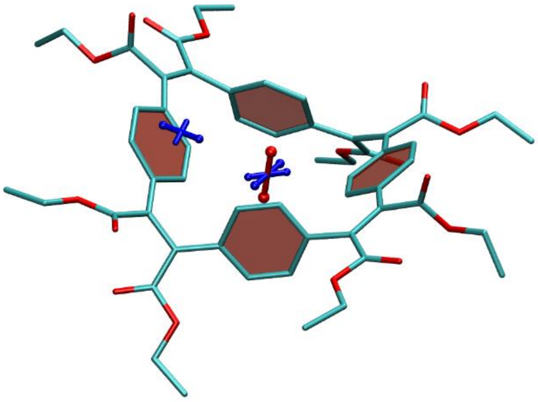

**BCyc-Et$^{4+}$**

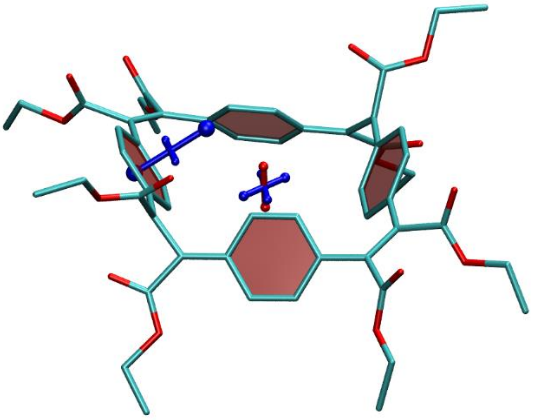

**BCyc-Et$^{4-}$ (triplet)**

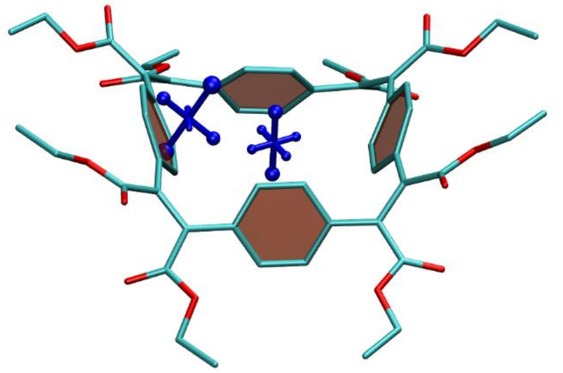

**BCyc-Et$^{4+}$ (triplet)**

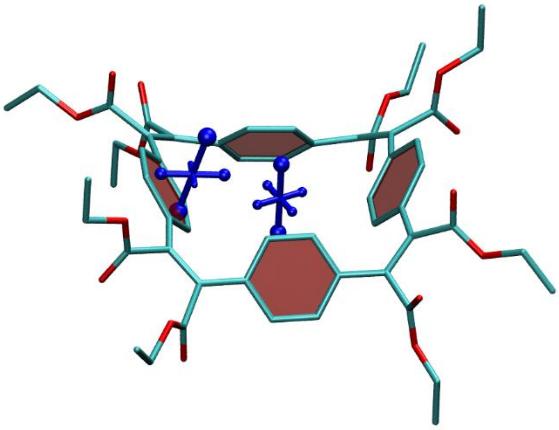

**BCyc-Et$^{6-}$**

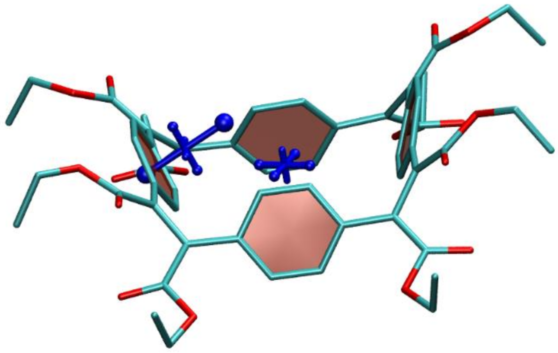

**BCyc-Et$^{6+}$**

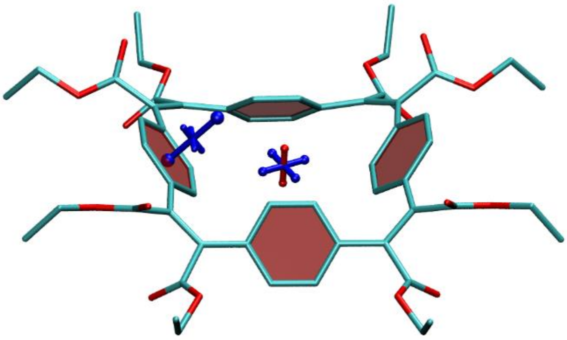

### 6.4.3 VIST plots of NCyc-Et in different charge and spin states

**NCyc-Et**

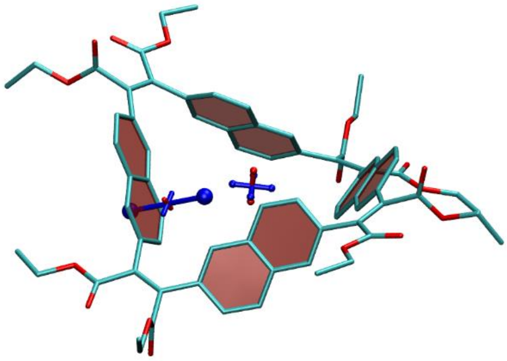

**NCyc-Et$^{2-}$**

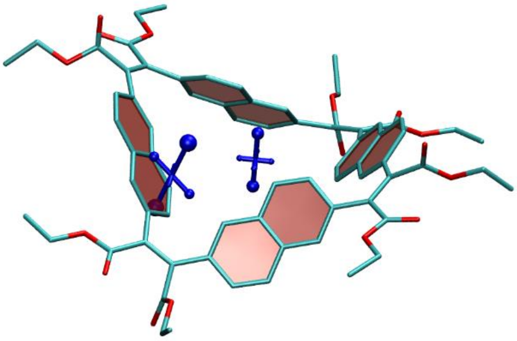

**NCyc-Et$^{2+}$**

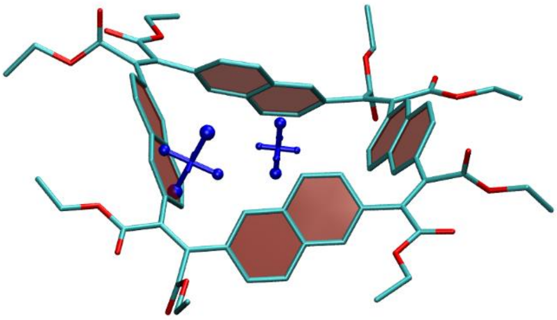

### 6.4.4 VIST plots of ACyc-Et in different charge states

**ACyc-Et**

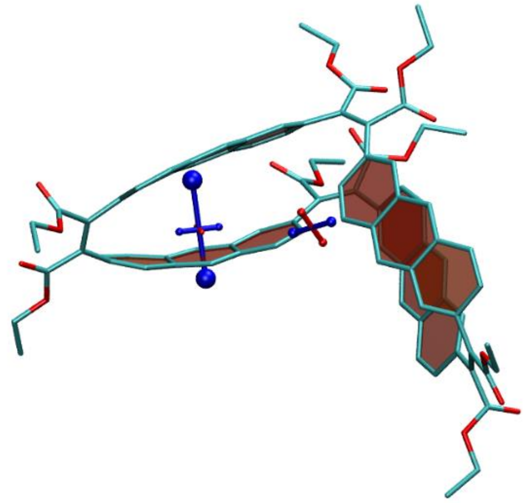

**ACyc-Et$^{2-}$**

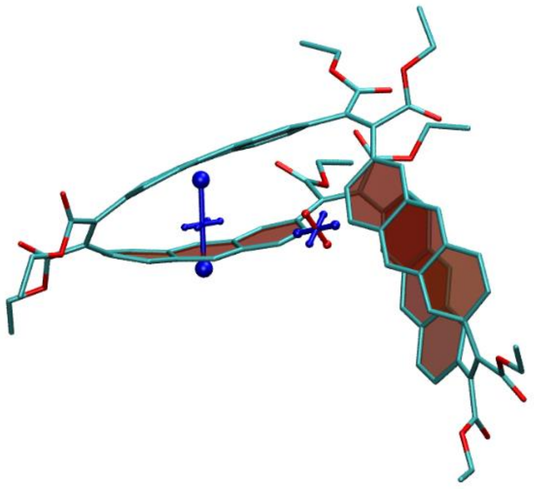

**ACyc-Et$^{2+}$**

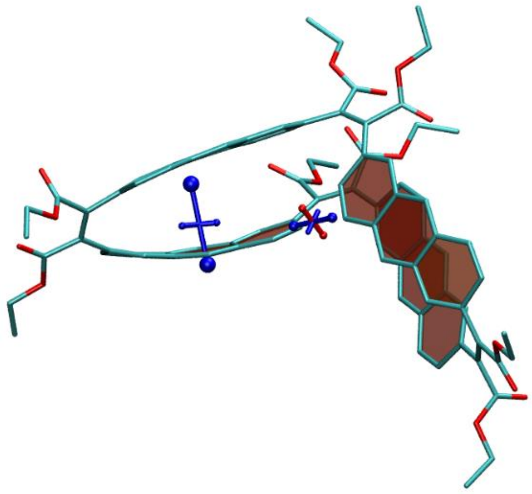

### 6.4.5 VIST plots of PCyc-Et in different charge states

**PCyc-Et**

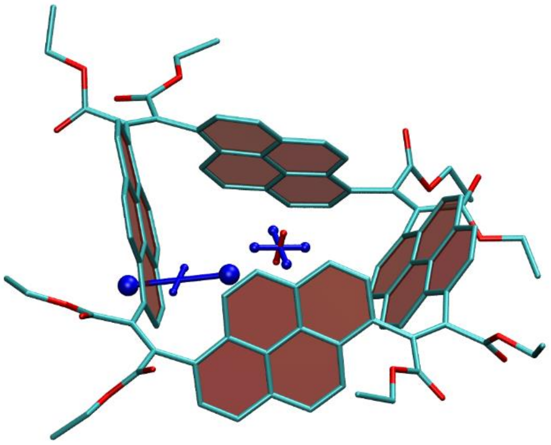

| PCyc-Et$^{2-}$ | PCyc-Et$^{2+}$ |
|---|---|
| 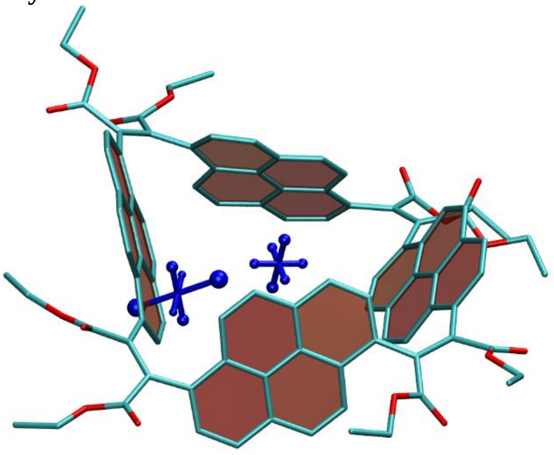 | 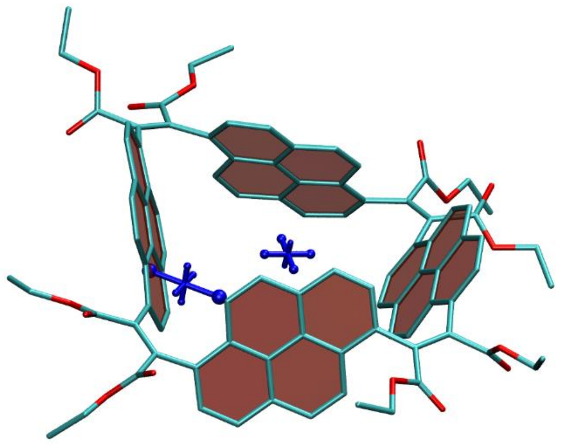 |

## 6.5 Electron density difference

### 6.5.1 Electron density difference plots of BCyc

Isovalues: -0.003 *e* for anions, +0.003 *e* for cations.

| BCyc$^{2-}$ | BCyc$^{2+}$ |
|---|---|
| 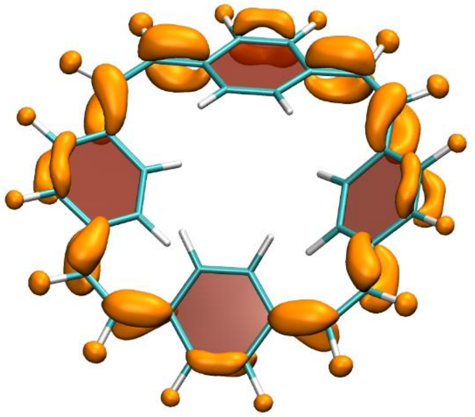 | 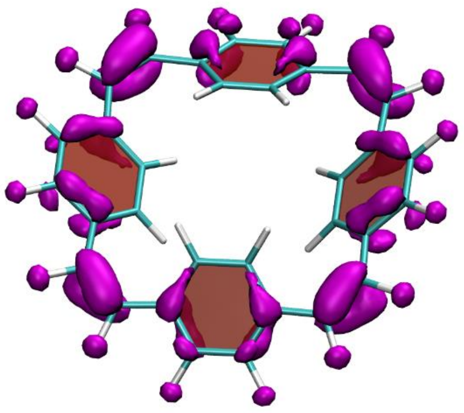 |
| **BCyc$^{2-}$ (top)** | **BCyc$^{2+}$ (top)** |
| 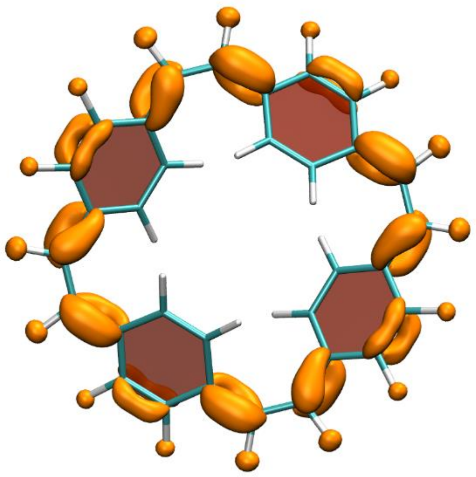 | 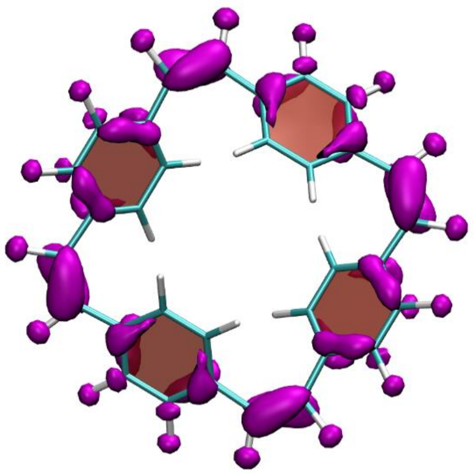 |

**BCyc$^{4-}$**

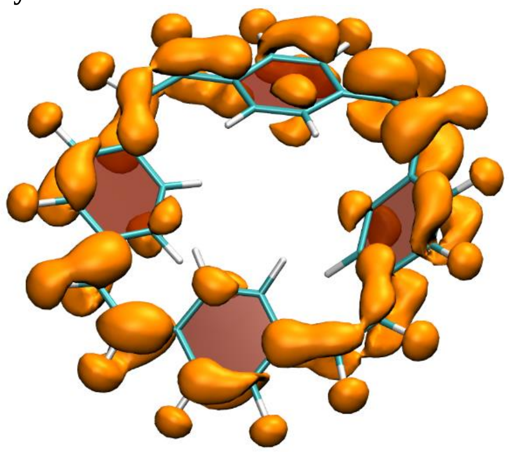

**BCyc$^{4+}$**

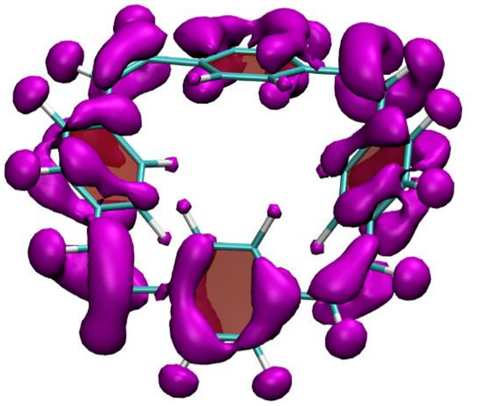

**BCyc$^{4-}$ (top)**

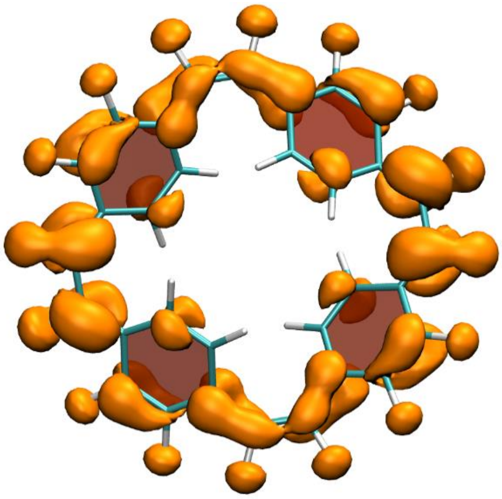

**BCyc$^{4+}$ (top)**

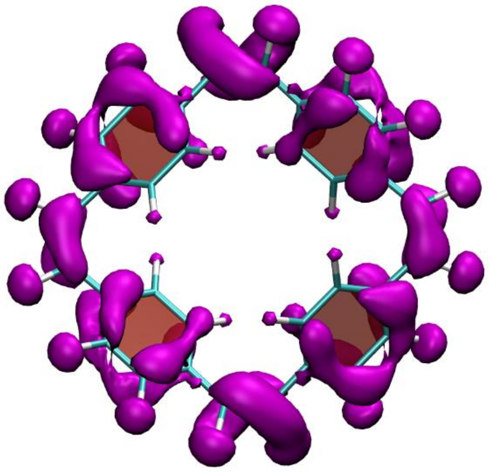

**BCyc$^{6-}$**

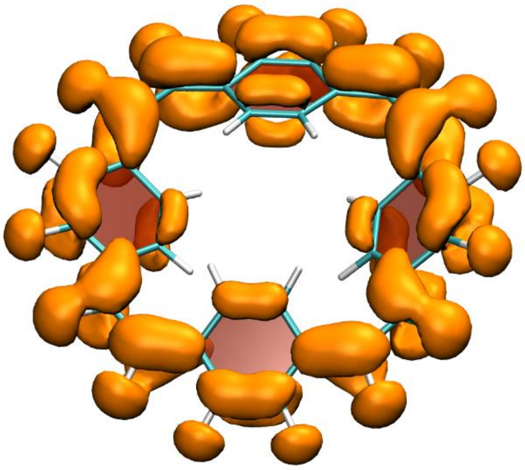

**BCyc$^{6+}$**

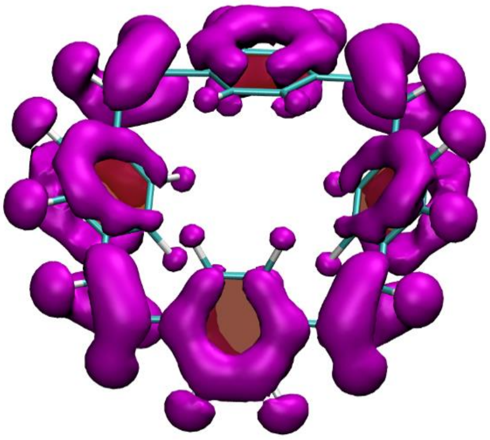

**BCyc$^{6-}$ (top)**

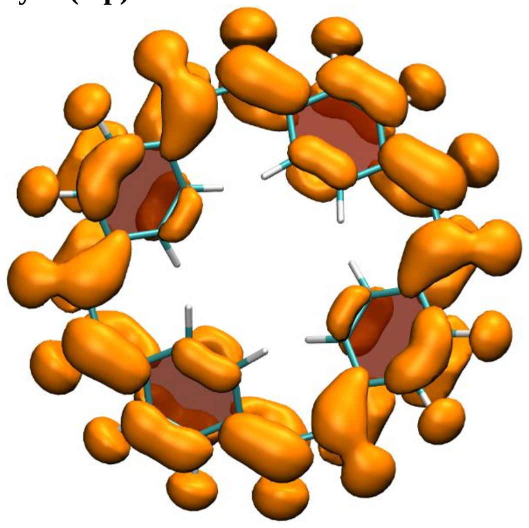

**BCyc$^{6+}$ (top)**

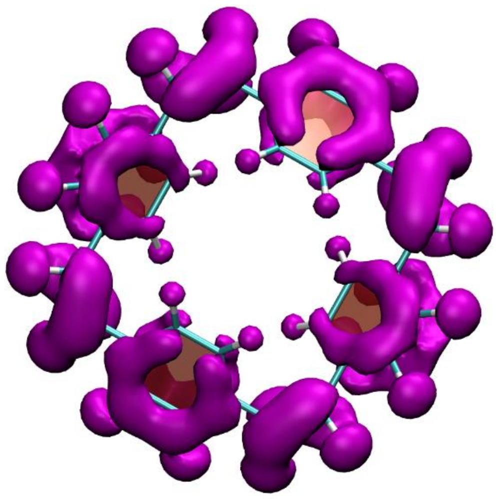

### 6.5.2 Electron density difference plots of BCyc-Et

Isovalues: -0.003 *e* for anions, +0.003 *e* for cations.

**BCyc-Et$^{2-}$**

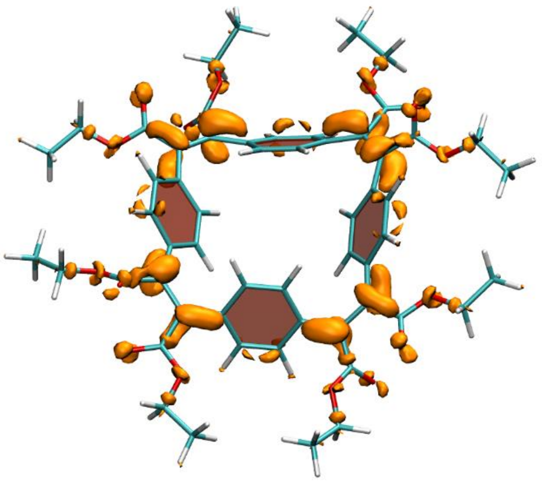

**BCyc-Et$^{2+}$**

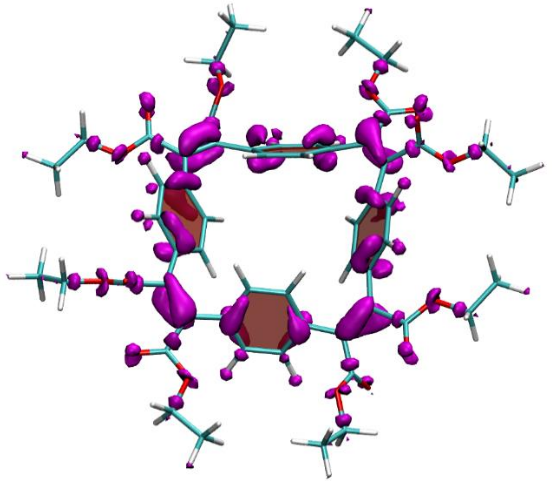

**BCyc-Et$^{2-}$ (top)**

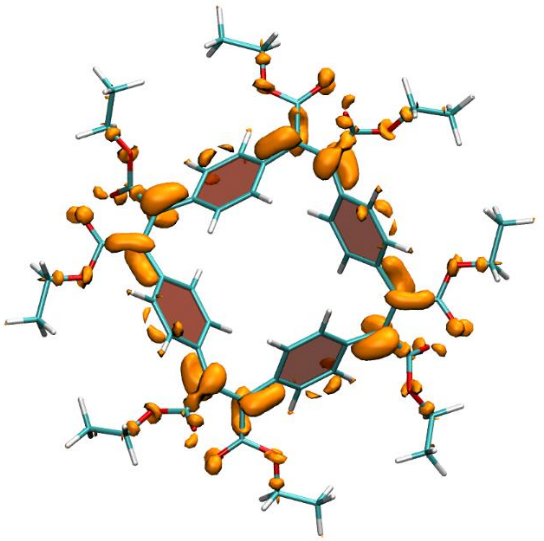

**BCyc-Et$^{2+}$ (top)**

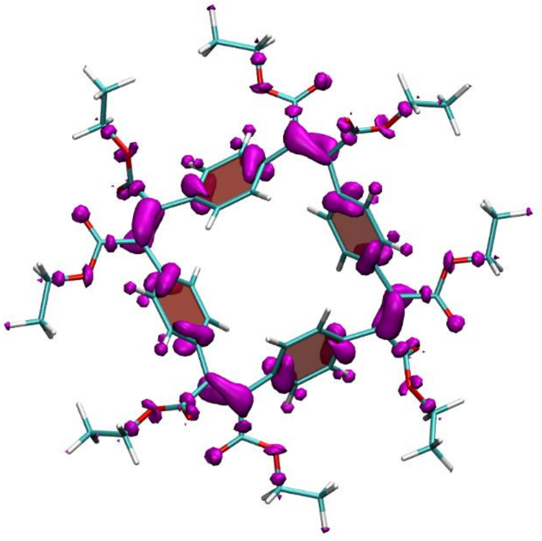

**BCyc-Et$^{4-}$**

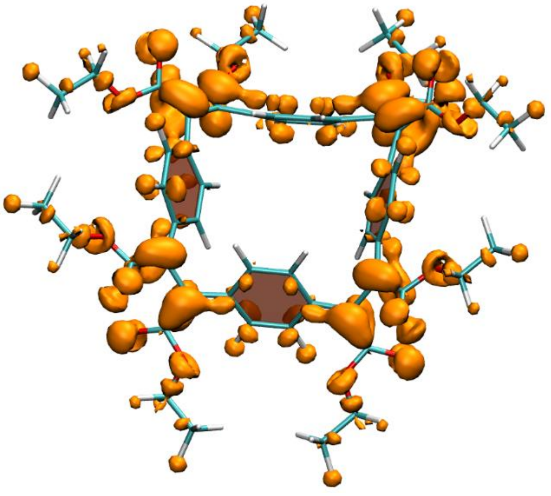

**BCyc-Et$^{4+}$**

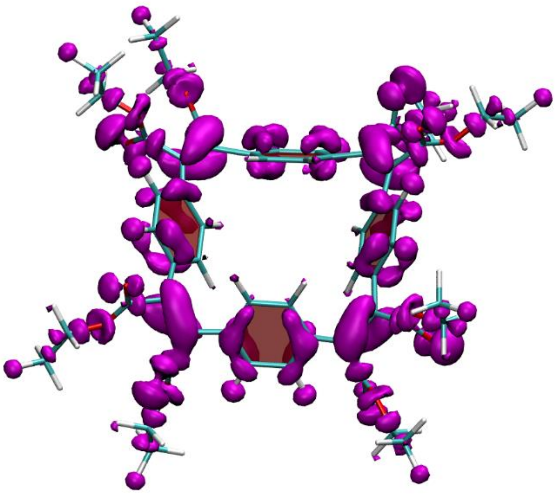

**BCyc-Et$^{4-}$ (top)**

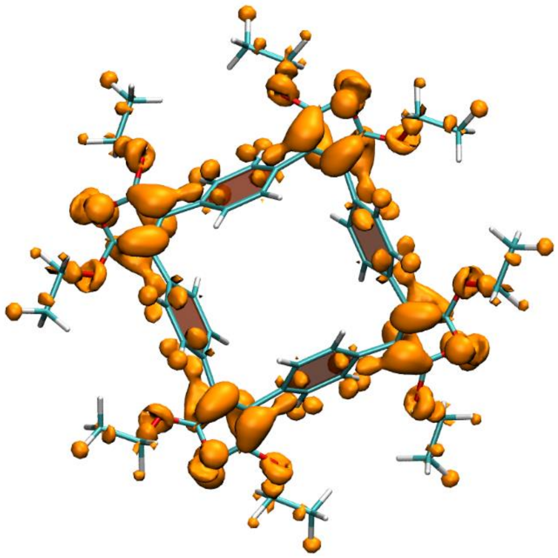

**BCyc-Et$^{4+}$ (top)**

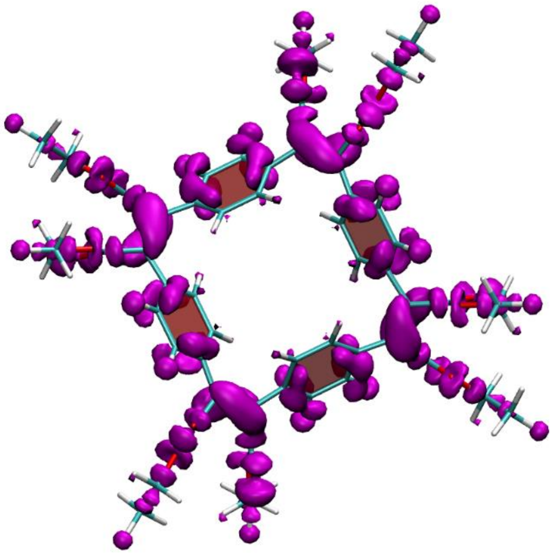

**BCyc-Et$^{6-}$**

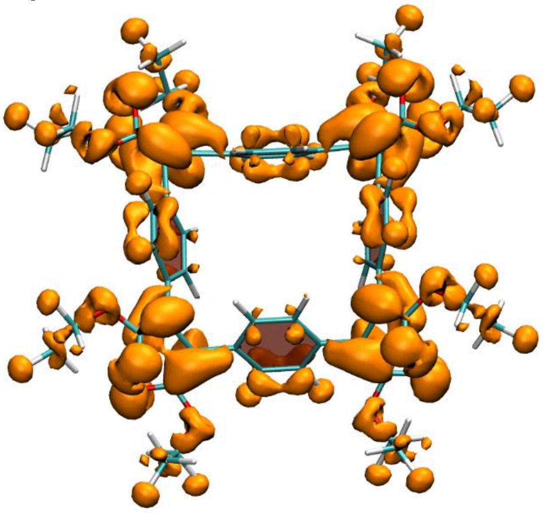

**BCyc-Et$^{6+}$**

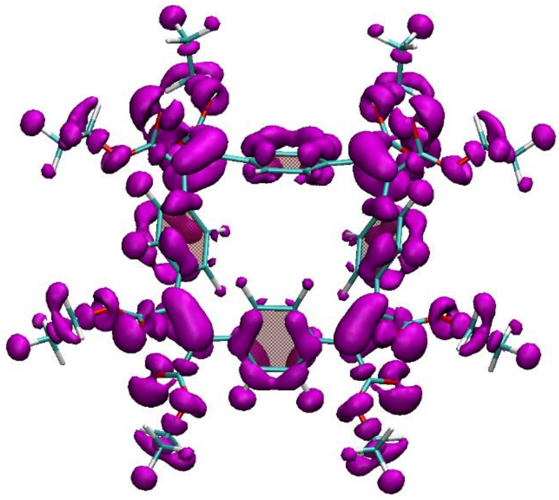

| **BCyc-Et$^{6-}$ (top)** | **BCyc-Et$^{6+}$ (top)** |
|---|---|
| 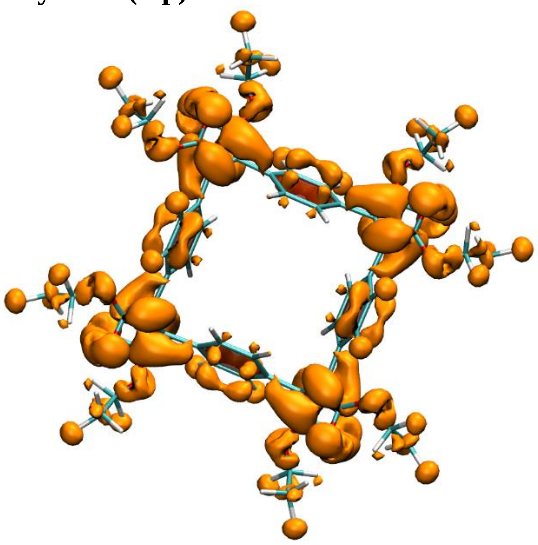 | 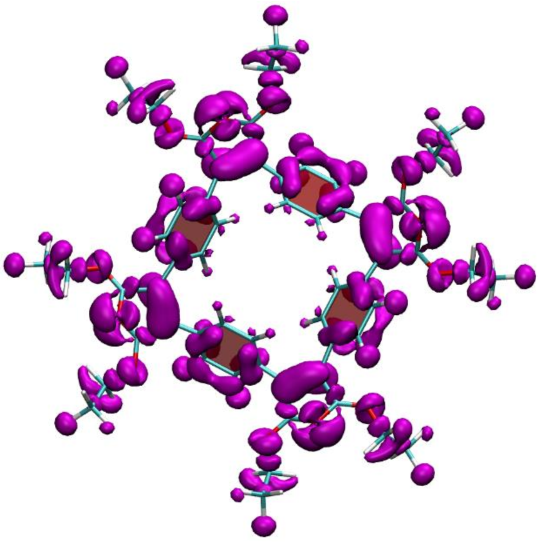 |

## 6.6 Anisotropy of the induced current density (ACID)

### 6.6.1 ACID plots of BCyc in different charge and spin states

| **BCyc** | **BCyc (triplet)** |
|---|---|
| 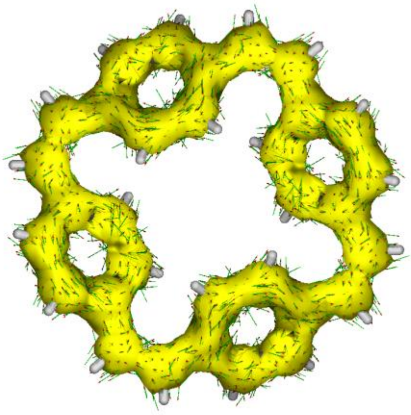 | 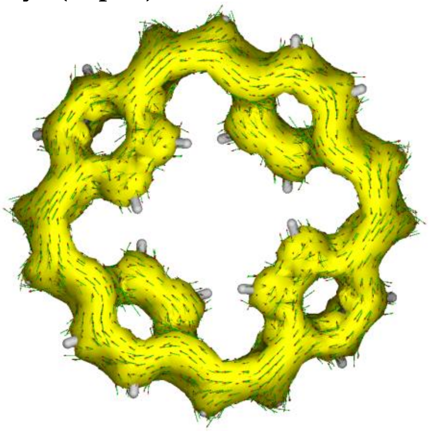 |
| **BCyc$^{2-}$** | **BCyc$^{2+}$** |
| 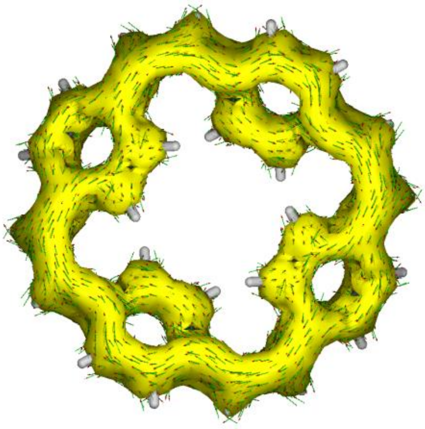 | 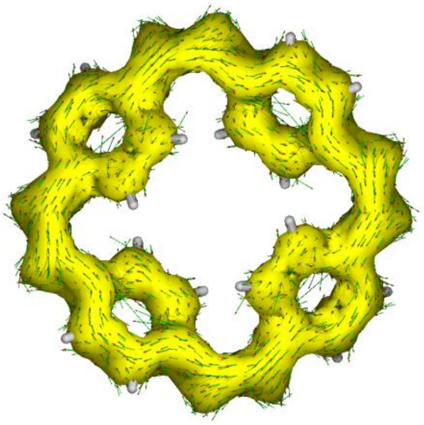 |

**BCyc$^{4-}$**

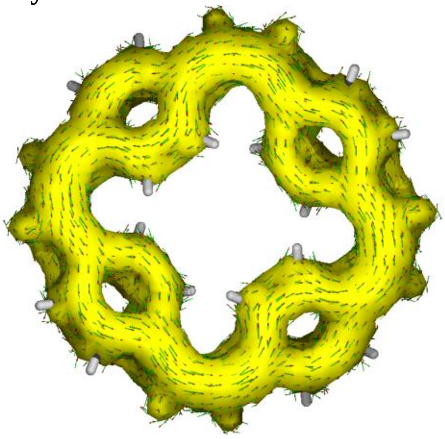

**BCyc$^{4+}$**

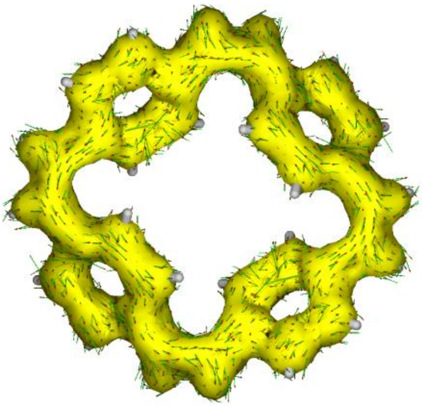

**BCyc$^{4-}$ (triplet)**

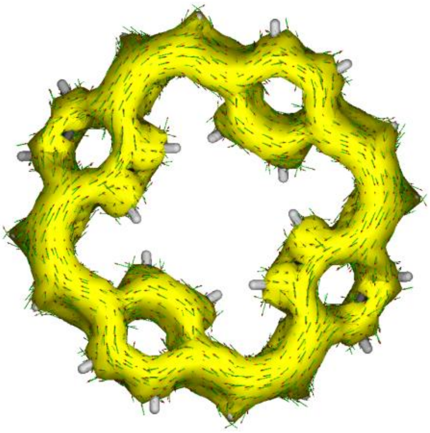

**BCyc$^{4+}$ (triplet)**

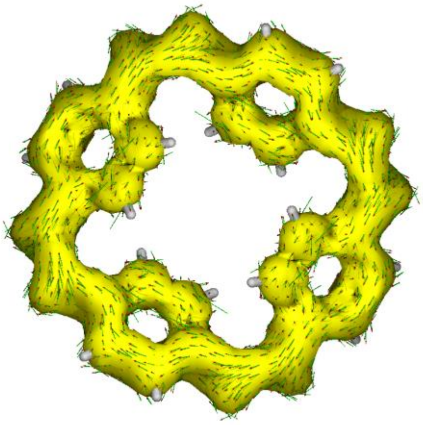

**BCyc$^{6-}$**

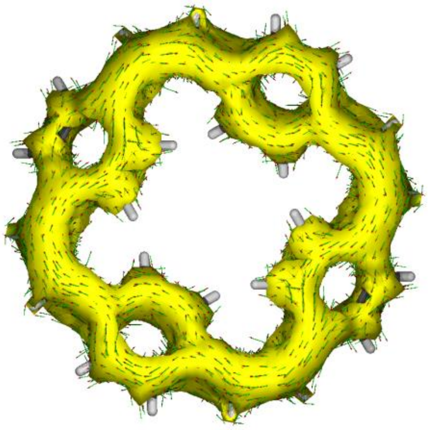

**BCyc$^{6+}$**

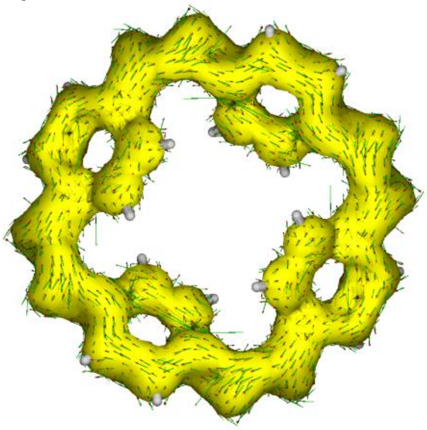

### 6.6.2 ACID plots of BCyc-Et in different charge and spin states

**BCyc-Et**

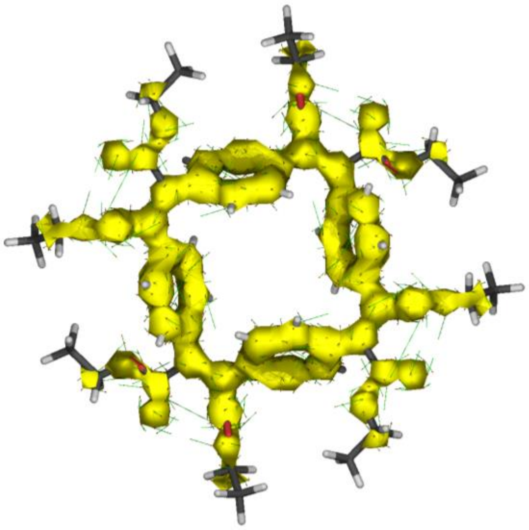

**BCyc-Et (triplet)**

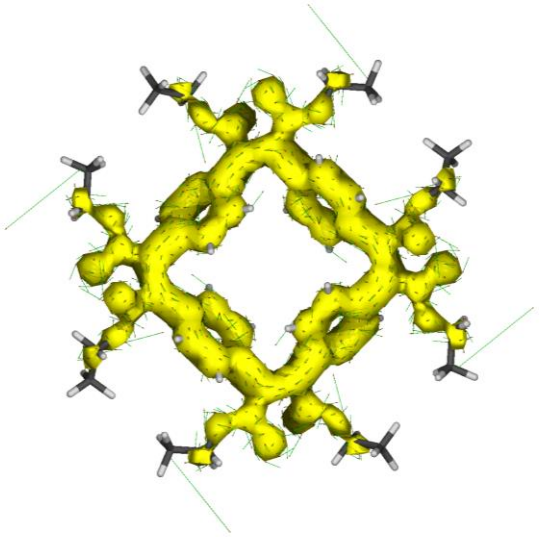

**BCyc-Et$^{2-}$**

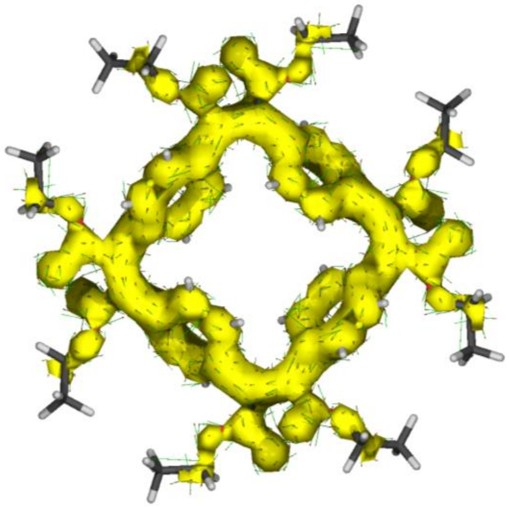

**BCyc-Et$^{2+}$**

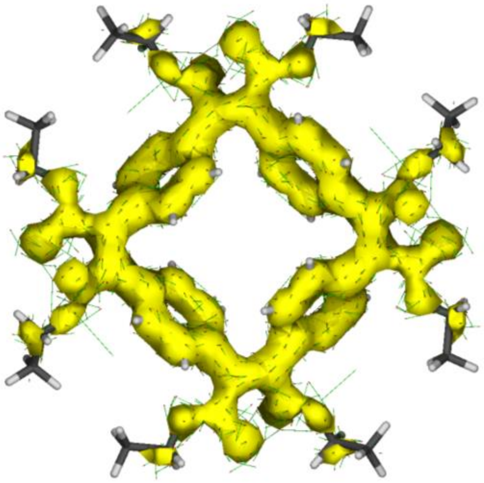

**BCyc-Et$^{4-}$**

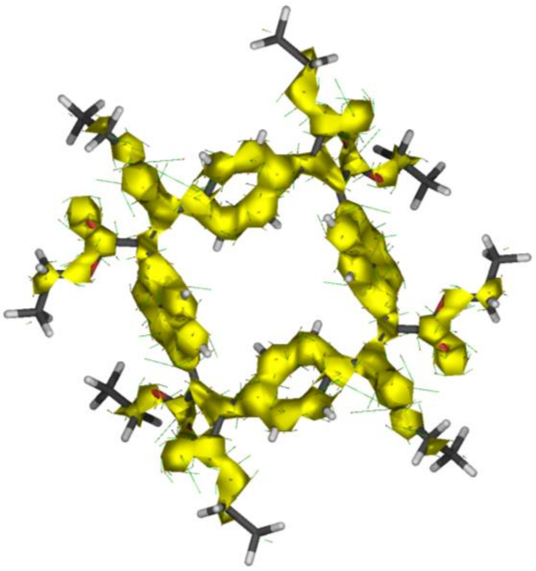

**BCyc-Et$^{4+}$**

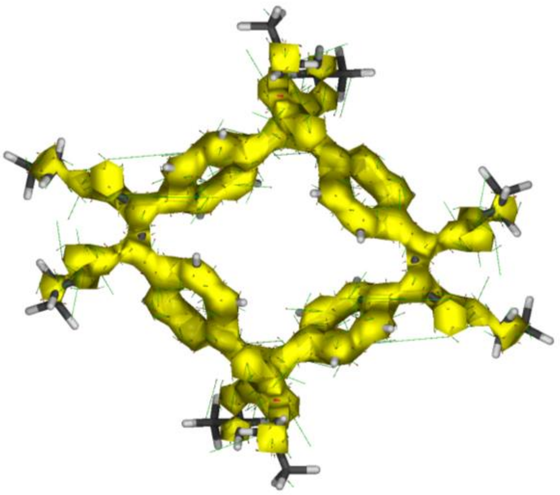

| **BCyc-Et$^{4-}$ (triplet)** | **BCyc-Et$^{4+}$ (triplet)** |
|---|---|
| 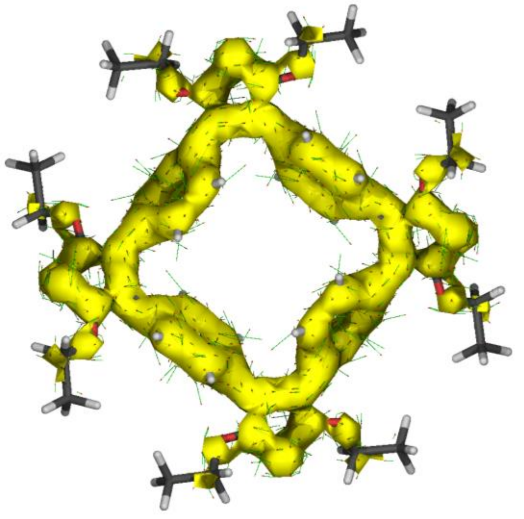 | 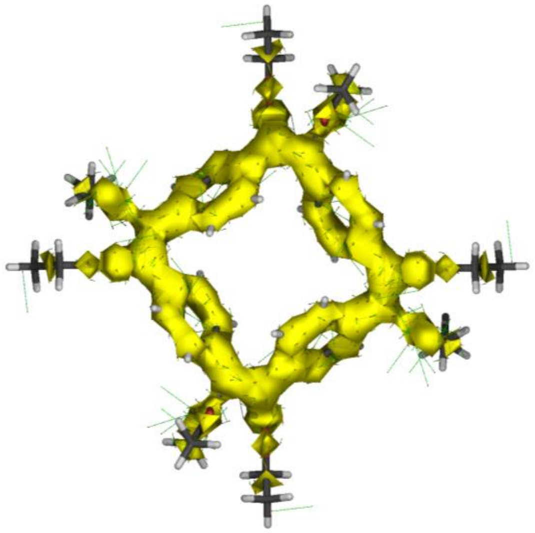 |
| **BCyc-Et$^{6-}$** | **BCyc-Et$^{6+}$** |
| 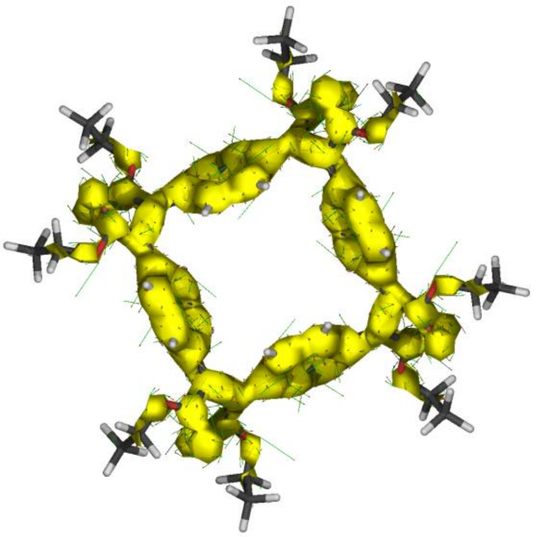 | 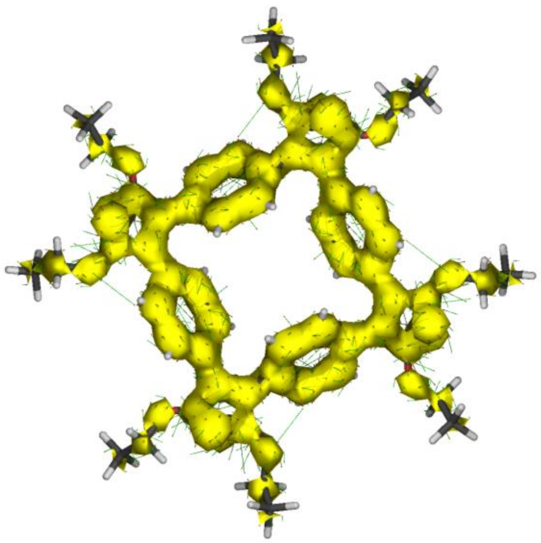 |

# 7 References


1. Kang, H.; Shin, H.; Kim, B.; Park, J., Position Effect Based on Anthracene Core for OLED Emitters, *J. Nanosci. Nanotechnol.* **2016,** *16* (3), 3045-3048. doi:10.1166/jnn.2016.11056
2. Robert, A.; Dechambenoit, P.; Hillard, E. A.; Bock, H.; Durola, F., Non-planar oligoarylene macrocycles from biphenyl, *Chem. Commun.* **2017,** *53* (84), 11540-11543. doi:10.1039/C7CC06798D
3. Moreira, T. S.; Ferreira, M.; Dall'armellina, A.; Cristiano, R.; Gallardo, H.; Hillard, E. A.; Bock, H.; Durola, F., Tetracarboxy-Functionalized [8]-, [10]-, [12]-, and [14]Phenacenes, *Eur. J. Org. Chem.* **2017,** *2017* (31), 4548-4551. doi:10.1002/ejoc.201700893
4. Girotto, E.; Ferreira, M.; Sarkar, P.; Bentaleb, A.; Hillard, E. A.; Gallardo, H.; Durola, F.; Bock, H., Plank-Shaped Column-Forming Mesogens with Substituents on One Side Only, *Chem. - Eur. J.* **2015,** *21* (20), 7603-7610. doi:10.1002/chem.201500048

5. Itoh, T.; Nomura, S.; Ohtake, M.; Yoshida, T.; Uno, T.; Kubo, M.; Kajiwara, A.; Sada, K.; Miyata, M., Molecular Oxygen Insertion Polymerization into Crystals of Tetrakis(alkoxycarbonyl)quinodimethanes, *Macromolecules* **2004,** *37* (22), 8230-8238. doi:10.1021/ma035997f
6. Naulet, G.; Robert, A.; Dechambenoit, P.; Bock, H.; Durola, F., Monoprotection of Arylene-Diacetic Acids Allowing the Build-Up of Longer Aromatic Ribbons by Successive Perkin Condensations, *Eur. J. Org. Chem.* **2018,** *2018* (5), 619-626. doi:10.1002/ejoc.201701499
7. Robert, A.; Dechambenoit, P.; Bock, H.; Durola, F., A carboxyfunctionalized (24)-1,6-pyrenophane-tetraene by glyoxylic Perkin condensation, *Can. J. Chem.* **2017,** *95* (4), 450-453. doi:10.1139/cjc-2016-0585
8. Wang, R.; Shi, K.; Cai, K.; Guo, Y.; Yang, X.; Wang, J.-Y.; Pei, J.; Zhao, D., Syntheses of polycyclic aromatic diimides via intramolecular cyclization of maleic acid derivatives, *New J. Chem.* **2016,** *40* (1), 113-121. doi:10.1039/C5NJ01849H
9. Eder, S.; Yoo, D.-J.; Nogala, W.; Pletzer, M.; Santana Bonilla, A.; White, A. J. P.; Jelfs, K. E.; Heeney, M.; Choi, J. W.; Glöcklhofer, F., Switching between Local and Global Aromaticity in a Conjugated Macrocycle for High-Performance Organic Sodium-Ion Battery Anodes, *Angew. Chem., Int. Ed.* **2020,** *59* (31), 12958-12964. doi:10.1002/anie.202003386
10. Mussari, L.; Postigo, M.; Lafuente, C.; Royo, F. M.; Urieta, J. S., Viscosity Measurements for the Binary Mixtures of 1,2-Dichloroethane or 1,2-Dibromoethane with Isomeric Butanols, *J. Chem. Eng. Data* **2000,** *45* (1), 86-91. doi:10.1021/je990211n
11. Jarusuwannapoom, T.; Hongrojjanawiwat, W.; Jitjaicham, S.; Wannatong, L.; Nithitanakul, M.; Pattamaprom, C.; Koombhongse, P.; Rangkupan, R.; Supaphol, P., Effect of solvents on electro-spinnability of polystyrene solutions and morphological appearance of resulting electrospun polystyrene fibers, *Eur. Polym. J.* **2005,** *41* (3), 409-421. doi:10.1016/j.eurpolymj.2004.10.010
12. Bernal-García, J. M.; Guzmán-López, A.; Cabrales-Torres, A.; Estrada-Baltazar, A.; Iglesias-Silva, G. A., Densities and Viscosities of (N,N-Dimethylformamide + Water) at Atmospheric Pressure from (283.15 to 353.15) K, *J. Chem. Eng. Data* **2008,** *53* (4), 1024-1027. doi:10.1021/je700671t
13. Muhuri, P. K.; Hazra, D. K., Density and Viscosity for Propylene Carbonate + 1,2-Dimethoxyethane at 298.15, 308.15, and 318.15 K, *J. Chem. Eng. Data* **1994,** *39* (2), 375-377. doi:10.1021/je00014a041
14. Chernyak, Y., Dielectric Constant, Dipole Moment, and Solubility Parameters of Some Cyclic Acid Esters, *J. Chem. Eng. Data* **2006,** *51* (2), 416-418. doi:10.1021/je050341y
15. Abdiaziz, K.; Salvadori, E.; Sokol, K. P.; Reisner, E.; Roessler, M. M., Protein film electrochemical EPR spectroscopy as a technique to investigate redox reactions in biomolecules, *Chem. Commun.* **2019,** *55* (60), 8840-8843. doi:10.1039/C9CC03212F
16. Fliegl, H.; Taubert, S.; Lehtonen, O.; Sundholm, D., The gauge including magnetically induced current method, *Phys. Chem. Chem. Phys.* **2011,** *13* (46), 20500-20518. doi:10.1039/C1CP21812C
17. *TURBOMOLE V7.4 2019, a development of University of Karlsruhe and Forschungszentrum Karlsruhe GmbH, 1989-2007, TURBOMOLE GmbH, since 2007; available from http://www.turbomole.com.*
